# Unlocking the Secrets of Roman Chamomile (*Anthemis nobilis* L.) Essential Oil: Structural Elucidation and Acute Toxicity of New Esters

**DOI:** 10.3390/molecules31020256

**Published:** 2026-01-12

**Authors:** Niko S. Radulović, Marko Z. Mladenović

**Affiliations:** 1 Department of Chemistry, Faculty of Sciences and Mathematics, University of Niš, Višegradska 33, 18000 Niš, Serbia; 2 Department of Sciences and Mathematics, State University of Novi Pazar, Vuka Karadžića 9, 36300 Novi Pazar, Serbia

**Keywords:** *Anthemis nobilis* L., Roman chamomile, essential oil, chemical composition, esters

## Abstract

To address gaps in the characterization of Roman chamomile (*Anthemis nobilis* L., Asteraceae)—an ethnobotanically and commercially important species—we profiled its essential oil (EO), focusing on esters that are incompletely characterized or unreported. Comprehensive GC-MS of two commercial EOs and their chromatographic fractions, combined with synthesis and co-injection of reference compounds, enabled the identification of 190 constituents. We uncovered a homologous series of angelates, tiglates, and senecioates by partial-ion-current (PIC) screening (*m*/*z* 55, 83, 100, 101), augmented by co-injection and NMR confirmation. Among these EO constituents, four esters, methallyl 3-methylbutanoate (**6h**), methallyl senecioate (**3h**), 3-methylpentyl 2-methylbutanoate (**5c**), and 5-methylhexyl angelate (**2g**) are reported here as new natural products and previously unreported compounds in the literature. Selected methacrylates and related α,β-unsaturated esters underwent model Michael additions to methanethiol (generated in situ from dimethyl disulfide and NaBH_4_), confirming their thiol-acceptor reactivity. In an *Artemia salina* assay, the EO and most esters were non-toxic; methacrylates showed only low toxicity at the highest concentrations. These results refine the chemical map of *A. nobilis* EO and highlight specific ester families for future mechanistic and biological evaluation.

## 1. Introduction

*Anthemis nobilis* L. (syn. *Chamaemelum nobile* (L.) All., Roman chamomile), alongside *Matricaria chamomilla* L. (German chamomile), with which it often shares the common vernacular name “chamomile,” represents one of the oldest and most widely utilized medicinal plants, recognized for its long-standing ethnopharmacological relevance [1]. Its therapeutic applications have been well recognized for centuries in ancient civilizations such as Greece, Rome, and Egypt [2], and the plant continues to maintain global recognition in both traditional and modern medicine [1]. Historically limited to small-scale domestic production, Roman chamomile has gained considerable commercial interest in recent years. This growing demand has led to its systematic cultivation in several countries, including Belgium, France, Italy, Germany, the United Kingdom, Egypt, Algeria, Hungary, Poland, Bulgaria, and Argentina [3]. In Serbia, Roman chamomile occurs naturally in the southeastern region, where it is still collected from the wild for export, use as herbal tea, or processing into phytopharmaceutical products. However, a notable increase in its commercial cultivation has been observed in recent years [3], as demonstrated by the establishment of organized plantations such as Siempreviva Oils and Tamnjanica Eco Plantations near the city of Niš.

The essential oil (EO) of Roman chamomile is typically obtained from aerial parts of the flowering plant, with fresh flowers yielding approximately 1.0% (*w*/*w*) and dried flowers up to 1.6% (*w*/*w*) of the oil [3]. Chemical composition of the aerial parts and flowers of Roman chamomile, encompassing both volatile and non-volatile constituents, has been extensively investigated over the past several decades [1,4,5]. Based on available data, it can be estimated that almost 100 different volatiles were found in the EO of Roman chamomile, predominantly by hemiterpenoid acid esters (angelates/tiglates/senecioates) alongside monoterpene hydrocarbons/oxygenated monoterpenes, with their identification predominantly achieved using gas chromatography–mass spectrometry [4]. However, regrettably, some of these compounds were undoubtedly misidentified. For example, Farkas et al. (2003) reported the identification of butyl angelate and butyl tiglate, with a difference in their retention indices of only 2 RI units (1091 and 1093, respectively), which raises serious doubts about the reliability of these identifications [6]. Generally, in the literature, the difference in retention indices (RI) between angelate and tiglate esters, when the alcohol moiety is the same, is approximately 50 units [7]. While the RI value reported for butyl angelate (RI = 1091 [6]) is consistent with literature data [8], the value reported for butyl tiglate (RI = 1093) deviates significantly from the expected literature value for this compound on the same GC column (RI = 1136 [9]). This discrepancy strongly suggests a misidentification. Work by Filipović et al. [3] regrettably suffers from similar inaccuracies (they identified isoamyl angelate at RI = 1143 and isoamyl tiglate at RI = 1148). Additionally, ten years ago, Tadrent et al. (2016) reported the identification of vinyl 2,2-dimethylbutanoate and glycidyl methacrylate as major constituents of the essential oil, accounting for relative abundances of 24.2% and 9.9%, respectively [10]. However, the accuracy of these identifications is highly questionable, and we believe that they most likely represent either a contaminant or a misidentification. Furthermore, certain EO constituents, particularly those with possible structural isomers that have similar retention index values and nearly indistinguishable mass-spectral fragmentation patterns (e.g., the cases of propyl methacrylate, isobutyl isovalerate, 2-butenyl angelate, and detected tiglate of C_6_ saturated alcohols), were reported to be only tentatively identified in the studies of Antonelli and Fabbri (1998) or Bicchi et al. (1987) [11,12]. Definitive structural elucidation of such compounds requires their isolation or the application of complementary identification techniques, such as chemical synthesis followed by gas chromatographic co-injection experiments. Their identification remains relevant from both phytochemical and commercial perspectives, as it contributes to a deeper understanding of the plant’s aromatic profile and its potential applications in flavor and fragrance industries, as well as, pharmacy.

Thus, as part of our ongoing efforts to comprehensively characterize essential oils through chromatographic fractionation, chemical synthesis, and NMR spectroscopy, we undertook a reinvestigation of the chemical composition of a commercially available Roman chamomile essential oil. In the present study, we report the comprehensive analysis of two commercial essential oil samples obtained from the flowering aerial parts of Roman chamomile populations originating from Serbia. A total of 190 volatile constituents were identified, among which over one hundred are reported for the first time as components of Roman chamomile essential oil including four new natural products. It is particularly surprising that, according to the available literature, a series of detected methacrylates have been identified as natural products exclusively in Roman chamomile EO. Roman chamomile has been traditionally utilized for a wide range of therapeutic purposes and is classified as “generally recognized as safe” by the United States Food and Drug Administration (FDA), supporting its use in food and medicinal products [1]. The presence of methacrylates, compounds for which one could expect some level of toxicity, prompted us to investigate both the safety and possible beneficial pharmacological properties of these naturally occurring esters, and, by extension, the safety profile of *A. nobilis*-based herbal preparations. To this end, we conducted a preliminary acute toxicity assessment using an *Artemia salina* bioassay, evaluating selected methacrylates and structurally related esters from a prepared sublibrary to gain initial insight into structure–activity relationships. In addition, the reactivity of methacrylates, tiglates, and angelates toward thiol groups was investigated, highlighting their potential to act as Michael acceptors, a property that may be pharmacologically relevant and warrants further investigation.

## 2. Results and Discussion

### 2.1. Chemical Composition of A. nobilis Essential Oil and Essential Oil Fractions

**Figure 1:**
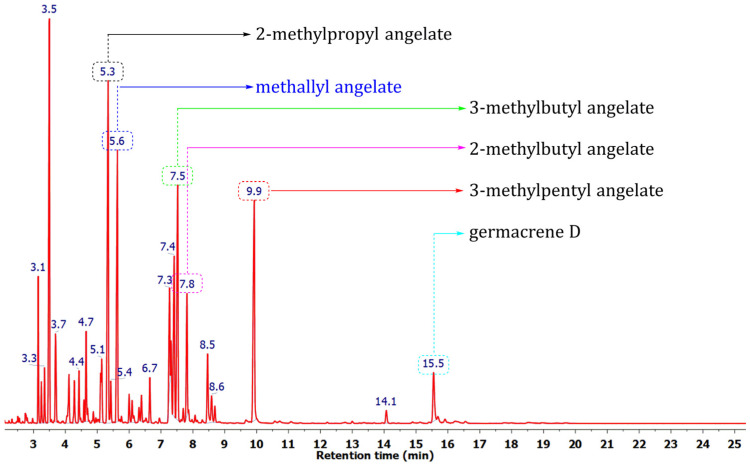
Typical TIC (total ion current) chromatogram of Roman chamomile (*A. nobilis*) essential oil.

Our investigation of the constituents of *Anthemis nobilis* essential oil (EO) has involved a comprehensive analysis of their composition using GC-MS, followed by preparative chromatographic separation, synthesis of pure compounds, and the application of a suite of structural elucidation techniques, including IR, 1D, and 2D NMR spectroscopy. Two commercially available samples (A and B, Table 1) were a generous gift from the company Siempreviva Oils (Niš, Serbia). Sample A was produced from plant material collected near Bovan Lake (Soko Banja) in 2017, whereas sample B was isolated from aerial parts collected from the herb plantation in Tamjanica, Niš, Serbia in 2018. The results of the GC-MS analysis of these samples and their chromatographic fractions are summarized in Table 1, and a representative chromatogram is shown in Figure 1.

The majority of EO constituents were identified by comparing their retention indices and mass spectra to literature data and GC-MS spectral libraries, and in some cases by GC co-injection experiments using synthesized/purchased authentic standards. Across the analyzed samples, this led to the successful identification of more than 190 compounds, and, up to this point, already this represents an increase of more than 50% of the known natural products found in Roman chamomile [4]. The identified constituents represented 97.6–99.6% of the total detected areas of the GC chromatogram of EO and EO chromatographic fractions samples, dominated by hemiterpenoid acid esters (angelates/tiglates/senecioates) alongside monoterpene hydrocarbons/oxygenated monoterpenes, with minor sesquiterpenes. 2-Methylpropyl 2-methylpropanoate (2.6–2.9%), 2-methylpropyl methacrylate (**1a**; 3.0–3.1%), α-pinene (6.1–9.2%), 2-methylbutyl 2-methylpropanoate (2.2–2.3%), 2-methylpropyl angelate (11.9–13.9%), methallyl angelate (**2h**; 8.5–9.3%), 3-methylpentyl methacrylate (**1c**; 1.7–3.5%), 3-methylbutyl angelate (5.4–5.9%), 2-methylbutyl angelate (9.3–9.5%), pinocarvone (2.7–2.9%), prenyl angelate (2.3–2.8%), 3-methylpentyl angelate (**2c**; 9.9–10.2%), and germacrene D (2.7–3.2%) represented the major EO constituents (Table 1). A comparison of the identified constituents with the previously published data revealed that the composition of the essential oil is rather complex, with a seemingly high variance in component presence and abundance depending on locality, harvest time, and growing season [4,6,13,14]. Nevertheless, a consistent feature across samples is the pronounced dominance of angelic acid esters.

Considering the observed compositional variation and the fact that *A. nobilis* essential oil originating from Serbia has been previously analyzed only once [3], a comprehensive investigation of its chemical profile was deemed necessary. Accordingly, an in-depth analysis was undertaken to identify as many unknown constituent GC peaks as possible. The identity of several constituents could not be determined by the analysis of MS and RI data alone. Subsequent efforts led to the identification of several additional, previously unreported constituents of *A. nobilis* essential oil, all classified as esters. Their structural elucidation was made possible by targeted synthesis, as detailed in the following sections. Two detected peaks (RI = 1017 and 1204) did not yield satisfactory matches in spectral libraries; however, fragmentation patterns visible in their mass spectra suggested that they most likely correspond to the methallyl and (iso)hexyl esters of isomeric pentanoic acids, respectively (Figure S82). The first compound exhibited a base peak at *m*/*z* 57 (C_4_H_9_^·+^), followed by a prominent one at *m*/*z* 85 (C_5_H_9_O^·+^). Additionally, the MS of the mentioned compounds displayed an ion at *m*/*z* 55, characteristic of a C_4_H_7_^·+^ fragment. The mass spectrum of the second compound additionally featured an abundant ion at *m*/*z* 84 (C_6_H_12_^·+^), indicative of an (iso)hexyl moiety in the alcoholic part of the molecule. Based on the RI values and MS data, the first compound was tentatively identified as methallyl ester of 2-methylbutanoic or 3-methylbutanoic acids, whereas the second was proposed to be 3-methylpentyl 3-methylbutanoate (**6c**). This assignment was further supported by the notable presence of structurally related 3-methylpentyl esters, such as isobutanoate, methacrylate (**1c**), and angelate (**2c**), whose identification was readily confirmed based on MS and RI data in both essential oil samples. The synthesis of the aforementioned tentatively identified methallyl and 3-methylpentyl esters was carried out using standard Steglich esterification protocols. Subsequent GC co-injection experiments with the synthesized reference compounds confirmed the proposed structural assignments. According to a SciFinder search of the Chemical Abstracts Service (CAS) database conducted on 12 November 2025, complemented by searches in NIST, MassFinder, and our in-house library, at the time of this investigation, the synthesized esters, 3-methylpentyl 2-methylbutanoate (**5c**) and methallyl 3-methylbutanoate (**6h**), represented new compounds and newly identified natural products. Although methallyl 2-methylbutanoate (**5h**) and 3-methylpentyl 3-methylbutanoate (**6c**) had previously been reported as natural products, in this study they were synthesized and spectroscopically characterized for the first time, including full MS, NMR, and IR data.

**Figure 2:**
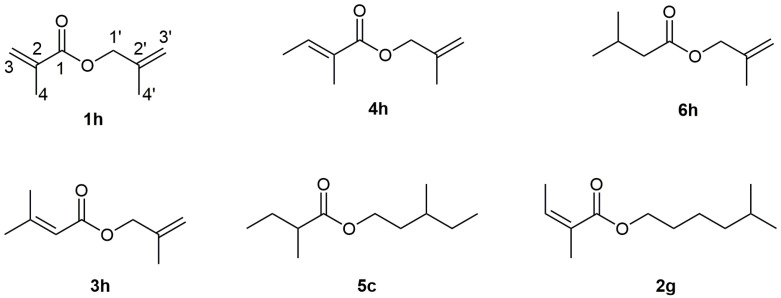
Structures of selected rare natural products (methallyl methacrylate (**1h**) with carbon-atom numbering and methallyl tiglate (**4h**)) and newly identified natural products (methallyl 3-methylbutanoate (**6h**), methallyl senecioate (**3h**), 3-methylpentyl 2-methylbutanoate (**5c**), and 5-methylhexyl angelate (**2g**)).

To identify additional constituents exhibiting MS fragmentation patterns analogous to those of the previously identified methallyl esters, a partial ion current (PIC) chromatogram was generated for both the essential oil samples and ester-rich fraction F4, monitoring the *m*/*z* 55 ion over time. This approach led to the detection of five additional ‘methallyl ester-like’ compounds, three of which were straightforwardly identified as isobutanoate, methacrylate (**1h**), and angelate (**2h**) based on available MS and RI data from the literature (Figure 2). Their identification was further confirmed through GC co-injection experiments using synthesized standards and EO samples. Although these esters have previously been reported as components of *A. nobilis* essential oil, this study presents their complete spectral characterization for the first time, including both 1D and 2D NMR techniques. The mass spectra of the remaining two methallyl esters closely resembled that of methallyl angelate but exhibited significantly higher RI values (1091 and 1106, respectively) compared to the angelate (RI = 1062). Based on our prior experience with esters of this type [15], we hypothesized that, in addition to methallyl angelate (**2h**), the analyzed samples also contained methallyl tiglate (**4h**) and methallyl senecioate (**3h**) (Figure 2). This assumption was confirmed through synthesis and GC co-chromatographic analysis, which verified the identities of the detected compounds as methallyl senecioate (**3h**; RI = 1091) and methallyl tiglate (**4h**; RI = 1106). A SciFinder search of the Chemical Abstracts Service (CAS) database conducted on November 12, 2025, further confirmed that methallyl senecioate (**3h**) represents both new natural product and previously unreported chemical entity.

The identification of the above-mentioned tiglate and senecioate esters further prompted us to conduct an additional screening of the samples using partial ion current (PIC) chromatograms, specifically by monitoring characteristic ions for angelates, tiglates, and senecioates (*m*/*z* 55, 83, 100, and 101). The PIC chromatograms indicated the presence of an entire series of angelates, tiglates, and senecioates, comprising a total of 44 constituents (Figure S77). The identity of the majority of these compounds was ultimately confirmed through co-chromatography of the EO samples with synthesized standards of homologous series of angelates, tiglates, and senecioates (Table 1).

However, the identification of isomeric hexyl esters proved challenging due to their nearly identical mass spectra and very similar RI values. This necessitated the synthesis of a small synthetic library of angelates, tiglates, and senecioates derived from the most structurally plausible isomeric hexanols, primarily those featuring a single methyl branching. The synthetic library comprised esters of angelic (**2**), senecioic (**3**), and tiglic (**4**) acids with 2-methylpentan-1-ol (**b**), 3-methylpentan-1-ol (**c**), 4-methylpentan-1-ol (**d**), and n-hexanol (**e**). All synthesized esters (12 in total) were fully characterized, most of them for the first time, by a comprehensive set of spectroscopic techniques, including 1D and 2D NMR, as well as MS and IR. Co-chromatographic analysis of the synthesized standards with the essential oil samples confirmed the presence of 4-methylpentyl angelate (**2d**), 3-methylpentyl angelate (**2c**), 3-methylpentyl tiglate (**4c**), hexyl angelate (**2e**), and hexyl tiglate (**4e**) as authentic constituents of the essential oil. For another peak identified in the aforementioned PIC chromatograms, the mass spectral fragmentation pattern, together with the retention index value (RI = 1349), which was approximately 100 units higher than that of 4-methylpentyl angelate and 36 units lower than that of heptyl angelate, suggested the presence of a branched isomer of heptyl angelate, specifically 5-methylhexyl angelate (**2g**). The synthesis of 5-methylhexyl, 4-methylhexyl, and n-heptyl angelates, followed by co-chromatographic runs with the essential oil sample, confirmed the identity of the compound as 5-methylhexyl angelate (**2g**; syn. isoheptyl angelate), representing a newly identified natural product.

### 2.2. NMR Data

Selected synthesized esters, some of which were identified as new natural products, were subjected to further structural elucidation using NMR spectroscopy. Below is an analysis of 1D (^1^H and ^13^C, including ^13^C spectra without heteronuclear decoupling, as well as DEPT-90 and DEPT-135) and 2D (gradient ^1^H–^1^H COSY, NOESY, HSQC, and HMBC) NMR spectra for four representative methallyl esters from the EO samples: methacrylate (**1h**), 3-methylbutanoate (**6h**), tiglate (**4h**), and senecioate (**3h**) (Figure 2), while the NMR spectral analysis of the remaining esters was performed in the same manner. The ^1^H and ^13^C NMR spectra of the examined compounds displayed the expected number of signals, as detailed in the Section 3 and Supplementary Materials. As expected, all four compounds exhibited nearly identical signals originating from the methallyl moiety. HSQC spectral data indicated that in all four compounds, the proton signals observed at approximately 4.5 ppm (appearing as a broad singlet) are directly bonded to a carbon atom at around 67.4 ppm, thus confirming their assignment to the C-1′ position (Figure 2). Additional HMBC correlations of these protons with carbon atoms signals, e.g., at 167.06, 140.02, 112.64, and 19.52 ppm in methallyl methacrylate, allowed their identification as the C-1, C-2′, C-3′, and C-4′ atoms, respectively. Furthermore, HSQC correlations of proton signals at 5.00 (pseudo triplet of quintets, *J* = 1.6, 0.8 Hz, 1H), 4.95–4.93 ppm (multiplet, 1H), and 1.78 ppm (broad doublet of doublets, *J* = 1.6, 0.8 Hz, 3H) further confirmed the assignment of C-3′ and C-4′ carbon atoms, as well as their corresponding protons at positions 3′ and 4′, respectively. Additional 2D NMR correlations that confirmed the structure of the synthesized methallyl methacrylate included HMBC interactions between the proton signals at 6.15 ppm (doublet of quartets, *J* = 1.6, 1.0 Hz, 1H) and 5.59 ppm (pseudo quintet, *J* = 1.6 Hz, 1H) with the carbon signals at 167.06, 136.27, and 18.34 ppm, assigned to C-1, C-2, and C-4, respectively. In the case of the isovalerate, tiglate, and senecioate derivatives, the structural assignments for the signals originating from the acid moieties were fully consistent with the reported NMR data for esters of this type in the literature [15].

### 2.3. Identification of a Series of Methacrylic Acid Esters and the Synthesis of Their Sulfur-Containing Adducts by DMDS

Another noteworthy group of esters, methacrylates, which have previously been sporadically identified as plant metabolites, drew our further attention. In addition to 2-methylpropyl methacrylate (**1a**) and 3-methylpentyl methacrylate (**1c**), two of the major constituents of the essential oil, whose tentative identifications were confirmed through comparison of their MS and RI data with those available in the literature [6] and further validated by synthesis and GC co-injection experiments, a partial ion current (PIC) chromatogram generated using characteristic methacrylate fragment ions enabled the detection of an additional series of nine “methacrylate-like” compounds in the essential oil. The synthesis of a small library of methacrylates followed by GC co-chromatography with the essential oil samples confirmed the presence of the following compounds as constituents of the essential oil: propyl, methallyl (**1h**), butyl, 3-methylbutyl, 2-methylbutyl, pentyl, prenyl, 4-methylpentyl (**1d**), and benzyl methacrylates (Table 1). Based on a comprehensive literature survey, 4-methylpentyl methacrylate (**1d**) was identified for the first time as a natural product. In the present study, this compound, along with the major essential oil constituents 2-methylpropyl (**1a**) and 3-methylpentyl methacrylate (**1c**), was fully characterized for the first time using a combination of spectroscopic techniques, including MS, IR, 1D, and 2D NMR. It is particularly surprising that, according to the available literature, the detected methacrylates have been identified as natural products exclusively in the essential oil of *A. nobilis* [6,11,12,14,16,17,18,19,20,21,22]. This finding may hold significant chemotaxonomic value, as these compounds could serve as reliable chemical markers for this taxon. This is especially relevant given the frequent taxonomic misidentification of *A. nobilis* due to its close morphological resemblance with other *Anthemis* and *Matricaria* species. However, although the presence of methacrylates in *A. nobilis* essential oil has been confirmed on multiple occasions, the question remains as to whether these compounds are genuine natural products or artifacts formed during the extraction process. Nevertheless, there is currently insufficient evidence to support or dispute this hypothesis. Further research is warranted to clarify whether methacrylates are indeed natural constituents of the essential oil or by-products generated during the extraction procedure.

Interactions between unsaturated conjugated carbonyl compounds (Michael acceptors) and sulfhydryl groups in biogenic molecules, such as thiol groups in proteins and glutathione, play a crucial role in various biological processes and therapeutic mechanisms. For example, studies have shown that α,β-unsaturated carbonyl compounds can react with thiol groups of cysteine residues in proteins, leading to modifications that affect the function of transcription factors such as Nrf2 and NF-κB. These modifications can result in the induction of anti-inflammatory enzymes such as heme oxygenase-1 (HO-1) or the inhibition of pro-inflammatory proteins like iNOS, TNF, and IL-6 [23]. Additionally, studies have demonstrated that Michael acceptors can form adducts with glutathione (GSH), whereby the thiol group of GSH covalently binds to the β-carbon of the Michael acceptor [24]. This interaction may affect GSH homeostasis and has implications for cellular detoxification processes [24]. Also, in the context of antimalarial activity, some morphinan derivatives containing Michael acceptor moieties have shown the ability to bind to thiol groups, which correlates with their efficacy against *Plasmodium falciparum*. These properties were confirmed through NMR experiments that demonstrated adduct formation with 1-propanethiol [25]. These findings highlight the importance of interactions between unsaturated conjugated carbonyls and sulfhydryl groups in biological systems, both in physiological processes and in the development of new therapeutic agents.

**Figure 3:**
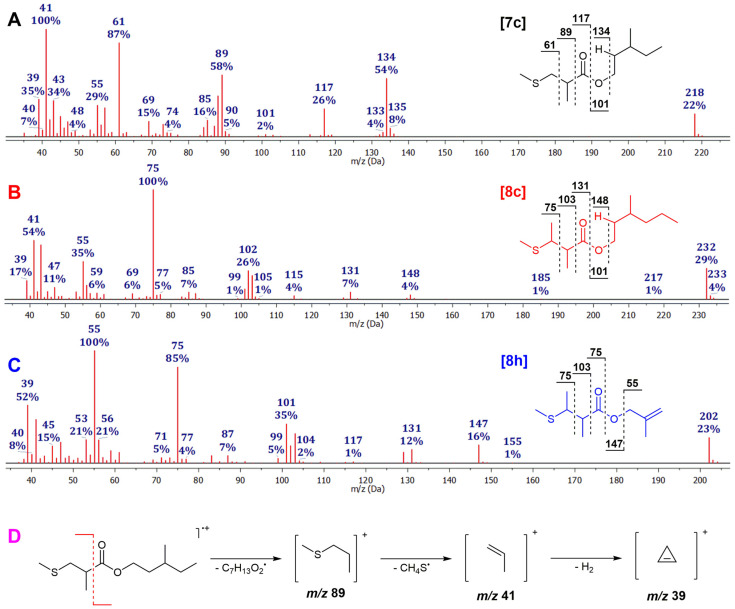
Mass spectra of the synthesized 3-methylpentyl 2-methyl-3-(methylthio)propanoate (**A**), 3-methylpentyl 2-methyl-3-(methylthio)butanoate (**B**), and 2-methylallyl 2-methyl-3-(methylthio)butanoate (**C**), together with the proposed electron ionization fragmentation pathways leading to the intense ions at *m*/*z* 41 and 39 (**D**).

The detected methacrylates represent ideal Michael acceptors candidates, potentially capable of exerting the aforementioned biological effects through interactions with thiol (syn. sulfhydryl) groups in biological systems. In addition to the potential biological activity of the synthesized esters, their substantial presence in the essential oil of *A. nobilis* (e.g., in total more than 11% of sample A, Table 1) suggests that the entire essential oil may also exhibit such activity. Therefore, before time-consuming and highly expensive in vivo and in vitro biological assays, we decided to preliminarily test this hypothesis using a simplified model reaction. We used 3-methylpentyl methacrylate (**1c**) as a test compound, a methacrylate ester identified in a relatively high amount in the essential oil (1.7–3.5%), in a reaction with an excess of methanethiol, generated in situ by the reaction of dimethyl disulfide with sodium borohydride (Figure 3). This reaction proceeded rapidly and quantitatively, yielding a stable adduct (3-methylpentyl 2-methyl-3-(methylthio)propanoate; **7c**) that was isolated and fully characterized by MS and NMR (see Section 3). The observed interaction of the methacrylate with methanethiol highlights its potential biological activity, which will be further investigated in our future studies. In addition to methacrylates, the essential oil also contains significant amounts of angelates, tiglates, and senecioates that also possess α,β-unsaturated carbonyl groups and may behave analogously to methacrylates. In support of this, we observed that the synthesized 3-methylpentyl tiglate (**4c**) and methallyl angelate (**2h**), both present in the essential oil samples, also reacted with methanethiol, although at a slower rate and with lower yield compared to methacrylates. These results support the hypothesis that the mentioned esters may exhibit potential biological activity as Michael acceptors; however, it should be emphasized that the observed reactivity reflects chemical behavior in a simplified model system, and any inference of biological significance remains hypothetical until validated by dedicated biological assays.

### 2.4. Brine Shrimp Lethality

The acute toxicity of the essential oil sample and the selected synthesized compounds (3-methylpentyl methacrylate (**1c**), 3-methylpentyl 2-methylbutanoate (**5c**), 3-methylpentyl 3-methylbutanoate (**6c**), methallyl 2-methylbutanoate (**5h**), methallyl 3-methylbutanoate (**6h**), methallyl angelate (**2h**), and 4-methylpentyl methacrylate (**1d**)) was tested with an *Artemia salina* acute toxicity assay, as described previously [26]. Additionally, the complete series of synthesized isomeric hexyl esters (2-methylpentyl, 3-methylpentyl, 4-methylpentyl, and hexyl) of angelic (**2b**–**2e**), tiglic (**4b**–**4e**), and senecioic (**3b**–**3e**) acid was tested to evaluate the impact of structural variation on acute toxicity. The obtained results demonstrated that, within the tested concentration range of 3.9–125 mg/L, the evaluated sample compounds exhibited either no toxicity or only low levels of toxicity compared to the positive control. The LC_50_ values determined for SDS, as the positive control, were consistent with those reported in the literature [27]. The highest tested concentration was limited by the solubility of the essential oil or pure compounds in the assay medium.

The whole series of synthesized isomeric hexyl angelates, hexyl tiglates, and hexyl senecioates, 3-methylpentyl 2-methylbutanoate, 3-methylpentyl 3-methylbutanoate, methallyl 2-methylbutanoate, methallyl 3-methylbutanoate, methallyl angelate, as well as the tested essential-oil sample turned out to be non-toxic to *Artemia salina* (the mortality for the highest tested concentrations of the mentioned samples was less than 10%, as in the case of the negative control [27]). Among the tested compounds, only the methacrylates (3-methylpentyl and 4-methylpentyl methacrylate) exhibited low toxicity in the *A. salina* acute toxicity assay. Mortality for the highest tested concentrations of the mentioned methacrylates after 24 h was 40% (for this reason, we could not calculate LC_50_ with an acceptable degree of confidence), whereas the LC_50_ after 48 h was 125 mg/L (0.73 mM). The tested set of compounds allowed for a clear observation of the correlation between structure and toxicity. It was evident that the presence of the methacrylate moiety in the ester structure contributed to the observed toxic effect in the *A. salina* assay. In contrast, structural modifications in the alcohol moiety such as methyl branching, variation in the position of the methyl group, or the presence of unsaturation, as seen in the case of methallyl esters, did not influence toxicity within the tested concentration range. This screening assay was included only to provide an initial hazard profile for newly characterized esters and does not substitute for mammalian toxicology.

## 3. Materials and Methods

### 3.1. Chemicals

Solvents (HPLC grade) and chemicals (dimethyl disulfide (DMDS), 4-(dimethylamino)pyridine (DMAP), *N*,*N*’-dicyclohexylcarbodiimide (DCC), 2-methylpropan-1-ol (**a**), 2-methylpentan-1-ol (**b**), 3-methylpentan-1-ol (**c**), 4-methylpentan-1-ol (**d**), hexan-1-ol (**e**), 3-chloro-2-methylprop-1-ene (methallyl chloride), 2-methylbutanoic (**5**), 3-methylbutanoic (**6**), methacrylic (**1**; 2-methylpropenoic acid), angelic (**2**; (*Z*)-2-methyl-2-butenoic), tiglic (**4**; (*E*)-2-methyl-2-butenoic), and senecioic (**3**; 3-methyl-2-butenoic) acids) were of analytical grade, commercially available (TCI (Paris, France), Sigma-Aldrich (St. Louis, MO, USA), and Merck (Darmstadt, Germany)), and were used as received unless stated otherwise. Hydrocarbon mixtures utilized for the determination of retention indices were purchased from Sigma-Aldrich (St. Louis, MO, USA). Deuterated chloroform (CDCl_3_) was also acquired from Sigma-Aldrich (St. Louis, MO, USA). Silica gel 60 (particle size distribution 40–63 μm) was used for column or dry-flash chromatography, whereas precoated Al silica gel plates (Kieselgel 60 F_254_, 0.2 mm, Merck, Darmstadt, Germany) were used for analytical TLC analyses.

### 3.2. General Experimental Procedures

The spots on TLC were visualized by UV light (254 nm) and by spraying with 50% (*v*/*v*) aq. H_2_SO_4_ or 10% (*w*/*v*) ethanolic solution of phosphomolybdic acid, followed by heating. IR measurements (ATR-attenuated total reflectance) were carried out using a Thermo Nicolet model 6700 FTIR instrument (Waltham, MA, USA). The ^1^H (including ^1^H NMR selective homonuclear decoupling experiments), ^13^C (with and without heteronuclear decoupling) nuclear magnetic resonance (NMR) spectra, distortionless enhancement by polarization transfer (DEPT90 and DEPT135), and 2D (NOESY, and gradient ^1^H–^1^H COSY, HSQC, and HMBC) NMR spectra were recorded on a Bruker Avance III 400 MHz NMR spectrometer (Fällanden, Switzerland; ^1^H at 400 MHz, ^13^C at 101 MHz) equipped with a 5–11 mm dual ^13^C/^1^H probe head. All NMR spectra were measured at 25 °C in CDCl_3_ with tetramethylsilane (TMS) as the internal standard. Chemical shifts are reported in ppm (*δ*) and referenced to TMS (*δ*_H_ = 0 ppm) in ^1^H NMR spectra and/or to solvent protons (CDCl_3_: *δ*_H_ = 7.26 ppm and *δ*_C_ = 77.16 ppm) in ^13^C and heteronuclear 2D spectra. The acquired NMR experiments, both 1D and 2D, were recorded using standard Bruker built-in pulse sequences. GC-MS analyses were performed on an HP 6890N GC with a DB-5MS capillary column (30 m × 0.25 mm, 0.25 µm; Agilent, Santa Clara, CA, USA) coupled to a 5975B MSD (EI, 70 eV). Injector and interface temperatures were 250 and 300 °C. The oven program was 70 → 290 °C at 5 °C·min^−1^, then 10 min isothermal. Helium was the carrier gas (pulsed split, 40:1; 1.0 µL injection of 1.0 mg/mL solutions). Flow was 1.5 mL·min^−1^ for the first 0.5 min, then 1.0 mL·min^−1^. Spectra were acquired over *m*/*z* 35–650 with a 0.32 s scan time.

### 3.3. Component Identification

Essential oil constituents were identified by comparison of their linear retention indices (RI) relative to the homologous series of *n*-alkanes on a DB-5MS column with literature data and their mass spectra with those of authentic standards, as well as those from Wiley 11, NIST17, MassFinder 2.3, and a homemade MS library with spectra corresponding to pure substances, NMR analysis of isolated compounds and, wherever possible, by co-injection with an authentic sample [28].

### 3.4. Fractionation of the A. nobilis Essential Oil

Essential oil samples of *A. nobilis* (samples A and B; Table 1) were generously provided by Siempreviva Oils (Niš, Serbia). Sample A was obtained from aerial parts collected near Bovan Lake, Soko Banja, in 2017, whereas sample B was distilled from aerial parts harvested in 2018 from a plantation in Tamjanica (Niš, Serbia). A portion of the pooled essential oil samples A and B (2.0 g) was subjected to dry flash chromatography, which resulted in a total of six different fractions (59, 8, 1597, 142, 20, and 125 mg, respectively), obtained using hexane, 2%, 3%, 4%, and 5% (*v*/*v*) diethyl ether in hexane as eluents for the first five fractions, and pure diethyl ether as the eluent for the final fraction. A gradient of hexane-diethyl ether, from 100:0 to 0:100 (*v*/*v*), was employed for the chromatography and the mentioned fractions were immediately analyzed by GC-MS upon solvent removal in vacuo.

### 3.5. Synthesis of 4-Methylhexan-1-ol and 5-Methylhexan-1-ol

A solution of dimethyl malonate (10.0 g; 75.75 mmol), 1-bromo-2-methylbutane or 1-bromo-3-methylbutane (1.2 eq; 13.7 g; 90.91 mmol), and anhydrous potassium carbonate (4 eq; 41.8 g; 0.3 mol) in dry acetone (100 mL) were refluxed for 4 h and additionally stirred for 20 h at room temperature [29]. The reaction was quenched by the addition of water (75 mL). Acetone was evaporated and the remaining water layer was extracted with Et_2_O (4 × 75 mL). The organic layers were dried with anhydrous MgSO_4_ and concentrated under reduced pressure to give a crude product that, according to the GC-MS analysis, represented pure dimethyl 2-(3-methylbutyl)- or 2-(2-methylbutyl)malonate. Afterward, the crude products, an aqueous solution of NaOH (4 eq), and EtOH was refluxed for 4 h. EtOH was evaporated, and the water layer was washed with Et_2_O (2 × 25 mL), then acidified with 1 M HCl and extracted with Et_2_O (5 × 75 mL). Combined ether extracts were concentrated under reduced pressure to yield the crude 2-isopentyl or 2-(2-methylbutyl)malonic acid that was subsequently decarboxylated by heating at 210 °C for 2 h under nitrogen. The obtained dark oil was purified by dry-flash chromatography on SiO_2_ using mixtures of the increasing polarity of hexane and EtOAc as the eluent to give pure 5-methyl and 4-methylhexanoic acids [29].

Lithium aluminum hydride (6.7 eq) was added to a dry 250 mL round-bottom flask with anhydrous, freshly distilled THF (80 mL). The content of the flask was cooled in an ice bath, and the portion of synthesized acids was slowly added under vigorous stirring. After the addition of acid, the reaction mixture was stirred at room temperature for 30 min and additionally 5 h under reflux. Then, the reaction mixture was cooled in an ice bath, and saturated sodium sulfate solution was added dropwise, the ice bath was removed, anhydrous sodium sulfate and THF were added, and the reaction mixture was stirred overnight. The reaction mixture was filtered through Celite and after evaporation of the solvent a colorless oil was obtained. The obtained oil was purified by dry-flash chromatography on SiO_2_ using mixtures of the increasing polarity of hexane and Et_2_O as the eluent to give pure 5-methyl and 4-methylhexanols. Their mass spectral data were in agreement with those reported in the literature [30].

### 3.6. Synthesis of Esters

Esters were prepared according to the following general Steglich procedure: a solution of alcohols (200 mg), the appropriate carboxylic acid (1.1 eq), DMAP (0.3 eq), and DCC (1 eq) in dichloromethane (20 mL) was stirred overnight at room temperature. The precipitated urea was filtered off and the filtrate was concentrated in vacuo. The resulting residue was subjected to SiO_2_ dry-flash chromatography, except in the case of angelates that were subjected to column chromatography, using *n*-hexane/Et_2_O mixtures of increasing polarity as the eluents. The purity of the ester fractions was evaluated by TLC and GC-MS.

2-Methylpropyl 2-methylprop-2-enoate (**1a**; syn. isobutyl methacrylate) [6]: Yield: 65%; RI = 930 (DB-5MS); IR (ATR, cm^−1^): 2958, 2930, 2876, 1716, 1639, 1454, 1403, 1378, 1297, 1158, 1055, 1013, 974, 937, 814; MS (EI), *m*/*z* (%) 93(4), 87(31), 70(6), 69(100), 57(11), 56(45), 55(6), 43(8), 41(74), 39(33); ^1^H NMR (CDCl_3_, 400 MHz): δ 6.11 (doublet of quartets, *J* = 1.6, 1.0 Hz, 1H, H-3a), 5.55 (pseudo quintet, *J* = 1.6 Hz, 1H, H-3b), 3.93 (doublet, *J* = 6.6 Hz, 2H, H-1′), 1.99 (nonet, *J* = 6.6 Hz, 1H, H-2′), 1.95 (doublet of doublets, *J* = 1.6, 1.0 Hz, 3H, H-4), 0.96 (doublet, *J* = 6.6 Hz, 6H, H-3′ and H-4′); ^13^C NMR (CDCl_3_, 101 MHz): δ 167.52 (C-1), 136.54 (C-2), 125.16 (C-3), 70.73 (C-1′), 27.78 (C-2′), 19.13 (C-3′ and C-4′), 18.34 (C-4).

3-Methylpentyl 2-methylprop-2-enoate (**1c**; syn. 3-methylpentyl methacrylate) [6]: Yield: 73%; RI = 1141 (DB-5MS); IR (ATR, cm^−1^): 2960, 2929, 2876, 1717, 1638, 1455, 1403, 1378, 1319, 1296, 1158, 1055, 1012, 973, 937, 814; MS (EI), *m*/*z* (%) 113(4), 87(18), 84(53), 70(6), 69(100), 57(8), 55(25), 53(4), 43(14), 41(65), 40(7), 39(35); ^1^H NMR (CDCl_3_, 400 MHz): δ 6.09 (doublet of quartets, *J* = 1.6, 1.0 Hz, 1H, H-3a), 5.54 (pseudo quintet, *J* = 1.6 Hz, 1H, H-3b), 4.24–4.13 (multiplet, 2H, H-1′), 1.94 (doublet of doublets, *J* = 1.6, 1.0 Hz, 3H, H-4), 1.76–1.67 (multiplet, 1H, H_a_-2′), 1.55–1.45 (overlapping peaks, 2H, H-3′ and H_b_-2′), 1.42–1.33 (multiplet, 1H, H_a_-4′), 1.27–1.15 (multiplet, 1H, H_b_-4′), 0.92 (doublet, *J* = 6.4 Hz, 3H, H-6′), 0.89 (pseudo triplet, *J* = 7.4 Hz, 3H, H-5′); ^13^C NMR (CDCl_3_, 101 MHz): δ 167.56 (C-1), 136.59 (C-2), 125.12 (C-3), 63.32 (C-1′), 35.10 (C-2′), 31.58 (C-3′), 29.42 (C-4′), 19.10 (C-6′), 18.34 (C-4), 11.25 (C-5′).

4-Methylpentyl 2-methylprop-2-enoate (**1d**; syn. 4-methylpentyl methacrylate) [31]: Yield: 75%; RI = 1136 (DB-5MS); IR (ATR, cm^−1^): 2956, 2871, 1717, 1638, 1468, 1453, 1320, 1295, 1158, 1012, 992, 937, 814; MS (EI), *m*/*z* (%) 127(6), 87(64), 84(35), 83(8), 70(7), 69(100), 59(10), 56(45), 55(15), 42(14), 41(96), 39(49); ^1^H NMR (CDCl_3_, 400 MHz): δ 6.10 (doublet of quartets, *J* = 1.6, 1.0 Hz, 1H, H-3a), 5.55 (pseudo quintet, *J* = 1.6 Hz, 1H, H-3b), 4.13 (triplet, *J* = 6.8 Hz, 2H, H-1′), 1.95 (doublet of doublets, *J* = 1.6, 1.0 Hz, 3H, H-4), 1.72–1.63 (multiplet, 2H, H-2′), 1.58 (nonet, *J* = 6.6 Hz, 1H, H-4′), 1.29–1.22 (multiplet, 2H, H-3′), 0.90 (doublet, *J* = 6.6 Hz, 6H, H-5′ and H-6′); ^13^C NMR (CDCl_3_, 101 MHz): δ 167.57 (C-1), 136.58 (C-2), 125.15 (C-3), 65.11 (C-1′), 35.09 (C-3′), 27.73 (C-4′), 26.53 (C-2′), 22.52 (C-5′ and C-6′), 18.35 (C-4).

2-Methylpentyl (*Z*)-2-methylbut-2-enoate (**2b**; syn. 2-methylpentyl angelate): Yield: 44%; RI = 1240 (DB-5MS); IR (ATR, cm^−1^): 2957, 2928, 2874, 1716, 1651, 1457, 1379, 1352, 1256, 1230, 1147, 1083, 1043, 986, 846; MS (EI), *m*/*z* (%) 184(1), 101(17), 100(100), 85(27), 84(19), 83(87), 69(12), 57(9), 56(27), 55(90), 53(18), 43(55), 42(11), 41(37); ^1^H NMR (CDCl_3_, 400 MHz): δ 6.05 (quartet of quartets, *J* = 7.2, 1.5 Hz, 1H, H-3), 4.04 (doublet of doublets, *J* = 10.8, 5.7 Hz, 1H, H_a_-1′), 3.93 (doublet of doublets, *J* = 10.8, 6.7 Hz, 1H, H_b_-1′), 1.99 (doublet of quartets, *J* = 7.2, 1.5 Hz, 3H, H-4), 1.90 (pseudo quintet, *J* = 1.5 Hz, 3H, H-5), 1.87–1.77 (multiplet, 1H, H-2′), 1.44–1.28 (overlapping peaks, 3H, H_a_-3′ and H-4′), 1.24–1.14 (multiplet, 1H, H_b_-3′), 0.96 (doublet, *J* = 6.7 Hz, 3H, H-6′), 0.90 (pseudo triplet, *J* = 7.2 Hz, 3H, H-5′); ^13^C NMR (CDCl_3_, 101 MHz): δ 168.27 (C-1), 137.42 (C-3), 128.17 (C-2), 69.11 (C-1′), 35.74 (C-3′), 32.34 (C-2′), 20.65 (C-4), 19.98 (C-4′), 17.07 (C-6′), 15.75 (C-5), 14.27 (C-5′).

3-Methylpentyl (*Z*)-2-methylbut-2-enoate (**2c**; syn. 3-methylpentyl angelate) [32]: Yield: 47%; RI = 1258 (DB-5MS); IR (ATR, cm^−1^): 2958, 2924, 2874, 1716, 1650, 1458, 1379, 1352, 1257, 1231, 1155, 1083, 1042, 973, 847; MS (EI), *m*/*z* (%) 184(5), 101(23), 100(82), 85(43), 84(23), 83(65), 69(29), 57(23), 56(19), 55(100), 53(21), 43(55), 42(7), 41(44); ^1^H NMR (CDCl_3_, 400 MHz): δ 6.04 (quartet of quartets, *J* = 7.2, 1.5 Hz, 1H, H-3), 4.24–4.12 (multiplet, 2H, H-1′), 1.98 (doublet of quartets, *J* = 7.2, 1.5 Hz, 3H, H-4), 1.89 (pseudo quintet, *J* = 1.5 Hz, 3H, H-5), 1.76–1.67 (multiplet, 1H, H_a_-2′), 1.54–1.43 (overlapping peaks, 2H, H-3′ and H_b_-2′), 1.42–1.32 (multiplet, 1H, H_a_-4′), 1.27–1.14 (multiplet, 1H, H_b_-4′), 0.92 (doublet, *J* = 6.4 Hz, 3H, H-6′), 0.89 (pseudo triplet, *J* = 7.4 Hz, 3H, H-5′); ^13^C NMR (CDCl_3_, 101 MHz): δ 168.28 (C-1), 137.33 (C-3), 128.12 (C-2), 62.70 (C-1′), 35.17 (C-2′), 31.56 (C-3′), 29.41 (C-4′), 20.63 (C-4), 19.08 (C-6′), 15.72 (C-5), 11.26 (C-5′).

4-Methylpentyl (*Z*)-2-methylbut-2-enoate (**2d**; syn. 4-methylpentyl angelate) [7]: Yield: 50%; RI = 1249 (DB-5MS); IR (ATR, cm^−1^): 2955, 2927, 2871, 1711, 1653, 1466, 1384, 1366, 1257, 1232, 1136, 1079, 1043, 991, 846; MS (EI), *m*/*z* (%) 184(8), 101(34), 100(100), 85(37), 84(8), 83(62), 69(12), 56(20), 55(80), 53(17), 43(71), 42(10), 41(43); ^1^H NMR (CDCl_3_, 400 MHz): δ 6.04 (quartet of quartets, *J* = 7.3, 1.5 Hz, 1H, H-3), 4.13 (triplet, *J* = 6.7 Hz, 2H, H-1′), 1.98 (doublet of quartets, *J* = 7.3, 1.5 Hz, 3H, H-4), 1.89 (pseudo quintet, *J* = 1.5 Hz, 3H, H-5), 1.72–1.63 (multiplet, 2H, H-3′), 1.58 (pseudo nonet, *J* = 6.6 Hz, 1H, H-4′), 1.30–1.23 (multiplet, 2H, H-2′), 0.90 (doublet, *J* = 6.6 Hz, 6H, H-5′ and H-6′); ^13^C NMR (CDCl_3_, 101 MHz): δ 168.25 (C-1), 137.29 (C-3), 128.16 (C-2), 64.51 (C-1′), 35.21 (C-2′), 27.74 (C-4′), 26.60 (C-3′), 22.52 (C-5′ and C-6′), 20.63 (C-4), 15.73 (C-5).

Hexyl (*Z*)-2-methylbut-2-enoate (**2e**; syn. hexyl angelate) [7]: Yield: 51%; RI = 1285 (DB-5MS); IR (ATR, cm^−1^): 2955, 2929, 2858, 1710, 1653, 1459, 1379, 1254, 1232, 1137, 1077, 1044, 733; MS (EI), *m*/*z* (%) 184(5), 101(23), 100(100), 85(13), 83(39), 82(19), 69(4), 56(11), 55(62), 53(13), 43(33), 42(7), 41(25); ^1^H NMR (CDCl_3_, 400 MHz): δ 6.04 (quartet of quartets, *J* = 7.3, 1.5 Hz, 1H, H-3), 4.14 (triplet, *J* = 6.7 Hz, 2H, H-1′), 1.98 (doublet of quartets, *J* = 7.3, 1.5 Hz, 3H, H-4), 1.89 (pseudo quintet, *J* = 1.5 Hz, 3H, H-5), 1.65 (pseudo quintet, *J* = 6.7 Hz, 3H, H-2′), 1.42–1.27 (overlapping peaks, 6H, H-3′–H-5′), 0.89 (pseudo triplet, *J* = 6.7 Hz, 3H, H-6′); ^13^C NMR (CDCl_3_, 101 MHz): δ 168.30 (C-1), 137.25 (C-3), 128.15 (C-2), 64.59 (C-1′), 31.43 (C-4′), 28.67 (C-2′), 25.74 (C-5′), 22.54 (C-3′), 20.62 (C-4), 14.01 (C-6′), 12.04 (C-5).

2-Methylpentyl 3-methylbut-2-enoate (**3b**; syn. 2-methylpentyl senecioate): Yield: 68%; RI = 1280 (DB-5MS); IR (ATR, cm^−1^): 2958, 2932, 2874, 1717, 1652, 1447, 1378, 1347, 1272, 1224, 1141, 1077, 1001, 850, 739; MS (EI), *m*/*z* (%) 101(18), 100(33), 84(16), 83(100), 82(8), 69(5), 56(10), 55(27), 53(8), 43(20), 41(18); ^1^H NMR (CDCl_3_, 400 MHz): δ 5.69 (heptet, *J* = 1.3 Hz, 1H, H-2), 3.98 (doublet of doublets, *J* = 10.8, 5.8 Hz, 1H, H_a_-1′), 3.87 (doublet of doublets, *J* = 10.8, 6.8 Hz, 1H, H_b_-1′), 2.17 (doublet, *J* = 1.3 Hz, 3H, H-5), 1.90 (doublet, *J* = 1.3 Hz, 3H, H-4), 1.87–1.76 (multiplet, 1H, H-2′), 1.44–1.26 (overlapping peaks, 3H, H_a_-3′ and H-4′), 1.20–1.12 (multiplet, 1H, H_b_-3′), 0.93 (doublet, *J* = 6.8 Hz, 3H, H-6′), 0.90 (triplet, *J* = 7.2 Hz, 3H, H-5′); ^13^C NMR (CDCl_3_, 101 MHz): δ 166.94 (C-1), 156.20 (C-3), 116.22 (C-2), 68.61 (C-1′), 35.74 (C-3′), 32.38 (C-2′), 27.38 (C-4), 20.20 (C-5), 19.98 (C-4′), 16.95 (C-6′), 14.27 (C-5′).

3-Methylpentyl 3-methylbut-2-enoate (**3c**; syn. 3-methylpentyl senecioate): Yield: 75%; RI = 1295 (DB-5MS); IR (ATR, cm^−1^): 2960, 2875, 1717, 1653, 1454, 1377, 1347, 1271, 1224, 1142, 1077, 977, 850; MS (EI), *m*/*z* (%) 184(2), 101(25), 100(33), 85(18), 84(23), 83(100), 82(12), 69(16), 57(12), 56(10), 55(43), 54(5), 53(13), 43(31), 41(29), 39(27); ^1^H NMR (CDCl_3_, 400 MHz): δ 5.67 (heptet, *J* = 1.3 Hz, 1H, H-2), 4.19–4.06 (multiplet, 2H, H-1′), 2.17 (doublet, *J* = 1.3 Hz, 3H, H-5), 1.89 (doublet, *J* = 1.3 Hz, 3H, H-4), 1.73–1.63 (multiplet, 1H, H_a_-2′), 1.53–1.43 (overlapping peaks, 2H, H-3′ and H_b_-2′), 1.42–1.32 (multiplet, 1H, H_a_-4′), 1.27–1.14 (multiplet, 1H, H_b_-4′), 0.91 (doublet, *J* = 6.4 Hz, 3H, H-6′), 0.88 (pseudo triplet, *J* = 7.4 Hz, 3H, H-5′); ^13^C NMR (CDCl_3_, 101 MHz): δ 166.85 (C-1), 156.25 (C-3), 116.21 (C-2), 62.14 (C-1′), 35.28 (C-2′), 31.49 (C-3′), 29.45 (C-4′), 27.37 (C-4), 20.17 (C-5), 19.05 (C-6′), 11.24 (C-5′).

4-Methylpentyl 3-methylbut-2-enoate (**3d**; syn. 4-methylpentyl senecioate): Yield: 77%; RI = 1289 (DB-5MS); IR (ATR, cm^−1^): 2952, 2874, 1717, 1654, 1454, 1377, 1347, 1271, 1225, 1142, 1077, 977, 850; MS (EI), *m*/*z* (%) 184(3), 101(43), 100(42), 85(19), 84(12), 83(100), 82(14), 69(7), 56(11), 55(34), 54(4), 53(11), 43(38), 41(30), 39(25); ^1^H NMR (CDCl_3_, 400 MHz): δ 5.68 (heptet, *J* = 1.3 Hz, 1H, H-2), 4.07 (triplet, *J* = 6.8 Hz, 2H, H-1′), 2.17 (doublet, *J* = 1.3 Hz, 3H, H-5), 1.89 (doublet, *J* = 1.3 Hz, 3H, H-4), 1.68–1.59 (multiplet, 2H, H-3′), 1.57 (pseudo nonet, *J* = 6.7 Hz, 1H, H-4′), 1.28–1.20 (multiplet, 2H, H-2′), 0.90 (doublet, *J* = 6.6 Hz, 6H, H-5′ and H-6′); ^13^C NMR (CDCl_3_, 101 MHz): δ 166.85 (C-1), 156.31 (C-3), 116.16 (C-2), 63.97 (C-1′), 35.11 (C-2′), 27.77 (C-4′), 27.39 (C-4), 26.66 (C-3′), 22.52 (C-5′ and C-6′), 20.18 (C-5).

Hexyl 3-methylbut-2-enoate (**3e**; syn. hexyl senecioate): Yield: 80%; RI = 1325 (DB-5MS); IR (ATR, cm^−1^): 2930, 2859, 1717, 1653, 1449, 1377, 1347, 1272, 1225, 1142, 1078, 999, 850; MS (EI), *m*/*z* (%) 184(5), 101(40), 100(71), 85(13), 84(12), 83(100), 82(20), 69(3), 56(8), 55(37), 53(11), 43(25), 41(23), 39(20); ^1^H NMR (CDCl_3_, 400 MHz): δ 5.68 (heptet, *J* = 1.3 Hz, 1H, H-2), 4.08 (triplet, *J* = 6.7 Hz, 2H, H-1′), 2.16 (doublet, *J* = 1.3 Hz, 3H, H-5), 1.89 (doublet, *J* = 1.3 Hz, 3H, H-4), 1.63 (pseudo quintet, *J* = 6.7 Hz, 2H, H-2′), 1.41–1.29 (overlapping peaks, 6H, H-3′–H-5′), 0.89 (pseudo triplet, *J* = 7.0 Hz, 3H, H-6′); ^13^C NMR (CDCl_3_, 101 MHz): δ 166.87 (C-1), 156.26 (C-3), 116.18 (C-2), 63.73 (C-1′), 31.49 (C-4′), 28.73 (C-2′), 27.38 (C-4), 25.70 (C-5′), 22.56 (C-3′), 20.18 (C-5), 14.01 (C-6′).

2-Methylpentyl (*E*)-2-methylbut-2-enoate (**4b**; syn. 2-methylpentyl tiglate): Yield: 64%; RI = 1289 (DB-5MS); IR (ATR, cm^−1^): 2958, 2930, 1708, 1653, 1467, 1380, 1342, 1252, 1134, 1076, 1025, 985; MS (EI), *m*/*z* (%) 101(34), 100(5), 85(8), 84(44), 83(100), 69(15), 57(5), 56(30), 55(70), 53(13), 42(9), 41(27); ^1^H NMR (CDCl_3_, 400 MHz): δ 6.85 (quartet of quartets, *J* = 7.0, 1.4 Hz, 1H, H-3), 4.02 (doublet of doublets, *J* = 10.7, 5.7 Hz, 1H, H_a_-1′), 3.91 (doublet of doublets, *J* = 10.7, 6.7 Hz, 1H, H_b_-1′), 1.84 (pseudo quintet, *J* = 1.4 Hz, 3H, H-5), 1.84 * (overlapped signals, H-2′), 1.79 (doublet of quartets, *J* = 7.0, 1.4 Hz, 3H, H-4), 1.42–1.26 (overlapping peaks, 3H, H_a_-3′ and H-4′), 1.24–1.11 (multiplet, 1H, H_b_-3′), 0.95 (doublet, *J* = 6.7 Hz, 3H, H-6′), 0.91 (pseudo triplet, *J* = 7.2 Hz, 3H, H-5′); ^13^C NMR (CDCl_3_, 101 MHz): δ 168.25 (C-1), 136.79 (C-3), 128.87 (C-2), 69.39 (C-1′), 35.75 (C-3′), 32.44 (C-2′), 19.98 (C-4′), 17.00 (C-6′), 14.32 (C-4), 14.28 (C-5′), 12.05 (C-5). * Center at ~1.84 according to the HSQC spectrum.

3-Methylpentyl (*E*)-2-methylbut-2-enoate (**4c**; syn. 3-methylpentyl tiglate) [32]: Yield: 66%; RI = 1304 (DB-5MS); IR (ATR, cm^−1^): 2960, 2928, 2876, 1709, 1653, 1460, 1380, 1344, 1254, 1134, 1077, 1021, 974; MS (EI), *m*/*z* (%) 101(50), 100(8), 85(11), 84(69), 83(88), 69(50), 57(15), 56(24), 55(100), 54(12), 53(20), 43(23), 41(40); ^1^H NMR (CDCl_3_, 400 MHz): δ 6.85 (quartet of quartets, *J* = 7.0, 1.4 Hz, 1H, H-3), 4.22–4.11 (multiplet, 2H, H-1′), 1.84 (pseudo quintet, *J* = 1.4 Hz, 3H, H-5), 1.79 (doublet of quartets, *J* = 7.0, 1.4 Hz, 3H, H-4), 1.75–1.66 (multiplet, 1H, H_a_-2′), 1.54–1.43 (overlapping peaks, 3H, H_b_-2′ and H-3′), 1.42–1.33 (multiplet, 1H, H_a_-4′), 1.26–1.14 (multiplet, 1H, H_b_-4′), 0.91 (doublet, *J* = 6.7 Hz, 3H, H-6′), 0.88 (pseudo triplet, *J* = 7.4 Hz, 3H, H-5′); ^13^C NMR (CDCl_3_, 101 MHz): δ 168.27 (C-1), 136.82 (C-3), 128.84 (C-2), 63.05 (C-1′), 35.20 (C-2′), 31.57 (C-3′), 29.44 (C-4′), 19.10 (C-6′), 14.32 (C-4), 12.05 (C-5), 11.25 (C-5′).

4-Methylpentyl (*E*)-2-methylbut-2-enoate (**4d**; syn. 4-methylpentyl tiglate) [7]: Yield: 66%; RI = 1298 (DB-5MS); IR (ATR, cm^−1^): 2954, 2928, 2870, 1709, 1653, 1468, 1384, 1367, 1342, 1256, 1134, 1077, 993, 733; MS (EI), *m*/*z* (%) 184(1), 102(5), 101(100), 100(10), 85(10), 84(23), 83(84), 82(8), 69(16), 57(9), 56(30), 55(85), 54(11), 53(18), 43(45), 41(46), 39(32); ^1^H NMR (CDCl_3_, 400 MHz): δ 6.85 (quartet of quartets, *J* = 7.0, 1.4 Hz, 1H, H-3), 4.11 (triplet, *J* = 6.7 Hz, 2H, H-1′), 1.83 (pseudo quintet, *J* = 1.4 Hz, 3H, H-5), 1.79 (doublet of quartets, *J* = 7.0, 1.4 Hz, 3H, H-4), 1.70–1.62 (multiplet, 2H, H-3′), 1.58 (pseudo nonet, *J* = 6.6 Hz, 1H, H-4′), 1.31–1.19 (multiplet, 2H, H-2′), 0.90 (doublet, *J* = 6.6 Hz, 6H, H-5′ and H-6′); ^13^C NMR (CDCl_3_, 101 MHz): δ 168.24 (C-1), 136.80 (C-3), 128.81 (C-2), 64.85 (C-1′), 35.14 (C-2′), 27.76 (C-4′), 26.62 (C-3′), 22.52 (C-5′ and C-6′), 14.32 (C-4), 12.04 (C-5).

Hexyl (*E*)-2-methylbut-2-enoate (**4e**; syn. hexyl tiglate) [7]: Yield: 79%; RI = 1334 (DB-5MS); IR (ATR, cm^−1^): 2955, 2929, 2859, 1709, 1653, 1467, 1381, 1342, 1253, 1135, 1077, 1023, 733; MS (EI), *m*/*z* (%) 184(1), 169(3), 111(3), 102(6), 101(100), 100(16), 84(11), 83(77), 82(9), 69(10), 57(7), 56(21), 55(84), 53(17), 43(27), 41(34), 39(30); ^1^H NMR (CDCl_3_, 400 MHz): δ 6.85 (quartet of quartets, *J* = 7.1, 1.5 Hz, 1H, H-3), 4.12 (triplet, *J* = 6.7 Hz, 2H, H-1′), 1.83 (pseudo quintet, *J* = 1.5 Hz, 3H, H-5), 1.79 (doublet of quartets, *J* = 7.1, 1.5 Hz, 3H, H-4), 1.65 (pseudo quintet, *J* = 6.7 Hz, 3H, H-2′), 1.42–1.27 (overlapping peaks, 6H, H-3′–H-5′), 0.90 (pseudo triplet, *J* = 6.7 Hz, 3H, H-6′); ^13^C NMR (CDCl_3_, 101 MHz): δ 168.27 (C-1), 136.81 (C-3), 128.80 (C-2), 64.60 (C-1′), 31.47 (C-4′), 28.67 (C-2′), 25.71 (C-5′), 22.55 (C-3′), 14.32 (C-4), 14.01 (C-6′), 12.04 (C-5).

3-Methylpentyl 2-methylbutanoate (**5c**): Yield: 80%; RI = 1204 (DB-5MS); IR (ATR, cm^−1^): 2963, 2932, 2877, 1732, 1461, 1380, 1359, 1263, 1239, 1181, 1150, 1083, 1013, 972; MS (EI), *m*/*z* (%) 129(8), 103(33), 102(3), 87(4), 85(61), 84(84), 83(4), 74(6), 69(62), 57(100), 56(33), 55(35), 53(6), 43(41), 42(10), 41(73), 39(22); ^1^H NMR (CDCl_3_, 400 MHz): δ 4.17–4.05 (multiplet, 2H, H-1′), 2.36 (sextet, *J* = 7.0 Hz, 1H, H-2), 1.73–1.61 (overlapping peaks, 2H, H-2′a and H-3a), 1.52–1.40 (overlapping peaks, 3H, H-2′b, H-3′, and H-3b), 1.39–1.31 (multiplet, 1H, H-4′a), 1.24–1.15 (multiplet, 1H, H-4′b), 1.14 (d, *J* = 7.0 Hz, 3H, H-5), 0.90 (t, *J* = 7.4 Hz, 3H, H-4), 0.90 (d, *J* = 6.3 Hz, 3H, H-6′), 0.88 (pseudo triplet, *J* = 7.4 Hz, 3H, H-5′); ^13^C NMR (CDCl_3_, 101 MHz): δ 176.85 (C-1), 62.75 (C-1′), 41.19 (C-2), 35.21 (C-2′), 31.48 (C-3′), 29.43 (C-4′), 26.84 (C-3), 19.05 (C-6′), 16.67 (C-5), 11.65 (C-4), 11.25 (C-5′).

3-Methylpentyl 3-methylbutanoate (**6c**) [33]: Yield: 82%; RI = 1208 (DB-5MS); IR (ATR, cm^−1^): 2959, 2928, 2874, 1736, 1464, 1369, 1294, 1253, 1186, 1169, 1120, 1097, 999; MS (EI), *m*/*z* (%) 157(3), 129(10), 103(31), 87(6), 85(100), 84(94), 83(5), 70(4), 69(71), 57(75), 56(35), 55(38), 53(6), 43(55), 42(21), 41(76), 39(26); ^1^H NMR (CDCl_3_, 400 MHz): δ 4.17–4.05 (multiplet, 2H, H-1′), 2.18 (d, *J* = 7.6 Hz, 2H, H-2), 2.15–2.04 (multiplet, 1H, H-3), 1.71–1.62 (multiplet, 1H, H-2′a), 1.50–1.31 (overlapping peaks, 3H, H-2′b, H-3′, and H-4′a), 1.24–1.13 (multiplet, 1H, H-4′b), 0.96 (d, *J* = 6.5 Hz, 6H, H.4 and H-5), 0.90 (d, *J* = 6.3 Hz, 3H, H-6′), 0.88 (pseudo triplet, *J* = 7.4 Hz, 3H, H-5′); ^13^C NMR (CDCl_3_, 101 MHz): δ 173.27 (C-1), 62.75 (C-1′), 43.57 (C-2), 35.19 (C-2′), 31.43 (C-3′), 29.40 (C-4′), 25.75 (C-3), 22.42 (C-4 and C-5), 19.03 (C-6′), 11.23 (C-5′).

4-Methylhexyl (*Z*)-2-methylbut-2-enoate (**2f**; syn. 4-methylhexyl angelate): RI = 1358 (DB-5MS); MS (EI), *m*/*z* (%) 198(1), 101(23), 100(100), 98(15), 83(95), 82(11), 69(15), 57(72), 56(54), 55(80), 53(10), 43(31), 41(33).

5-Methylhexyl (*Z*)-2-methylbut-2-enoate (**2g**; syn. 5-methylhexyl angelate): Yield: 58%; RI = 1349 (DB-5MS); IR (ATR, cm^−1^): 2954, 2871, 1710, 1655, 1466, 1385, 1365, 1254, 1136, 1078, 734; MS (EI), *m*/*z* (%) 198(5), 101(29), 100(100), 99(5), 85(8), 83(54), 82(15), 69(9), 57(74), 56(20), 55(60), 53(10), 43(34); ^1^H NMR (CDCl_3_, 400 MHz): δ 6.04 (quartet of quartets, *J* = 7.3, 1.5 Hz, 1H, H-3), 4.13 (triplet, *J* = 6.7 Hz, 2H, H-1′), 1.98 (doublet of quartets, *J* = 7.3, 1.5 Hz, 3H, H-4), 1.89 (pseudo quintet, *J* = 1.5 Hz, 3H, H-5), 1.65 (pseudo quintet, *J* = 6.7 Hz, 2H, H-2′), 1.54 (nonet, *J* = 6.6 Hz, 1H, H-5′), 1.41–1.33 (multiplet, 2H, H-3′), 1.27–1.17 (multiplet, 2H, H-4′), 0.88 (doublet, *J* = 6.6 Hz, 6H, H-6′ and H-7′); ^13^C NMR (CDCl_3_, 101 MHz): δ 168.29 (C-1), 137.28 (C-3), 128.14 (C-2), 64.24 (C-1′), 38.53 (C-4′), 28.90 (C-2′), 27.87 (C-5′), 23.85 (C-3′), 22.56 (C-6′ and C-7′), 20.63 (C-4), 15.72 (C-5).

### 3.7. Synthesis of Methallyl Esters

A solution of the appropriate carboxylic acid (0.5 g in 40 mL of dry acetone), anhydrous K_2_CO_3_ (2 equation), 20 µL of triethyl amine, and freshly distilled methallyl chloride (2 equation) was refluxed 2 h and additionally stirred overnight at room temperature. The reaction was quenched by the addition of water (25 mL). Acetone was evaporated and the remaining water layer was extracted with Et_2_O (3 × 40 mL). The organic layers were dried with anhydrous MgSO_4_ and concentrated under reduced pressure to give a crude product that, according to the GC-MS analysis, represented pure esters.

2-Methylprop-2-en-1-yl 2-methylprop-2-enoate (**1h**; syn. methallyl methacrylate) [21]: Yield: 79%; RI = 947 (DB-5MS); MS (EI), *m*/*z* (%) 125(5), 95(6), 70(6), 69(100), 67(3), 55(19), 53(7), 43(6), 41(61), 39(50); ^1^H NMR (CDCl_3_, 400 MHz): δ 6.15 (doublet of quartets, *J* = 1.6, 1.0 Hz, 1H, H_a_-3), 5.59 (pseudo quintet *J* = 1.6 Hz, 1H, H_b_-3), 5.00 (pseudo triplet of quintets, *J* = 1.6, 0.8 Hz, 1H, H_a_-3′), 4.95–4.93 (multiplet, 1H, H_b_-3′), 4.58 (broad singlet, 2H, H-1′), 1.97 (doublet of doublets, *J* = 1.6, 1.0 Hz, 3H, H-4), 1.78 (broad doublet of doublets, *J* = 1.6, 0.8 Hz, 3H, H-4′); ^13^C NMR (CDCl_3_, 101 MHz): δ 167.06 (C-1), 140.02 (C-2′), 136.27 (C-2), 125.61 (C-3), 112.64 (C-3′), 67.84 (C-1′), 19.52 (C-4′), 18.34 (C-4).

2-Methylprop-2-en-1-yl 2-methylbutanoate (**5h**; syn. methallyl 2-methylbutanoate): Yield: 77%; RI = 1015 (DB-5MS); IR (ATR, cm^−1^): 2970, 2937, 2879, 1734, 1660, 1461, 1379, 1299, 1263, 1234, 1178, 1145, 1079, 1029, 1012, 953, 902, 792; MS (EI), *m*/*z* (%) 85(40), 72(5), 58(4), 57(100), 56(7), 55(25), 43(3), 41(25), 39(17); ^1^H NMR (CDCl_3_, 400 MHz): δ 4.99–4.97 (multiplet, 1H, H_a_-3′), 4.93–4.91 (multiplet, 1H, H_b_-3′), 4.51 (broad singlet, 2H, H-1′), 2.42 (pseudo sextet, *J* = 7.0 Hz, 1H, H-2), 1.77–1.76 (multiplet, 3H, H-4′), 1.75–1.65 (multiplet, 1H, H_a_-3), 1.50 (doublet of quartets of doublets, *J* = 13.8, 7.4, 7.0 Hz, 1H, H_b_-3), 1.17 (doublet, *J* = 7.0 Hz, 3H, H-5), 0.92 (triplet, *J* = 7.4 Hz, 3H, H-4); ^13^C NMR (CDCl_3_, 101 MHz): δ 176.37 (C-1), 140.21 (C-2′), 112.70 (C-3′), 67.41 (C-1′), 41.17 (C-2), 26.81 (C-3), 19.51 (C-4′), 16.66 (C-5), 11.65 (C-4).

2-Methylprop-2-en-1-yl 3-methylbutanoate (**6h**; syn. methallyl 3-methylbutanoate): Yield: 80%; RI = 1017 (DB-5MS); IR (ATR, cm^−1^): 2969, 2936, 2880, 1735, 1661, 1462, 1379, 1299, 1264, 1232, 1179, 1145, 1079, 1029, 1012, 953, 903, 792; MS (EI), *m*/*z* (%) 86(5), 85(91), 72(7), 69(7), 59(7), 57(100), 55(38), 53(7), 43(15), 41(32), 39(28); ^1^H NMR (CDCl_3_, 400 MHz): δ 4.99–4.97 (multiplet, 1H, H_a_-3′), 4.93–4.91 (multiplet, 1H, H_b_-3′), 4.50 (broad singlet, 2H, H-1′), 2.24 (doublet, *J* = 7.3 Hz, 2H, H-2), 2.19–2.08 (multiplet, 1H, H-3), 1.76 (doublet of doublets, *J* = 1.6, 0.9 Hz, 3H, H-4′), 0.97 (doublet, *J* = 6.6 Hz, 6H, H-4 and H-5); ^13^C NMR (CDCl_3_, 101 MHz): δ 172.84 (C-1), 140.08 (C-2′), 112.85 (C-3′), 67.47 (C-1′), 43.42 (C-2), 25.72 (C-3), 19.54 (C-4′), 22.43 (C-4 and C-5).

2-Methylprop-2-en-1-yl (*Z*)-2-methylbut-2-enoate (**2h**; syn. methallyl angelate) [20]: Yield: 80%; RI = 1062 (DB-5MS); IR (ATR, cm^−1^): 2928, 1717, 1653, 1456, 1379, 1349, 1255, 1229, 1151, 1084, 1043, 992, 902, 847; MS (EI), *m*/*z* (%) 139(3), 111(3), 84(5), 83(91), 82(13), 81(4), 71(4), 67(3), 56(7), 55(100), 54(10), 53(19), 41(9), 40(5), 39(37); ^1^H NMR (CDCl_3_, 400 MHz): δ 6.09 (quartet of quartets, *J* = 7.3, 1.5 Hz, 1H, H-3), 5.02–4.99 (multiplet, 1H, H_a_-3′), 4.95–4.93 (multiplet, 1H, H_b_-3′), 4.58 (broad singlet, 2H, H-1′), 2.00 (doublet of quartets, *J* = 7.3, 1.5 Hz, 3H, H-4), 1.92 (pseudo quintet, *J* = 1.5 Hz, 3H, H-5), 1.79 (doublet of doublets, *J* = 1.6., 0.8 Hz, 3H, H-4′); ^13^C NMR (CDCl_3_, 101 MHz): δ 167.66 (C-1), 140.14 (C-2′), 138.19 (C-3), 127.77 (C-2), 112.67 (C-3′), 67.39 (C-1′), 20.63 (C-4), 19.62 (C-4′), 15.73 (C-5).

2-Methylprop-2-en-1-yl 3-methylbut-2-enoate (**3h**; syn. methallyl senecioate): Yield: 82%; RI = 1091 (DB-5MS); MS (EI), *m*/*z* (%) 111(2), 84(6), 83(100), 82(4), 55(32), 53(9), 41(5), 40(3), 39(22); IR (ATR, cm^−1^): 2977, 2918, 1720, 1651, 1446, 1378, 1346, 1269, 1222, 1139, 1078, 1003, 972, 902, 850; ^1^H NMR (CDCl_3_, 400 MHz): δ 5.73 (heptet, *J* = 1.3 Hz, 1H, H-2), 4.99–4.97 (multiplet, 1H, H_a_-3′), 4.93–4.91 (multiplet, 1H, H_b_-3′), 4.52 (broad singlet, 2H, H-1′), 2.18 (doublet, *J* = 1.3 Hz, 3H, H-5), 1.91 (doublet, *J* = 1.3 Hz, 3H, H-4), 1.77 (broad singlet, *J* = 1.6., 0.8 Hz, 3H, H-4′); ^13^C NMR (CDCl_3_, 101 MHz): δ 166.33 (C-1), 157.16 (C-3), 140.35 (C-2′), 115.79 (C-2), 112.52 (C-3′), 66.85 (C-1′), 27.45 (C-4), 20.26 (C-5), 19.56 (C-4′).

2-Methylprop-2-en-1-yl (*E*)-2-methylbut-2-enoate (**4h**; syn. methallyl tiglate) [20]: Yield: 84%; RI = 1106 (DB-5MS); MS (EI), *m*/*z* (%) 139(7), 109(5), 93(4), 84(6), 83(100), 67(3), 56(6), 55(88), 54(8), 53(17), 41(8), 40(5), 39(34); IR (ATR, cm^−1^): 2929, 1714, 1653, 1446, 1380, 1261, 1134, 1078, 902, 733; ^1^H NMR (CDCl_3_, 400 MHz): δ 6.91 (quartet of quartets, *J* = 7.1, 1.2 Hz, 1H), 5.00–4.98 (multiplet, 1H, H_a_-3′), 4.94–4.91 (multiplet, 1H, H_b_-3′), 4.56 (broad singlet, 2H, H-1′), 1.86 (pseudo quintet, *J* = 1.2 Hz, 3H, H-5), 1.80 (doublet of quartets, *J* = 7.1, 1.2 Hz, 3H, H-4), 1.79–1.77 (multiplet, 3H, H-4′); ^13^C NMR (CDCl_3_, 101 MHz): δ 167.74 (C-1), 140.32 (C-2′), 137.40 (C-3), 128.55 (C-2), 112.42 (C-3′), 67.63 (C-1′), 19.55 (C-4′), 14.38 (C-4), 12.05 (C-5).

### 3.8. Synthesis of 3-Methylpentyl 2-Methyl-3-(methylthio)propanoate

A solution of dimethyl disulfide (DMDS; 250 µL; 2.81 mmol) and sodium borohydride (NaBH_4_; 100 mg; 2.64 mmol) in dry methanol (10 mL) were stirred at room temperature for 10 min, after which 3-methylpentyl methacrylate (50 mg; 0.29 mmol) was added and the mixture was stirred overnight. Subsequently, 50 mL of saturated NaCl solution was added, and the reaction mixture was extracted with diethyl ether (3 × 50 mL). The combined organic layers were washed with water, dried with anhydrous MgSO_4_, and concentrated under reduced pressure to yield 30 mg of crude product. The obtained crude product was purified by dry-flash chromatography on SiO_2_ using mixtures of the increasing polarity of hexane and Et_2_O as the eluent to give pure 3-methylpentyl 2-methyl-3-(methylthio)propanoate (25 mg).

3-Methylpentyl 2-methyl-3-(methylthio)propanoate (**7c**): Yield: 39%; RI = 1493 (DB-5MS); MS (EI), *m*/*z* (%) 218(22), 135(8), 134(54), 117(26), 90(5), 89(58), 88(38), 85(16), 84(9), 69(15), 61(87), 57(28), 56(11), 45(19), 43(34), 42(16), 41(100), 39(35); IR (ATR, cm^−1^): 2961, 2921, 2876, 1734, 1460, 1378, 1349, 1204, 1162, 1118, 1070, 969, 807; ^1^H NMR (CDCl_3_, 400 MHz): δ 4.19–4.08 (multiplet, 2H, H-1′), 2.82 (doublet of doublets, *J* = 12.9, 7.2 Hz, 1H, H_b_-3), 2.74–2.64 (multiplet, 1H, H-2), 2.55 (doublet of doublets, *J* = 12.9, 6.8 Hz, 1H, H_a_-3), 2.11 (singlet, 3H, H-5), 1.73–1.63 (multiplet, 1H, H_a_-2′), 1.52–1.42 (overlapping peaks, 2H, H-3′ and H_b_-2′), 1.41–1.33 (multiplet, 1H, H_a_-4′), 1.25 (doublet, *J* = 6.9 Hz, 3H, H-4), 1.23–1.14 (multiplet, 1H, H_b_-4′), 0.91 (doublet, *J* = 6.4 Hz, 3H, H-6′), 0.88 (pseudo triplet, *J* = 7.3 Hz, 3H, H-5′); ^13^C NMR (CDCl_3_, 101 MHz): δ 175.29 (C-1), 63.24 (C-1′), 39.85 (C-2), 37.65 (C-3), 35.08 (C-2′), 31.42 (C-3′), 29.37 (C-4′), 19.04 (C-6′), 16.81 (C-4), 16.00 (C-5), 11.25 (C-5′).

### 3.9. Synthesis of 3-Methylpentyl 2-Methyl-3-(methylthio)butanoate and 2-Methylallyl 2-methyl-3-(methylthio)butanoate

The workflow, using a solution of dimethyl disulfide (DMDS; 25 µL; 0.28 mmol) and sodium borohydride (NaBH_4_; 10 mg; 0.26 mmol) in dry methanol (2 mL), is the same as that used for the synthesis of 3-methylpentyl 2-methyl-3-(methylthio)propanoate. The solution was stirred at room temperature for 10 min, after which 3-methylpentyl tiglate or methallyl tiglate (0.03 mmol) was added, and the mixture was stirred overnight. Subsequently, 5 mL of saturated NaCl solution was added, and the reaction mixture was extracted with diethyl ether (3 × 5 mL). The combined organic layers were washed with water, dried with anhydrous MgSO_4_, concentrated under reduced pressure, and directly analyzes by GC–MS.

3-Methylpentyl 2-methyl-3-(methylthio)butanoate (**8c**): RI = 1550 (DB-5MS); MS (EI), *m*/*z* (%) 232(9), 131(7), 102(26), 85(7), 77(5), 75(100), 69(6), 59(6), 55(35), 47(11), 43(50), 41(54),39(17).

2-Methylallyl 2-methyl-3-(methylthio)butanoate (**8h**): RI = 1358 (DB-5MS); MS (EI), *m*/*z* (%) 202(23), 147(16), 131(12), 101(35), 99(5), 87(7), 75(85), 71(5), 56(21), 55(100), 53(21), 45(15), 40(8), 39(25).

### 3.10. Evaluation of Acute Toxicity in the Model of Artemia salina

The method for *Artemia salina* (brine shrimp) cyst hatching used here was previously described by Radulović et al. [26]. The final concentrations of the essential oil and synthesized compounds in artificial seawater (methallyl 2-methylbutanoate (**5h**), methallyl 3-methylbutanoate (**6h**), methallyl angelate (**2h**), 3-methylpentyl methacrylate (**1c**), 4-methylpentyl methacrylate (**1d**), 3-methylpentyl 2-methylbutanoate (**5c**), 3-methylpentyl 3-methylbutanoate (**6c**), 2-methylpentyl angelate (**2b**), 3-methylpentyl angelate (**2c**), 4-methylpentyl angelate (**2d**), hexyl angelate (**2e**), 2-methylpentyl senecioate (**3b**), 3-methylpentyl senecioate (**3c**), 4-methylpentyl senecioate (**3d**), hexyl senecioate (**3e**), 2-methylpentyl tiglate (**4b**), 3-methylpentyl tiglate (**4c**), 4-methylpentyl tiglate (**4d**), and hexyl tiglate (**4e**)) were as follows: 3.9, 7.8, 15.6, 31.3, 62.5, and 125 µg/mL. The final concentration of DMSO was less than 0.5% (*v*/*v*). The tested samples were not aerated, and the test dishes were left at room temperature under constant illumination; brine shrimps (20 nauplii per Petri dish) were not fed during the test. Dead nauplii were counted after 24 and 48 h. Statistical analysis determined a concentration lethal to 50% of nauplii (LC_50_). Sodium dodecyl sulfate (SDS) was used as a positive control. DMSO was inactive under the stated conditions, as demonstrated by a negative control. All the tests were performed in triplicate and repeated twice.

## 4. Conclusions

Using comprehensive GC-MS, preparative EO fractionation, and targeted synthesis/co-injection (25 reference esters with full MS/IR/NMR/RI data), we identified 190 constituents of *A. nobilis* EO. Four of these (methallyl 3-methylbutanoate (**6h**), methallyl senecioate (**3h**), 3-methylpentyl 2-methylbutanoate (**5c**), and 5-methylhexyl angelate (**2g**)) are new natural products. A sublibrary of the prepared natural and structurally related compounds, including the identified constituents of *A. nobilis* EO, was evaluated for acute toxicity using the *Artemia salina* bioassay. Among the tested compounds, only the methacrylates (3-methylpentyl (**1c**) and 4-methylpentyl methacrylate (**1d**)) exhibited low toxicity at the tested concentrations, while the essential oil sample itself and other tested esters were found to be non-toxic to *A. salina* (mortality at the highest tested concentrations was below 10%, comparable to that of the negative control). These findings suggest that the presence of such compounds in *A. nobilis* is unlikely to compromise the safety of botanical preparations derived from this plant. Nonetheless, potential adverse interactions with other constituents within the plant matrix cannot be excluded, and further studies are warranted to assess the toxicity of these compounds in more complex biological contexts.

Additionally, the reactivity of selected methacrylates, tiglates, and angelates as potential Michael acceptors, through their interaction with thiol groups, was evaluated. The discovery of new natural products, particularly methacrylates that exhibit pronounced reactivity as Michael acceptors, emphasizes the chemical complexity of the essential oil and its promising potential for new bioproduct development. These results not only expand the current knowledge of the chemical diversity of *A. nobilis* essential oil but also highlight its relevance for pharmacological exploration.

## Figures and Tables

**Table 1:** Chemical composition of the essential oil and essential oil fractions of *A. nobilis* aerial parts from Serbia.

RI ^a^	Constituent	Sample ^b^								ID ^c^
		A	B	F1	F2	F3	F4	F5	F6	
780	2-Methylpropyl acetate	0.1	tr			tr				MS, RI
782	Methyl 2-methylbutanoate	tr				tr				MS, RI
790	Oct-1-ene	0.1	0.1	tr						MS, RI
796	Hexan-3-ol		tr						tr	MS, RI, CoI
799	Hexan-2-ol		tr						tr	MS, RI, CoI
802	Hexanal	tr	tr			tr				MS, RI, CoI
808	Butyl acetate	tr				tr				MS, RI
835	Methyl angelate	0.1	0.1			0.1	tr			MS, RI, CoI
836	2-Methylbutanoic acid	tr							tr	MS, RI, CoI
837	3-Methylpentan-1-ol	0.1	0.1						0.8	MS, RI, CoI
842	Ethyl 2-methylbutanoate	0.1	0.1			tr	tr			MS, RI
844	Propyl 2-methylpropanoate	0.1	0.1			tr	tr			MS, RI
845	(*Z*)-Hex-3-en-1-ol		tr						tr	MS, RI, CoI
860	Methyl tiglate	tr					tr			MS, RI, CoI
865	Hexan-1-ol	tr	tr						0.1	MS, RI, CoI
871	3-Methylbutyl acetate	0.1	0.1			tr	0.2			MS, RI
874	2-Methylbutyl acetate	0.2	0.1			tr	0.4			MS, RI
877	Propyl methacrylate	0.1	0.1			0.1	tr			MS, CoI
889	Angelic acid	tr							0.1	MS, RI, CoI
890	Non-1-ene	tr	tr	tr						MS, RI
894	Ethyl angelate	0.1	0.1			0.1	tr			MS, RI, CoI
907	2-Methylpropyl 2-methylpropanoate	2.6	2.9			4.2	4.5			MS, RI
915	Prenyl acetate	0.8	0.8				0.9			MS, RI
922	Methallyl isobutanoate	1.0	1.2			1.6	2.8			MS, CoI
924	α-Thujene	tr		tr						MS, RI
926	2-Methylhexan-1-ol	tr	tr						0.1	MS, RI
930	2-Methylpropyl methacrylate **1a**	3.1	3.0			2.4	0.6			MS, CoI
933	α-Pinene	6.1	9.2	7.8	tr					MS, RI
940	Propyl 2-methylbutanoate	0.1	0.1			0.1	tr			MS, RI
944	Propyl 3-methylbutanoate	tr	tr				tr			MS, RI
946	Butyl 2-methylpropanoate	tr				tr				MS, RI
947	Methallyl methacrylate **1h**	1.7	1.4			2.1	0.7			MS, RI, CoI
950	Camphene	0.1	0.6	1.0	tr					MS, RI
955	Thuja-2,4(10)-diene	tr	tr	tr						MS, RI
960	Benzaldehyde	tr					tr	tr		MS, RI, CoI
964	3-Methylbutyl propanoate	tr	tr			tr	tr			MS, RI
965	Heptan-1-ol	tr	tr						tr	MS, RI, CoI
966	2-Methylbutyl propanoate	tr	tr				tr			MS, RI
974	Sabinene	0.1	0.1	tr	tr					MS, RI
975	Oct-1-en-3-ol	tr	tr						0.4	MS, RI, CoI
975	Butyl methacrylate	tr				0.1	tr			MS, RI, CoI
976	3-Methylpentyl acetate	tr	tr			tr	2.8			MS, RI
980	β-Pinene	1.1	1.1	1.2	tr					MS, RI
989	Myrcene	tr	tr	tr	tr					MS, RI
990	Propyl angelate	1.1	1.1			1.8	1.0			MS, RI, CoI
996	Octan-3-ol	tr	tr						tr	MS, RI, CoI
1006	2-Methylpropyl 2-methylbutanoate	1.1	1.1			2.0	2.4			MS, RI
1009	2-Methylpropyl 3-methylbutanoate	0.1	0.1			0.2	0.5			MS, RI
1010	α-Phellandrene	tr	tr	tr	tr					MS, RI
1013	3-Methylbutyl 2-methylpropanoate	0.5	0.4			0.8	1.4			MS, RI
1014	2-Methylbutyl 2-methylpropanoate	2.3	2.2			4.2	6.2			MS, RI
1015	Methallyl 2-methylbutanoate **5h**	0.3	0.4			0.6	0.6			RI, CoI
1017	Methallyl 3-methylbutanoate **6h**	tr	tr			0.1	tr			**NEW**, CoI
1026	*p*-Cymene	0.2	0.2	tr	0.3					MS, RI
1027	Limonene	0.1	0.1	1.0						MS, RI
1029	Propyl tiglate	tr	tr				0.5			MS, CoI
1031	1,8-Cineole	tr	tr					tr		MS, RI
1036	3-Methylbutyl methacrylate	0.8	0.8			1.1	0.4			MS, RI, CoI
1037	Butyl 2-methylbutanoate	tr	tr				tr			MS, RI
1039	2-Methylbutyl methacrylate	1.7	1.4			1.9	0.6			MS, RI, CoI
1041	(*E*)-β-Ocimene	tr		tr	tr					MS, RI
1045	2-Methylpropyl angelate	11.9	13.9			18.7	5.8			MS, RI, CoI
1051	Prenyl isobutyrate	1.2	1.0			1.7	10.3			MS, RI
1056	γ-Terpinene	tr	tr		tr					MS, RI
1062	Methallyl angelate **2h**	8.5	9.3			15.3	5.9			MS, RI, CoI
1068	*cis*-Sabinene hydrate	tr	tr						0.3	MS, RI
1075	3-Methylpentyl propionate	0.2	0.1			0.2	1.8			MS, CoI
1078	Non-1-en-3-ol	tr	tr						0.1	MS, RI
1081	Pentyl methacrylate	tr				tr	tr			MS, CoI
1081	2-Methylpropyl senecioate	tr	tr			tr	tr			MS, CoI
1084	Prenyl methacrylate	0.7	0.7			1.3	1.2			MS, RI, CoI
1086	Butyl angelate	0.6	0.5			0.8	0.3			MS, RI, CoI
1089	2-Methylpropyl tiglate	0.3	0.2			0.2	4.3			MS, RI, CoI
1091	Methallyl senecioate **3h**	tr					tr			**NEW**, CoI
1096	3-Methylbutyl 2-methylbutanoate	0.3	0.2			0.3	0.4			MS, RI
1099	2-Methylbutyl 2-methylbutanoate	0.9	0.7			1.4	2.1			MS, RI
1101	Linalool	tr	tr						1.0	MS, RI
1103	2-Methylbutyl 3-methylbutanoate	0.1	0.1			0.1	tr			MS, RI
1106	Methallyl tiglate **4h**	0.2	0.2			0.3	4.3			MS, CoI
1111	3-Methylpentyl 2-methylpropanoate	1.1	1.0			2.1	3.5			MS, RI
1126	Prenyl 2-methylbutyrate	0.1	tr			0.1	tr			MS, RI
1132	α-Campholenal	0.1	0.1				2.4	1.4		MS, RI
1133	Butyl tiglate	tr				tr	tr			MS, RI, CoI
1136	4-Methylpentyl methacrylate **1d**	tr				tr	tr			MS, CoI
1141	*trans*-Pinocarveol	1.8							59.1	MS, RI
1141	3-Methylpentyl methacrylate **1c**	3.5	1.7			2.6	2.4			MS, RI, CoI
1141	(1R*, 3S*, 5R*)-Sabinol	tr	3.0						4.7	MS, RI
1144	3-Methylbutyl angelate	5.9	5.4			6.4	2.9			MS, CoI
1148	2-Methylbutyl angelate	9.5	9.3			9.8	4.0	tr		MS, RI, CoI
1155	Camphene hydrate	0.2	0.1						3.6	MS, RI
1157	(*Z*)-2-Methylbut-2-en-1-yl angelate *	0.4	0.3			0.4	tr			MS
1158	Isoborneol	tr	tr						0.2	MS, RI
1164	*iso*-Isopulegol	tr	tr						0.1	MS, RI, CoI
1165	Pinocarvone	2.9	2.7				1.0	77.6		MS, RI
1167	Borneol	0.2	0.2						4.5	MS, RI
1169	Unidentified constituent 1 ^d^	0.1				tr	tr			
1177	*cis*-Pinocamphone	0.4	0.3					1.2		MS, RI
1178	Terpinen-4-ol	tr	tr						2.1	MS, RI
1182	3-Methylbutyl senecioate	tr	tr			0.1				MS, CoI
1183	2-Methylbutyl senecioate	tr				tr	tr			MS, CoI
1185	Pentyl angelate	tr				tr	tr			MS, RI, CoI
1188	*p*-Cymen-8-ol	tr	tr						0.2	MS, RI
1190	*trans*-*p*-Mentha-1(7),8-dien-2-ol	tr	tr						0.3	MS, RI
1190	Prenyl angelate	2.8	2.3			4.3	3.5			MS, RI, CoI
1191	3-Methylbutyl tiglate	tr	tr			tr	1.2			MS, RI, CoI
1193	2-Methylbutyl tiglate	tr	tr				1.1			MS, RI, CoI
1194	α-Terpineol	tr	tr						2.3	MS, RI
1199	Myrtenal	0.8	0.7					6.8		MS, RI
1201	Myrtenol		0.3						6.2	MS, RI
1204	3-Methylpentyl 2-methylbutanoate **5c**	0.5	0.4			0.8	1.2			**NEW**, CoI
1205	α-Campholenol	tr	tr						0.5	MS, RI
1205	Decanal		tr				tr			MS, RI, CoI
1208	3-Methylpentyl 3-methylbutanoate **6c**	tr	tr			tr				MS, RI, CoI
1213	Verbenone		tr						0.5	MS, RI
1220	*trans*-Carveol	tr							0.7	MS, RI
1223	β-Cyclocitral		tr					tr		MS, RI
1229	*cis*-*p*-Mentha-1(7),8-dien-2-ol	tr	tr						0.2	MS, RI
1234	Pentyl tiglate	tr				tr				MS, RI, CoI
1235	(*Z*)-Hex-3-en-1-yl crotonate	tr				tr	tr			MS, RI, CoI
1240	Prenyl tiglate	tr	tr				0.7			MS, RI, CoI
1243	2-Hydroxy-2-methylbut-3-en-1-yl angelate	0.3	0.2						4.5	MS, RI
1244	Cumin aldehyde		tr					tr		MS, RI, CoI
1249	4-Methylpentyl angelate **2d**	tr	tr			tr				MS, CoI
1254	Nerol		tr						0.3	MS, RI
1258	3-Methylpentyl angelate **2c**	10.2	9.9		tr	8.9	4.2	tr		MS, CoI
1259	*cis*-Myrtanol	0.2							0.4	MS, RI
1260	2-Phenethyl acetate	tr				tr				MS, RI
1262	*trans*-Myrtanol	tr							0.2	MS, RI
1268	Nonanoic acid		tr						0.1	MS, RI
1279	(*Z*)-Hex-3-en-1-yl angelate	0.2	0.1			0.2	tr			MS, RI, CoI
1280	3-Methylbutyl 3-hydroxy-2-methylenebutanoate	tr							0.4	MS, RI
1282	2-Methylbutyl 3-hydroxy-2-methylenebutanoate *	tr							0.9	MS
1285	Hexyl angelate **2e**	0.1	0.1			tr				MS, RI, CoI
1288	Bornyl acetate	tr					tr	tr		MS, RI
1293	Undecan-2-one	tr						tr	0.1	MS, RI
1298	Benzyl 2-methylpropanoate	tr	tr				2.5	tr		MS, RI
1304	3-Methylpentyl tiglate **4c**	0.1	0.1			0.1				MS, CoI
1305	*trans*-Pinocarvyl acetate	tr	tr				1.8	0.5		MS, RI
1324	(*Z*)-Hex-3-en-1-yl tiglate	tr					tr			MS, RI, CoI
1327	Myrtenyl acetate	tr					tr	tr		MS, RI
1328	Silphiperfol-5-ene	tr	tr	0.2						MS, RI
1329	2-Methylpropyl benzoate	tr				tr	tr			MS, RI
1334	Hexyl tiglate **4e**	tr				tr	tr			MS, RI, CoI
1335	Benzyl methacrylate	tr					tr			MS, CoI
1336	Presilphiperfol-7-ene		tr	tr						MS, RI
1348	Silphinene	tr	tr	1.1						MS, RI
1349	5-Methylhexyl angelate **2g**	tr				tr	tr			**NEW**, CoI
1363	Neryl acetate	0.1	0.1				2.9	5.4		MS, RI
1371	Cyclosativene	tr	tr	1.0						MS, RI
1379	α-Copaene	0.1	tr	2.3						MS, RI
1383	Geranyl acetate	tr					tr	1.2		MS, RI
1385	Heptyl angelate	tr				tr	tr			MS, CoI
1385	Modheph-2-ene	tr	tr	tr						MS, RI
1386	Unidentified constituent 2 ^e^	tr	tr						0.9	
1387	Benzyl 2-methylbutanoate	tr					tr			MS, RI
1389	Benzyl 3-methylbutanoate	tr					tr	0.9		MS, RI
1394	β-Elemene	tr		0.3						MS, RI
1396	2-Phenylethyl 2-methylpropanoate	tr	tr				tr	1.2		MS, RI
1401	Unidentified constituent 3 ^f^	tr	tr						0.1	
1407	*iso*-Italicene	tr	tr	0.8						MS, RI
1417	*cis*-α-Bergamotene	tr	tr	0.4						MS, RI
1423	(*E*)-Caryophyllene	0.2	0.2	5.8						MS, RI
1432	β-Copaene	tr		tr						MS, RI
1437	α-*trans*-Bergamotene	tr	tr	tr						MS, RI
1443	Benzyl angelate	0.1	tr				tr			MS, RI
1454	Neryl propanoate	tr	tr				tr			MS, RI
1457	(*E*)-β-Farnesene	0.1	0.1	0.2	tr					MS, RI
1463	2-Methyltetradecane	tr		tr						MS, RI
1478	β-Selinene	tr	tr	3.3	tr					MS, RI
1482	γ-Curcumene	0.3	0.2	tr	32.4	tr				MS, RI
1486	Germacrene D	3.2	2.7	43.8	tr					MS, RI
1492	Eudesma-3,5,11-triene	0.4	0.3	13.8	tr					MS, RI
1494	Benzyl tiglate	tr					tr			MS, RI
1500	Bicyclogermacrene	0.3	0.3	6.1	34.4					MS, RI
1503	α-Muurolene	tr		1.4						MS, RI
1509	(*E*,*E*)-alpha-Farnesene	0.4	0.3	1.4	32.3	tr				MS, RI
1518	γ-Cadinene	tr		1.1						MS, RI
1526	δ-Cadinene	0.1	0.1	3.8						MS, RI
1540	2-Phenylethyl angelate	tr					tr			MS, RI
1541	α-Cadinene	tr		0.3						MS, RI
1548	3-Methylpentyl benzoate	tr					tr			MS, CoI
1600	Hexadecane	tr		tr						MS, RI, CoI
1838	Neophytadiene (isomer 1)	tr		tr						MS, RI
1845	Hexahydrofarnesyl acetone	tr						1.9		MS, RI
1900	Nonadecane	tr		tr						MS, RI, CoI
1960	Hexadecanoic acid		tr						0.2	MS, RI
2000	Eicosane		tr	tr						MS, RI, CoI
2100	Heneicosane	tr	tr	tr						MS, RI, CoI
2112	(*E*)-Phytol	tr							2.1	MS, RI
2300	Tricosane	tr	tr	tr						MS, RI, CoI
2400	Tetracosane		tr	tr						MS, RI, CoI
2500	Pentacosane		tr	tr						MS, RI, CoI
2700	Heptacosane	tr	tr	tr						MS, RI, CoI
2900	Nonacosane	tr	tr	tr						MS, RI, CoI
	Total identified [%]	97.6	98.5	98.1	99.4	99.6	98.2	98.1	98.3	

^a^ RI = retention indices relative to a homologous series of n-alkanes (C_7_–C_29_) on a DB-5MS column. ^b^ The essential oil samples of *A. nobilis* aerial parts; samples F1–F6 represent SiO_2_ chromatographic fractions obtained from the pooled essential oils A and B; tr = trace amounts (<0.05%). ^c^ ID = identification method; MS = constituent identified by mass-spectra comparison with those listed in the Wiley 11, NIST17, MassFinder 2.3 and a homemade mass spectral library, RI = constituent identified by retention index matching with literature data, CoI = constituent identity confirmed by GC co-injection of an authentic sample; NEW = new natural product and new compound in general. ^d^ Unidentified constituent 1 (Figure S79): MS (EI), *m*/*z* (%) 139(3), 123(4), 109(6), 101(11), 100(5), 85(8), 84(6), 83(100), 82(4), 69(48), 68(20), 67(27), 57(3), 56(4), 55(70), 54(8), 53(25), 51(7), 50(3), 43(6), 41(47). ^e^ Unidentified constituent 2 (Figure S80): MS (EI), *m*/*z* (%) 185(11), 117(4), 116(10), 115(6), 101(23), 100(4), 99(51), 98(30), 97(12), 86(7), 85(80), 84(23), 83(29), 82(8), 81(25), 73(7), 72(6), 71(20), 70(12), 69(32), 67(5), 57(31), 56(24), 55(67), 54(18), 53(28), 51(5), 45(25), 43(100), 42(12), 41(67). ^f^ Unidentified constituent 3 (Figure S81): MS (EI), *m*/*z* (%) 164(3), 155(6), 122(3), 110(4), 100(8), 99(22), 98(4), 84(7), 83(70), 82(38), 81(4), 79(3), 71(16), 56(5), 55(100), 54(16), 53(21), 51(4), 45(3), 43(46), 41(39). * Tentatively identified essential oil constituents by analysis of mass fragmentation and prediction of retention index (Figure S78).

## Data Availability

Data are contained within the article and Supplementary Materials.

## Supplementary Materials

The following supporting information can be downloaded at: https://www.mdpi.com/article/10.3390/molecules31020256/s1, Figure S1. Mass spectrum of isobutyl methacrylate (**1a**; RI = 930 (DB-5MS column)). Figure S2. ^1^H NMR spectrum of isobutyl methacrylate in CDCl_3_ (400 MHz). Figure S3. ^13^C NMR spectrum of isobutyl methacrylate in CDCl_3_ (101 MHz). Figure S4. Mass spectrum of 3-methylpentyl methacrylate (**1c**; RI = 1141 (DB-5MS column)). Figure S5. ^1^H NMR spectrum of 3-methylpentyl methacrylate in CDCl_3_ (400 MHz). Figure S6. ^13^C NMR spectrum of 3-methylpentyl methacrylate in CDCl_3_ (101 MHz). Figure S7. Mass spectrum of 4-methylpentyl methacrylate (**1d**; RI = 1136 (DB-5MS column)). Figure S8. ^1^H NMR spectrum of 4-methylpentyl methacrylate in CDCl_3_ (400 MHz). Figure S9. ^13^C NMR spectrum of 4-methylpentyl methacrylate in CDCl_3_ (101 MHz). Figure S10. Mass spectrum of 2-methylpentyl angelate (**2b**; RI = 1240 (DB-5MS column)). Figure S11. ^1^H NMR spectrum of 2-methylpentyl angelate in CDCl_3_ (400 MHz). Figure S12. ^13^C NMR spectrum of 2-methylpentyl angelate in CDCl_3_ (101 MHz). Figure S13. Mass spectrum of 3-methylpentyl angelate (**2c**; RI = 1258 (DB-5MS column)). Figure S14. ^1^H NMR spectrum of 3-methylpentyl angelate in CDCl_3_ (400 MHz). Figure S15. ^13^C NMR spectrum of 3-methylpentyl angelate in CDCl_3_ (101 MHz). Figure S16. Mass spectrum of 4-methylpentyl angelate (**2d**; RI = 1249 (DB-5MS column)). Figure S17. ^1^H NMR spectrum of 4-methylpentyl angelate in CDCl_3_ (400 MHz). Figure S18. ^13^C NMR spectrum of 4-methylpentyl angelate in CDCl_3_ (101 MHz). Figure S19. Mass spectrum of hexyl angelate (**2e**; RI = 1285 (DB-5MS column)). Figure S20. ^1^H NMR spectrum of hexyl angelate in CDCl_3_ (400 MHz). Figure S21. ^13^C NMR spectrum of hexyl angelate in CDCl_3_ (101 MHz). Figure S22. Mass spectrum of 2-methylpentyl senecioate (**3b**; RI = 1280 (DB-5MS column)). Figure S23. ^1^H NMR spectrum of 2-methylpentyl senecioate in CDCl_3_ (400 MHz). Figure S24. ^13^C NMR spectrum of 2-methylpentyl senecioate in CDCl_3_ (101 MHz). Figure S25. Mass spectrum of 3-methylpentyl senecioate (**3c**; RI = 1295 (DB-5MS column)). Figure S26. ^1^H NMR spectrum of 3-methylpentyl senecioate in CDCl_3_ (400 MHz). Figure S27. ^13^C NMR spectrum of 3-methylpentyl senecioate in CDCl_3_ (101 MHz). Figure S28. Mass spectrum of 4-methylpentyl senecioate (**3d**; RI = 1289 (DB-5MS column)). Figure S29. ^1^H NMR spectrum of 4-methylpentyl senecioate in CDCl_3_ (400 MHz). Figure S30. ^13^C NMR spectrum of 4-methylpentyl senecioate in CDCl_3_ (101 MHz). Figure S31. Mass spectrum of hexyl senecioate (**3e**; RI = 1325 (DB-5MS column)). Figure S32. ^1^H NMR spectrum of hexyl senecioate in CDCl_3_ (400 MHz). Figure S33. ^13^C NMR spectrum of hexyl senecioate in CDCl_3_ (101 MHz). Figure S34. Mass spectrum of 2-methylpentyl tiglate (**4b**; RI = 1289 (DB-5MS column)). Figure S35. ^1^H NMR spectrum of 2-methylpentyl tiglate in CDCl_3_ (400 MHz). Figure S36. ^13^C NMR spectrum of 2-methylpentyl tiglate in CDCl_3_ (101 MHz). Figure S37. Mass spectrum of 3-methylpentyl tiglate (**4c**; RI = 1304 (DB-5MS column)). Figure S38. ^1^H NMR spectrum of 3-methylpentyl tiglate in CDCl_3_ (400 MHz). Figure S39. ^13^C NMR spectrum of 3-methylpentyl tiglate in CDCl_3_ (101 MHz). Figure S40. Mass spectrum of 4-methylpentyl tiglate (**4d**; RI = 1298 (DB-5MS column)). Figure S41. ^1^H NMR spectrum of 4-methylpentyl tiglate in CDCl_3_ (400 MHz). Figure S42. ^13^C NMR spectrum of 4-methylpentyl tiglate in CDCl_3_ (101 MHz). Figure S43. Mass spectrum of hexyl tiglate (**4e**; RI = 1334 (DB-5MS column)). Figure S44. ^1^H NMR spectrum of hexyl tiglate in CDCl_3_ (400 MHz). Figure S45. ^13^C NMR spectrum of hexyl tiglate in CDCl_3_ (101 MHz). Figure S46. Mass spectrum of 3-methylpentyl 2-methylbutanoate (**5c**; RI = 1204 (DB-5MS column)). Figure S47. ^1^H NMR spectrum of 3-methylpentyl 2-methylbutanoate in CDCl_3_ (400 MHz). Figure S48a. DEPT-90, DEPT-135, and ^13^C NMR spectra (from top to bottom) of 3-methylpentyl 2-methylbutanoate in CDCl_3_ (101 MHz). Figure S48b. HSQC spectrum of 3-methylpentyl 2-methylbutanoate in CDCl_3_ (101 MHz). Figure S48c. HMBC spectrum of 3-methylpentyl 2-methylbutanoate in CDCl_3_ (101 MHz). Figure S49. Mass spectrum of 3-methylpentyl 3-methylbutanoate (**6c**; RI = 1208 (DB-5MS column)). Figure S50. ^1^H NMR spectrum of 3-methylpentyl 3-methylbutanoate in CDCl_3_ (400 MHz). Figure S51. ^13^C NMR spectrum of 3-methylpentyl 3-methylbutanoate in CDCl_3_ (101 MHz). Figure S52. Mass spectrum of 4-methylhexyl angelate (**2f**; RI = 1358 (DB-5MS column)). Figure S53. Mass spectrum of 5-methylhexyl angelate (**2g**; RI = 1349 (DB-5MS column)). Figure S54. ^1^H NMR spectrum of 5-methylhexyl angelate in CDCl_3_ (400 MHz). Figure S55a. ^13^C NMR spectrum of 5-methylhexyl angelate in CDCl_3_ (101 MHz). Figure S55b. HSQC spectrum of 5-methylhexyl angelate in CDCl_3_ (101 MHz). Figure S56. Mass spectrum of methallyl methacrylate (**1h**; RI = 947 (DB-5MS column)). Figure S57. ^1^H NMR spectrum of methallyl methacrylate in CDCl_3_ (400 MHz). Figure S58. ^13^C NMR spectrum of methallyl methacrylate in CDCl_3_ (101 MHz). Figure S59. Mass spectrum of methallyl 2-methylbutanoate (**5h**; RI = 1015 (DB-5MS column)). Figure S60. ^1^H NMR spectrum of methallyl 2-methylbutanoate in CDCl_3_ (400 MHz). Figure S61. ^13^C NMR spectrum of methallyl 2-methylbutanoate in CDCl_3_ (101 MHz). Figure S62. Mass spectrum of methallyl 3-methylbutanoate (**6h**; RI = 1017 (DB-5MS column)). Figure S63. ^1^H NMR spectrum of methallyl 3-methylbutanoate in CDCl_3_ (400 MHz). Figure S64a. DEPT-90, DEPT-135, and ^13^C NMR spectra (from top to bottom) of methallyl 3-methylbutanoate in CDCl_3_ (101 MHz). Figure S64b. HSQC spectrum of methallyl 3-methylbutanoate in CDCl_3_ (101 MHz). Figure S64c. HMBC spectrum of methallyl 3-methylbutanoate in CDCl_3_ (101 MHz). Figure S65. Mass spectrum of methallyl angelate (**2h**; RI = 1062 (DB-5MS column)). Figure S66. ^1^H NMR spectrum of methallyl angelate in CDCl_3_ (400 MHz). Figure S67. ^13^C NMR spectrum of methallyl angelate in CDCl_3_ (101 MHz). Figure S68. Mass spectrum of methallyl senecioate (**3h**; RI = 1091 (DB-5MS column)). Figure S69. ^1^H NMR spectrum of methallyl senecioate in CDCl_3_ (400 MHz). Figure S70a. DEPT-90, DEPT-135, and ^13^C NMR spectra (from top to bottom) of methallyl senecioate in CDCl_3_ (101 MHz). Figure S70b. HSQC spectrum of methallyl senecioate in CDCl_3_ (101 MHz). Figure S70c. HMBC spectrum of methallyl senecioate in CDCl_3_ (101 MHz). Figure S71. Mass spectrum of methallyl tiglate (**4h**; RI = 1106 (DB-5MS column)). Figure S72. ^1^H NMR spectrum of methallyl tiglate in CDCl_3_ (400 MHz). Figure S73. ^13^C NMR spectrum of methallyl tiglate in CDCl_3_ (101 MHz). Figure S74. Mass spectrum of 3-methylpentyl 2-methyl-3-(methylthio)propanoate (**7c**; RI = 1493 (DB-5MS column)). Figure S75. Mass spectrum of 3-methylpentyl 2-methyl-3-(methylthio)butanoate (**8c**; RI = 1550 (DB-5MS column)). Figure S76. Mass spectrum of 2-methylallyl 2-methyl-3-(methylthio)butanoate (**8h**; RI = 1358 (DB-5MS column)). Figure S77. (A) Extracted partial ion chromatograms (*m*/*z* 55, 83, 100, and 101) highlighting diagnostic fragments for the identification of ester series, and (B) representative total ion chromatogram (TIC) of the ester-enriched essential oil fraction. Figure S78. Mass spectrum of tentatively identified (*Z*)-2-methylbut-2-en-1-yl angelate (RI = 1157 (DB-5MS column)). Figure S79. Mass spectrum of unidentified constituent 1 (RI = 1169 (DB-5MS column)). Figure S80. Mass spectrum of unidentified constituent 2 (RI = 1386 (DB-5MS column)). Figure S81. Mass spectrum of unidentified constituent 3 (RI = 1401 (DB-5MS column)). Figure S82. (EI)MS fragmentation patterns of selected rare natural products (methallyl methacrylate (**1h**) and methallyl tiglate (**4h**)) and newly identified natural products (methallyl 3-methylbutanoate (**6h**), methallyl senecioate (**3h**), 3-methylpentyl 2-methylbutanoate (**5c**), and 5-methylhexyl angelate (**2g**)).
