# Supplementary material for: Unlocking the Secrets of Roman Chamomile (Anthemis nobilis L.) Essential Oil: Structural Elucidation and Acute Toxicity of New Esters
Source: Molecules. 2026 Jan 12;31(2):256. doi: 10.3390/molecules31020256 (PMC12843988; doi:10.3390/molecules31020256)
Supplement: Supplementary file 1 [file molecules-31-00256-s001.zip › molecules-4048807-supplementary.pdf]

SUPPLEMENTARY MATERIAL

FOR

Unlocking the Secrets of Roman Chamomile (*Anthemis nobilis* L.) Essential Oil: Structural Elucidation and Acute Toxicity of New Esters

Niko S. Radulović [1,*] and Marko Z. Mladenović [1,2,*]

[1] Department of Chemistry, Faculty of Sciences and Mathematics, University of Niš, Višegradska 33, 18000 Niš, Serbia
[2] Department of Sciences and Mathematics, State University of Novi Pazar, Vuka Karadžića 9, 36300 Novi Pazar, Serbia
* Correspondence: nikoradulovic@yahoo.com (N.R.); markohem87@gmail.com (M.M.); Tel.: +381-18-533-015 (N.R. & M.M.); Fax: +381-18-533-014 (N.R. & M.M.)

Table of Contents

Figure S1. Mass spectrum of isobutyl methacrylate (**1a**; RI = 930 (DB-5MS column))
Figure S2. ^{1}H NMR spectrum of isobutyl methacrylate in $CDCl_3$ (400 MHz)
Figure S3. ^{13}C NMR spectrum of isobutyl methacrylate in $CDCl_3$ (101 MHz)
Figure S4. Mass spectrum of 3-methylpentyl methacrylate (**1c**; RI = 1141 (DB-5MS column))
Figure S5. ^{1}H NMR spectrum of 3-methylpentyl methacrylate in $CDCl_3$ (400 MHz)
Figure S6. ^{13}C NMR spectrum of 3-methylpentyl methacrylate in $CDCl_3$ (101 MHz)
Figure S7. Mass spectrum of 4-methylpentyl methacrylate (**1d**; RI = 1136 (DB-5MS column))
Figure S8. ^{1}H NMR spectrum of 4-methylpentyl methacrylate in $CDCl_3$ (400 MHz)
Figure S9. ^{13}C NMR spectrum of 4-methylpentyl methacrylate in $CDCl_3$ (101 MHz)
Figure S10. Mass spectrum of 2-methylpentyl angelate (**2b**; RI = 1240 (DB-5MS column))
Figure S11. ^{1}H NMR spectrum of 2-methylpentyl angelate in $CDCl_3$ (400 MHz)
Figure S12. ^{13}C NMR spectrum of 2-methylpentyl angelate in $CDCl_3$ (101 MHz)
Figure S13. Mass spectrum of 3-methylpentyl angelate (**2c**; RI = 1258 (DB-5MS column))
Figure S14. ^{1}H NMR spectrum of 3-methylpentyl angelate in $CDCl_3$ (400 MHz)
Figure S15. ^{13}C NMR spectrum of 3-methylpentyl angelate in $CDCl_3$ (101 MHz)
Figure S16. Mass spectrum of 4-methylpentyl angelate (**2d**; RI = 1249 (DB-5MS column))
Figure S17. ^{1}H NMR spectrum of 4-methylpentyl angelate in $CDCl_3$ (400 MHz)
Figure S18. ^{13}C NMR spectrum of 4-methylpentyl angelate in $CDCl_3$ (101 MHz)
Figure S19. Mass spectrum of hexyl angelate (**2e**; RI = 1285 (DB-5MS column))
Figure S20. ^{1}H NMR spectrum of hexyl angelate in $CDCl_3$ (400 MHz)
Figure S21. ^{13}C NMR spectrum of hexyl angelate in $CDCl_3$ (101 MHz)
Figure S22. Mass spectrum of 2-methylpentyl senecioate (**3b**; RI = 1280 (DB-5MS column))
Figure S23. ^{1}H NMR spectrum of 2-methylpentyl senecioate in $CDCl_3$ (400 MHz)
Figure S24. ^{13}C NMR spectrum of 2-methylpentyl senecioate in $CDCl_3$ (101 MHz)

Figure S25. Mass spectrum of 3-methylpentyl senecioate (**3c**; RI = 1295 (DB-5MS column))
Figure S26. ^{1}H NMR spectrum of 3-methylpentyl senecioate in $CDCl_3$ (400 MHz)
Figure S27. ^{13}C NMR spectrum of 3-methylpentyl senecioate in $CDCl_3$ (101 MHz)
Figure S28. Mass spectrum of 4-methylpentyl senecioate (**3d**; RI = 1289 (DB-5MS column))
Figure S29. ^{1}H NMR spectrum of 4-methylpentyl senecioate in $CDCl_3$ (400 MHz)
Figure S30. ^{13}C NMR spectrum of 4-methylpentyl senecioate in $CDCl_3$ (101 MHz)
Figure S31. Mass spectrum of hexyl senecioate (**3e**; RI = 1325 (DB-5MS column))
Figure S32. ^{1}H NMR spectrum of hexyl senecioate in $CDCl_3$ (400 MHz)
Figure S33. ^{13}C NMR spectrum of hexyl senecioate in $CDCl_3$ (101 MHz)
Figure S34. Mass spectrum of 2-methylpentyl tiglate (**4b**; RI = 1289 (DB-5MS column))
Figure S35. ^{1}H NMR spectrum of 2-methylpentyl tiglate in $CDCl_3$ (400 MHz)
Figure S36. ^{13}C NMR spectrum of 2-methylpentyl tiglate in $CDCl_3$ (101 MHz)
Figure S37. Mass spectrum of 3-methylpentyl tiglate (**4c**; RI = 1304 (DB-5MS column))
Figure S38. ^{1}H NMR spectrum of 3-methylpentyl tiglate in $CDCl_3$ (400 MHz)
Figure S39. ^{13}C NMR spectrum of 3-methylpentyl tiglate in $CDCl_3$ (101 MHz)
Figure S40. Mass spectrum of 4-methylpentyl tiglate (**4d**; RI = 1298 (DB-5MS column))
Figure S41. ^{1}H NMR spectrum of 4-methylpentyl tiglate in $CDCl_3$ (400 MHz)
Figure S42. ^{13}C NMR spectrum of 4-methylpentyl tiglate in $CDCl_3$ (101 MHz)
Figure S43. Mass spectrum of hexyl tiglate (**4e**; RI = 1334 (DB-5MS column))
Figure S44. ^{1}H NMR spectrum of hexyl tiglate in $CDCl_3$ (400 MHz)
Figure S45. ^{13}C NMR spectrum of hexyl tiglate in $CDCl_3$ (101 MHz)
Figure S46. Mass spectrum of 3-methylpentyl 2-methylbutanoate (**5c**; RI = 1204 (DB-5MS column))
Figure S47. ^{1}H NMR spectrum of 3-methylpentyl 2-methylbutanoate in $CDCl_3$ (400 MHz)
Figure S48a. DEPT-90, DEPT-135, and ^{13}C NMR spectra (from top to bottom) of 3-methylpentyl 2-methylbutanoate in $CDCl_3$ (101 MHz)
Figure S48b. HSQC spectrum of 3-methylpentyl 2-methylbutanoate in $CDCl_3$ (101 MHz)
Figure S48c. HMBC spectrum of 3-methylpentyl 2-methylbutanoate in $CDCl_3$ (101 MHz)
Figure S49. Mass spectrum of 3-methylpentyl 3-methylbutanoate (**6c**; RI = 1208 (DB-5MS column))
Figure S50. ^{1}H NMR spectrum of 3-methylpentyl 3-methylbutanoate in $CDCl_3$ (400 MHz)
Figure S51. ^{13}C NMR spectrum of 3-methylpentyl 3-methylbutanoate in $CDCl_3$ (101 MHz)
Figure S52. Mass spectrum of 4-methylhexyl angelate (**2f**; RI = 1358 (DB-5MS column))
Figure S53. Mass spectrum of 5-methylhexyl angelate (**2g**; RI = 1349 (DB-5MS column))
Figure S54. ^{1}H NMR spectrum of 5-methylhexyl angelate in $CDCl_3$ (400 MHz)
Figure S55a. ^{13}C NMR spectrum of 5-methylhexyl angelate in $CDCl_3$ (101 MHz)
Figure S55b. HSQC spectrum of 5-methylhexyl angelate in $CDCl_3$ (101 MHz)
Figure S56. Mass spectrum of methallyl methacrylate (**1h**; RI = 947 (DB-5MS column))
Figure S57. ^{1}H NMR spectrum of methallyl methacrylate in $CDCl_3$ (400 MHz)

Figure S58. ^{13}C NMR spectrum of methallyl methacrylate in $CDCl_3$ (101 MHz)
Figure S59. Mass spectrum of methallyl 2-methylbutanoate (**5h**; RI = 1015 (DB-5MS column))
Figure S60. ^{1}H NMR spectrum of methallyl 2-methylbutanoate in $CDCl_3$ (400 MHz)
Figure S61. ^{13}C NMR spectrum of methallyl 2-methylbutanoate in $CDCl_3$ (101 MHz)
Figure S62. Mass spectrum of methallyl 3-methylbutanoate (**6h**; RI = 1017 (DB-5MS column))
Figure S63. ^{1}H NMR spectrum of methallyl 3-methylbutanoate in $CDCl_3$ (400 MHz)
Figure S64a. DEPT-90, DEPT-135, and ^{13}C NMR spectra (from top to bottom) of methallyl 3-methylbutanoate in $CDCl_3$ (101 MHz)
Figure S64b. HSQC spectrum of methallyl 3-methylbutanoate in $CDCl_3$ (101 MHz)
Figure S64c. HMBC spectrum of methallyl 3-methylbutanoate in $CDCl_3$ (101 MHz)
Figure S65. Mass spectrum of methallyl angelate (**2h**; RI = 1062 (DB-5MS column))
Figure S66. ^{1}H NMR spectrum of methallyl angelate in $CDCl_3$ (400 MHz)
Figure S67. ^{13}C NMR spectrum of methallyl angelate in $CDCl_3$ (101 MHz)
Figure S68. Mass spectrum of methallyl senecioate (**3h**; RI = 1091 (DB-5MS column))
Figure S69. ^{1}H NMR spectrum of methallyl senecioate in $CDCl_3$ (400 MHz)
Figure S70a. DEPT-90, DEPT-135, and ^{13}C NMR spectra (from top to bottom) of methallyl senecioate in $CDCl_3$ (101 MHz)
Figure S70b. HSQC spectrum of methallyl senecioate in $CDCl_3$ (101 MHz)
Figure S70c. HMBC spectrum of methallyl senecioate in $CDCl_3$ (101 MHz)
Figure S71. Mass spectrum of methallyl tiglate (**4h**; RI = 1106 (DB-5MS column))
Figure S72. ^{1}H NMR spectrum of methallyl tiglate in $CDCl_3$ (400 MHz)
Figure S73. ^{13}C NMR spectrum of methallyl tiglate in $CDCl_3$ (101 MHz)
Figure S74. Mass spectrum of 3-methylpentyl 2-methyl-3-(methylthio)propanoate (**7c**; RI = 1493 (DB-5MS column))
Figure S75. Mass spectrum of 3-methylpentyl 2-methyl-3-(methylthio)butanoate (**8c**; RI = 1550 (DB-5MS column))
Figure S76. Mass spectrum of 2-methylallyl 2-methyl-3-(methylthio)butanoate (**8h**; RI = 1358 (DB-5MS column))
Figure S77. (A) Extracted partial ion chromatograms (*m/z* 55, 83, 100, and 101) highlighting diagnostic fragments for the identification of ester series, and (B) representative total ion chromatogram (TIC) of the ester-enriched essential oil fraction
Figure S78. Mass spectrum of tentatively identified (*Z*)-2-methylbut-2-en-1-yl angelate (RI = 1157 (DB-5MS column))
Figure S79. Mass spectrum of unidentified constituent 1 (RI = 1169 (DB-5MS column))
Figure S80. Mass spectrum of unidentified constituent 2 (RI = 1386 (DB-5MS column))
Figure S81. Mass spectrum of unidentified constituent 3 (RI = 1401 (DB-5MS column))
Figure S82. (EI)MS fragmentation patterns of selected rare natural products (methallyl methacrylate (**1h**) and methallyl tiglate (**4h**)) and newly identified natural products (methallyl 3-methylbutanoate (**6h**), methallyl senecioate (**3h**), 3-methylpentyl 2-methylbutanoate (**5c**), and 5-methylhexyl angelate (**2g**))

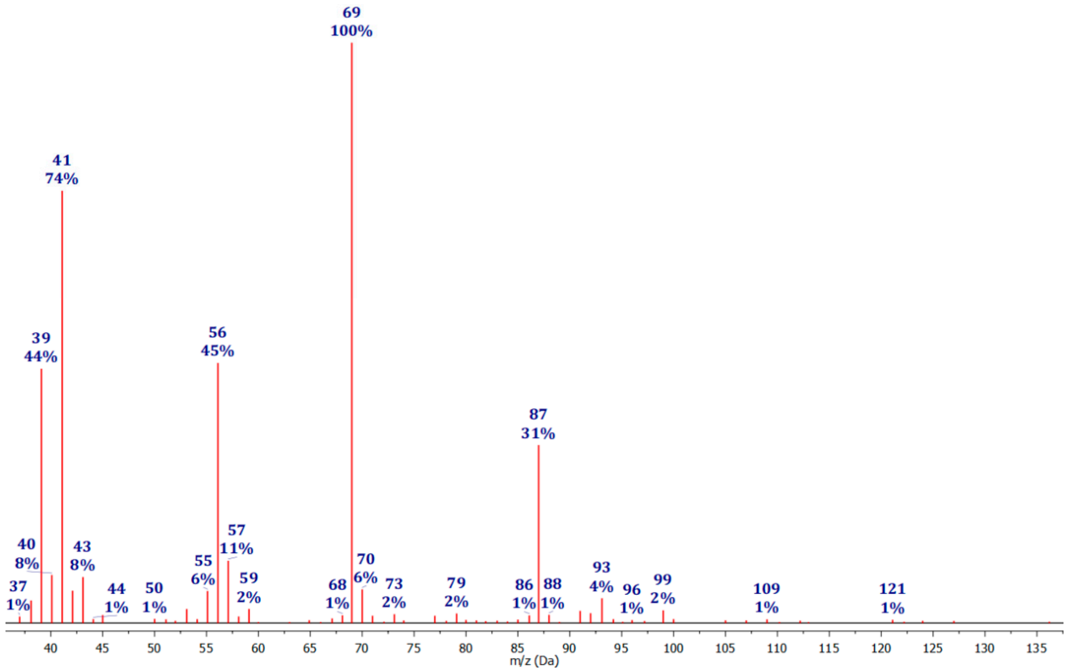

Figure S1. Mass spectrum of isobutyl methacrylate (**1a**; RI = 930 (DB-5MS column))

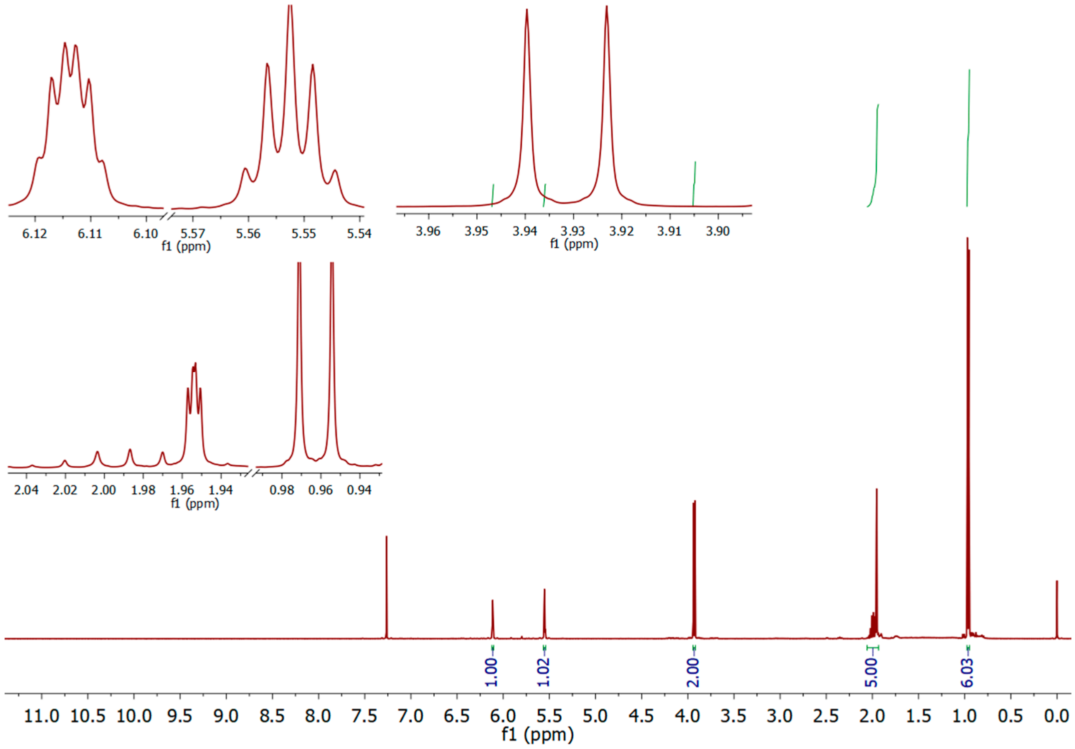

Figure S2. ¹H NMR spectrum of isobutyl methacrylate in $CDCl_3$ (400 MHz)

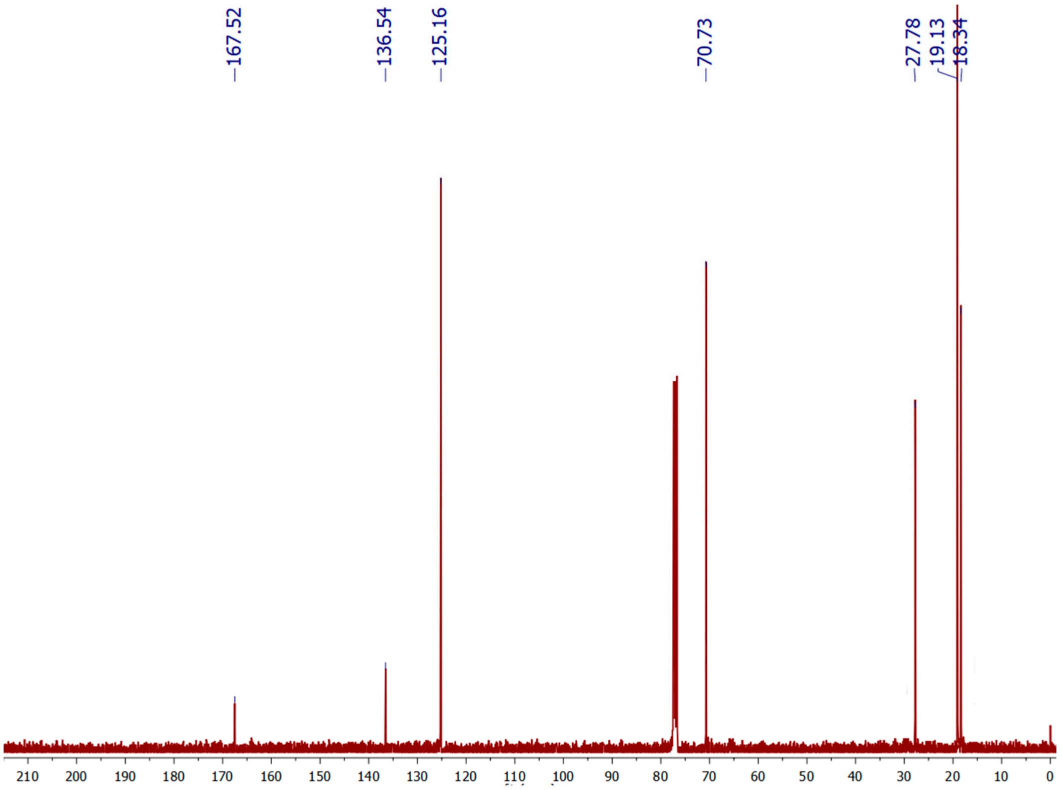

Figure S3. ^{13}C NMR spectrum of isobutyl methacrylate in $CDCl_3$ (101 MHz)

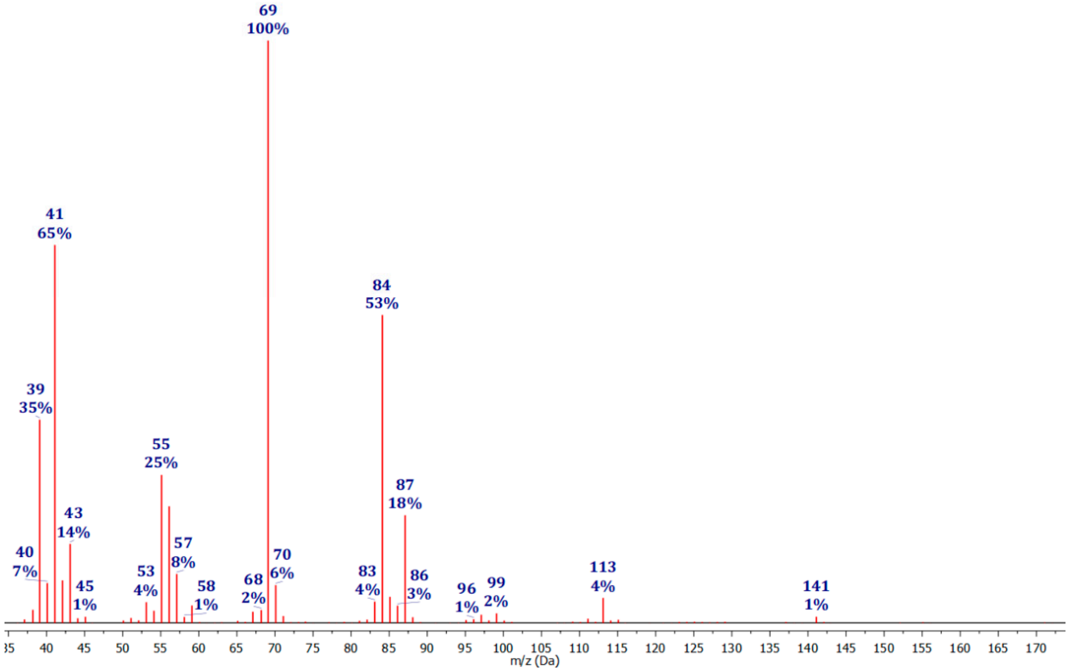

Figure S4. Mass spectrum of 3-methylpentyl methacrylate (**1c**; RI = 1141 (DB-5MS column))

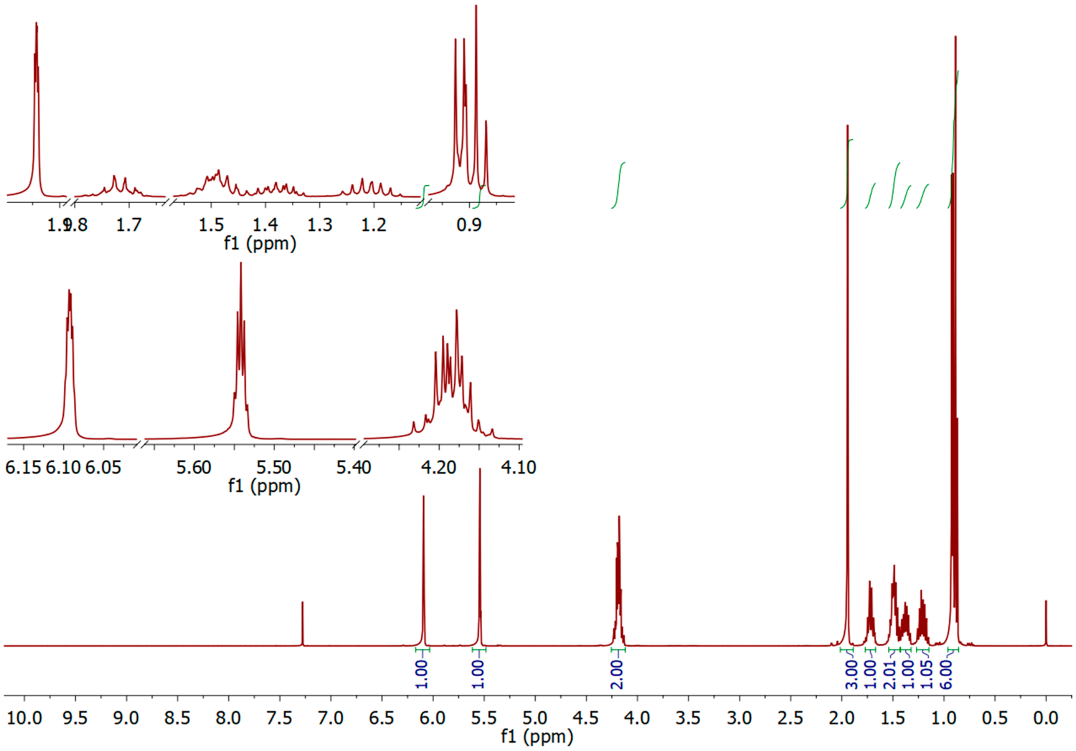

Figure S5. ^{1}H NMR spectrum of 3-methylpentyl methacrylate in $CDCl_3$ (400 MHz)

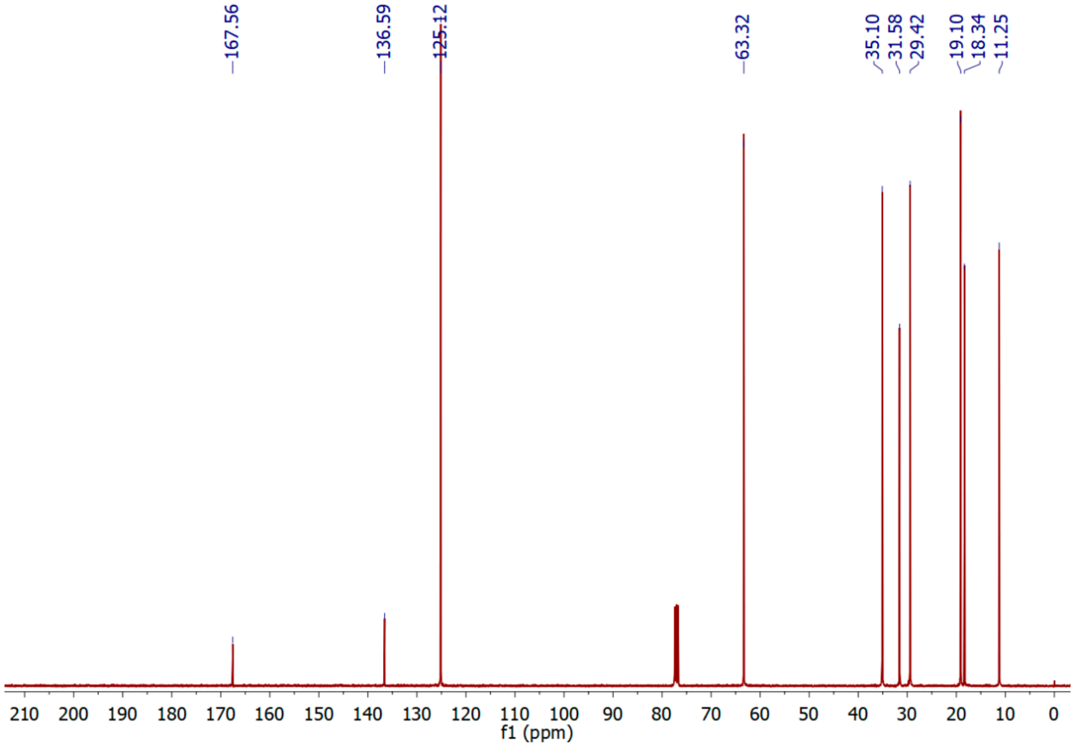

Figure S6. ^{13}C NMR spectrum of 3-methylpentyl methacrylate in $CDCl_3$ (101 MHz)

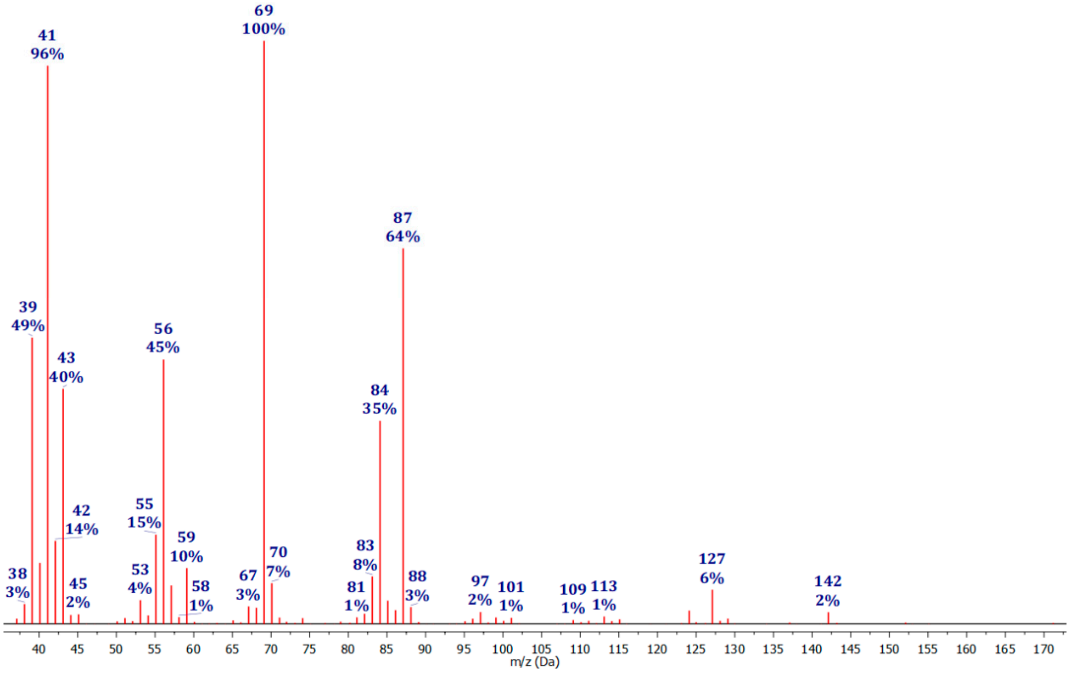

Figure S7. Mass spectrum of 4-methylpentyl methacrylate (**1d**; RI = 1136 (DB-5MS column))

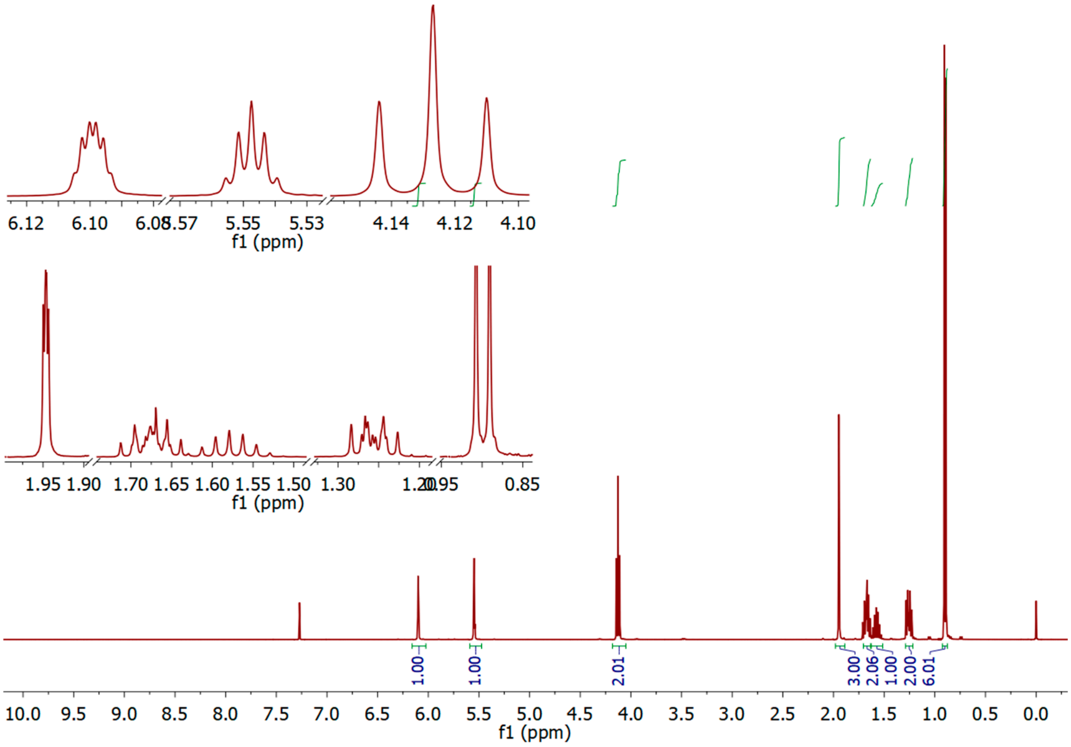

Figure S8. ^{1}H NMR spectrum of 4-methylpentyl methacrylate in $CDCl_3$ (400 MHz)

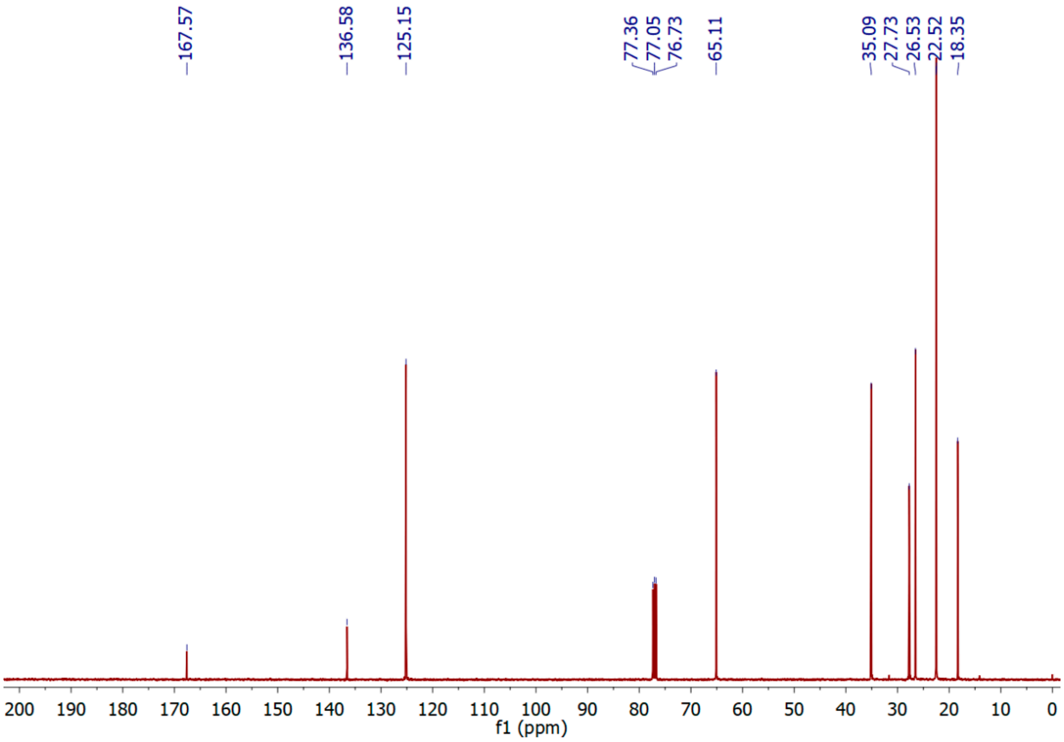

Figure S9. ^{13}C NMR spectrum of 4-methylpentyl methacrylate in $CDCl_3$ (101 MHz)

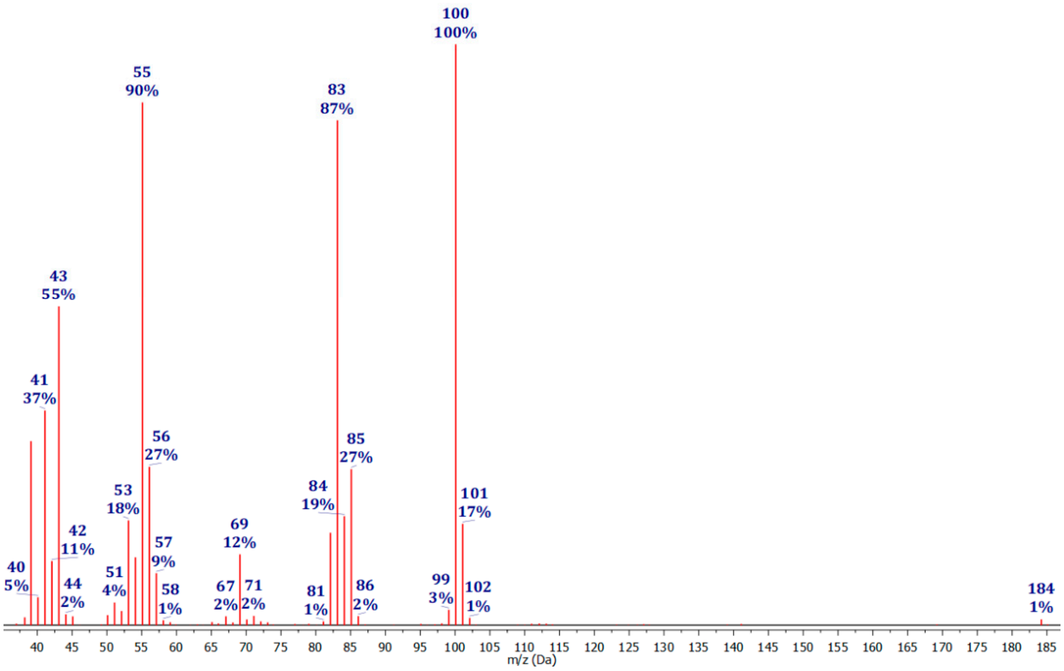

Figure S10. Mass spectrum of 2-methylpentyl angelate (**2b**; RI = 1240 (DB-5MS column))

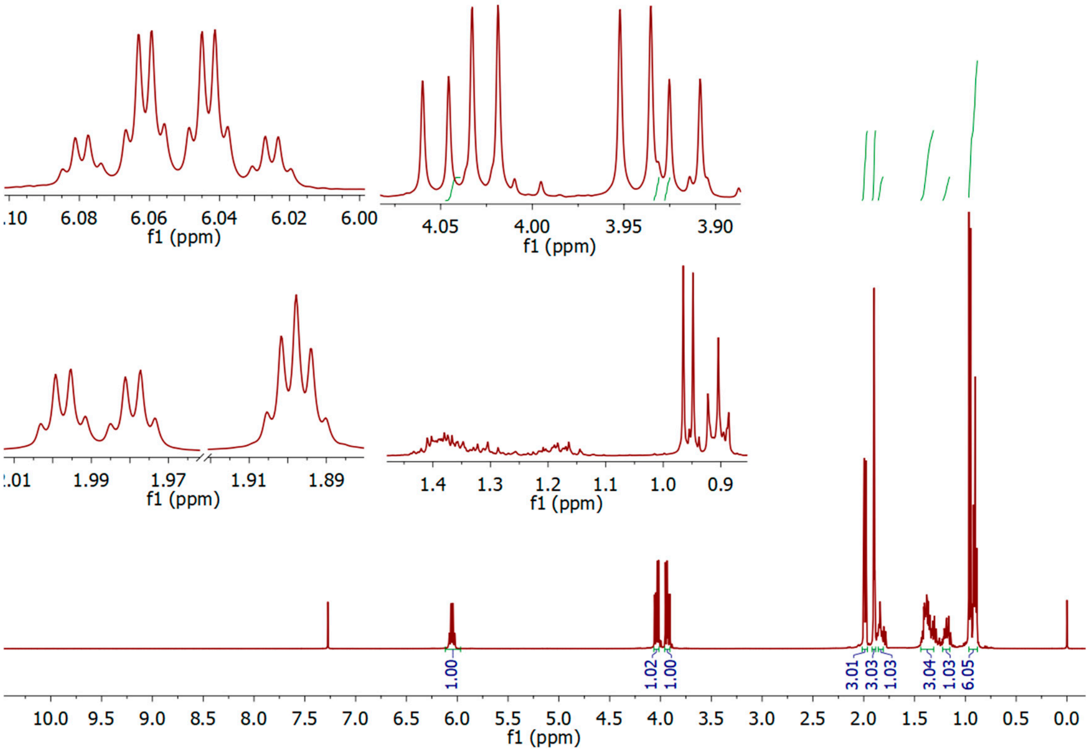

Figure S11. ^{1}H NMR spectrum of 2-methylpentyl angelate in $CDCl_3$ (400 MHz)

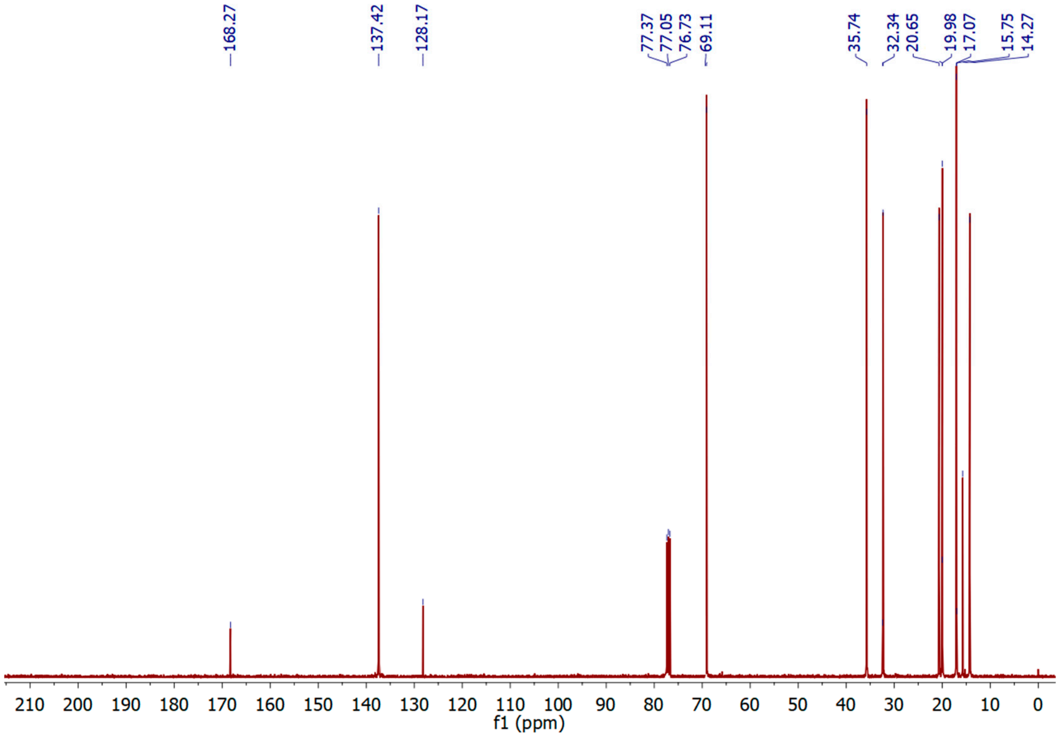

Figure S12. ^{13}C NMR spectrum of 2-methylpentyl angelate in $CDCl_3$ (101 MHz)

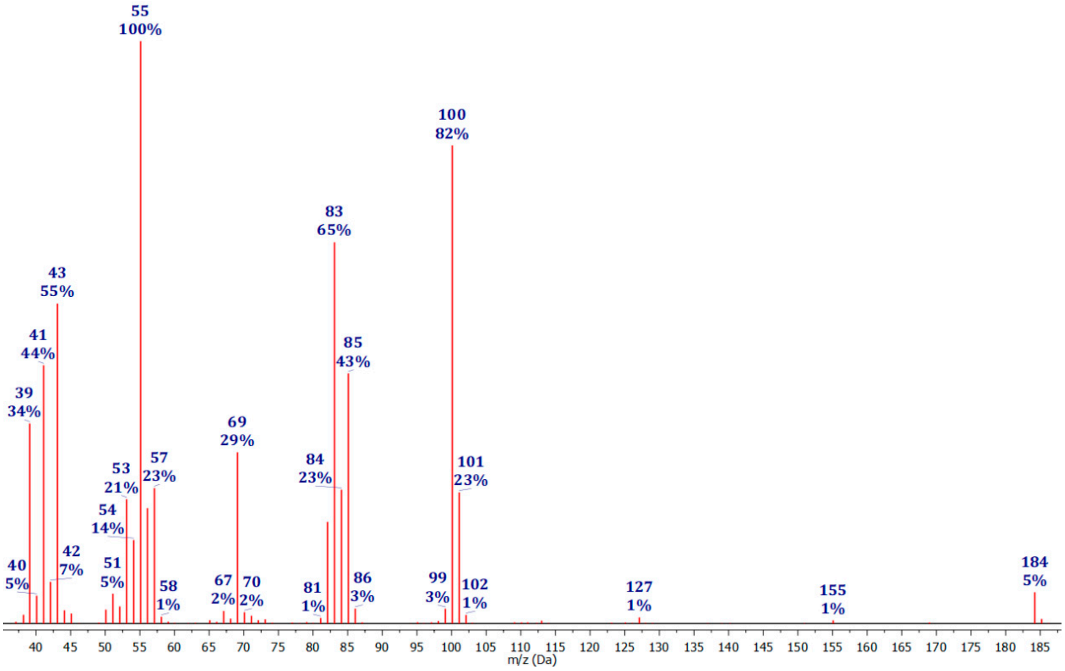

Figure S13. Mass spectrum of 3-methylpentyl angelate (**2c**; RI = 1258 (DB-5MS column))

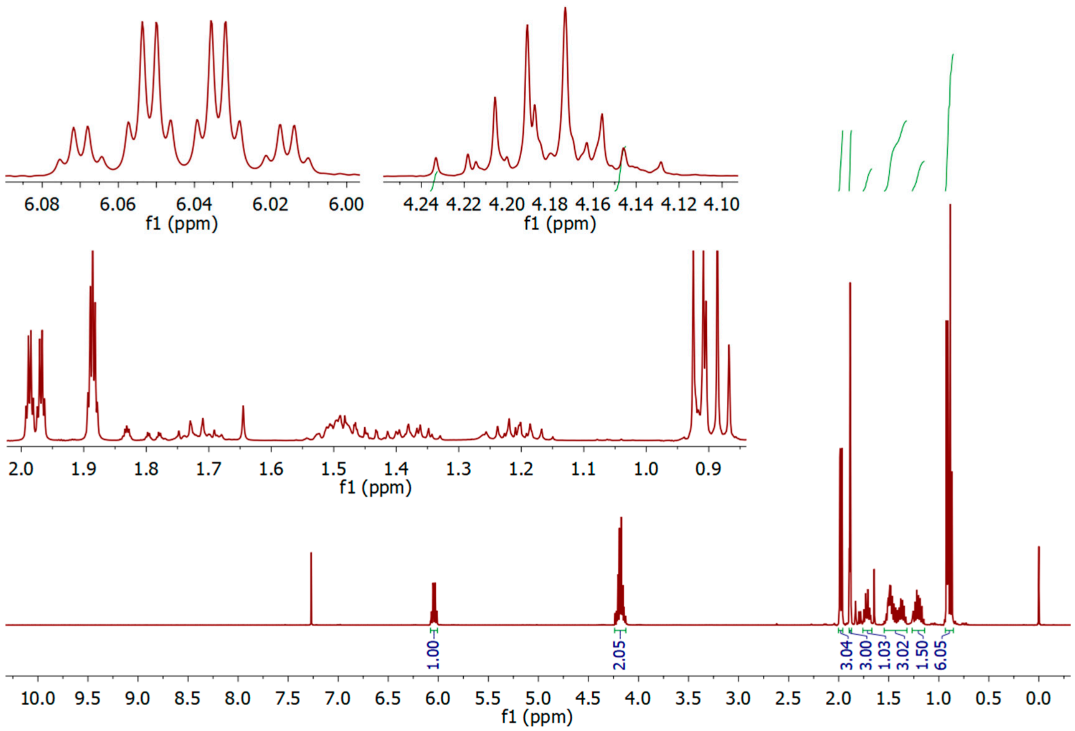

Figure S14. ^{1}H NMR spectrum of 3-methylpentyl angelate in $CDCl_3$ (400 MHz)

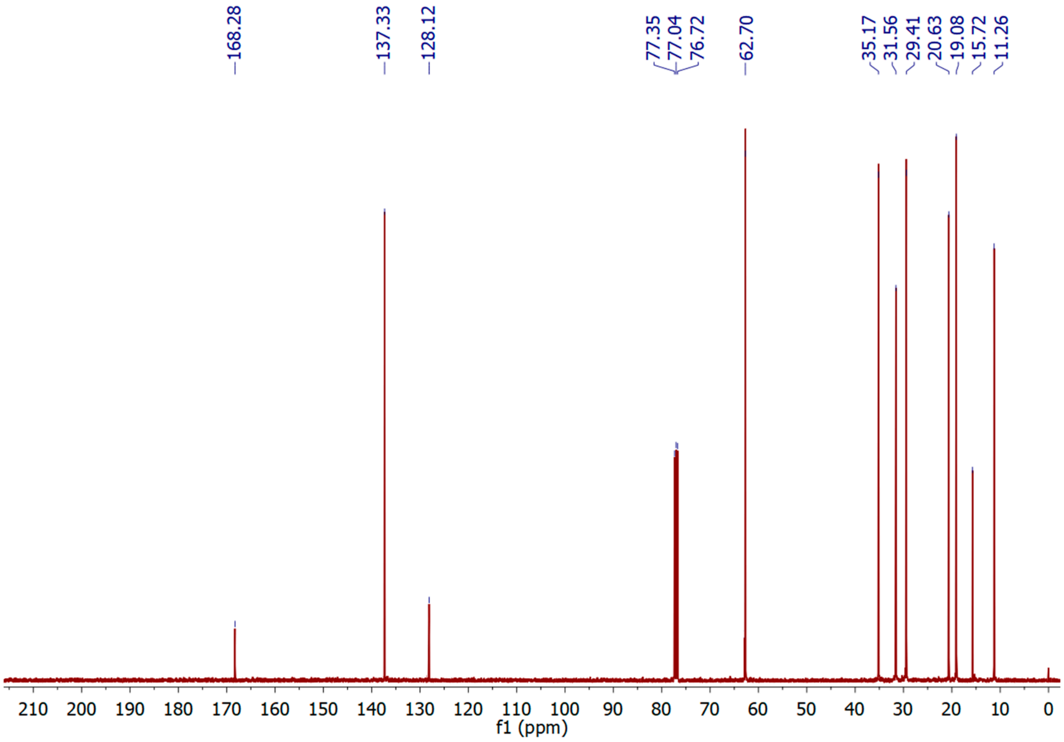

Figure S15. ^{13}C NMR spectrum of 3-methylpentyl angelate in $CDCl_3$ (101 MHz)

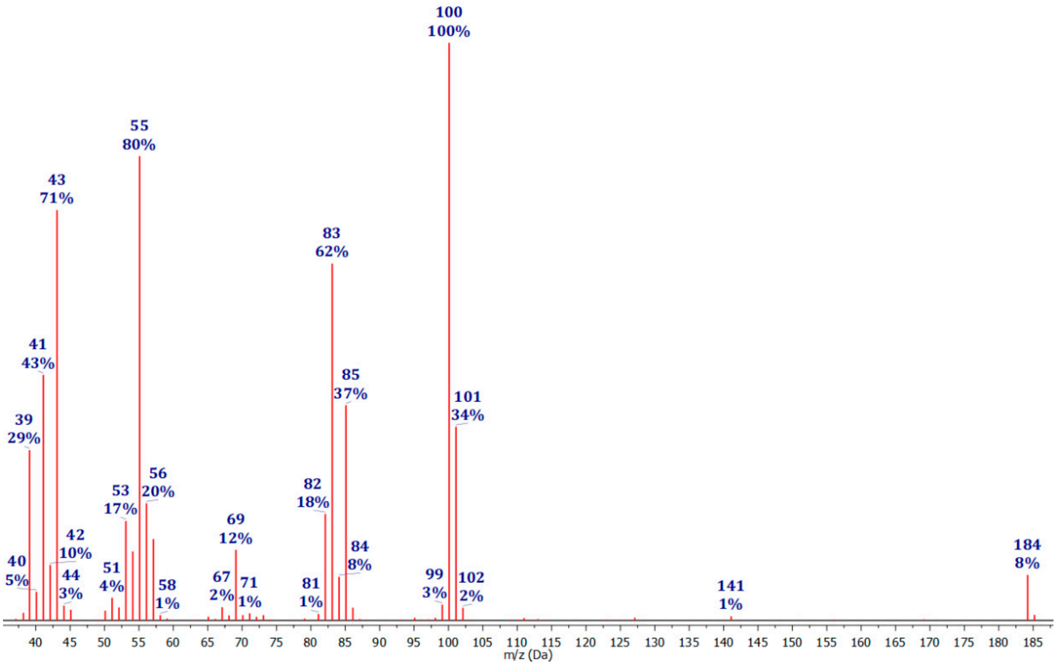

Figure S16. Mass spectrum of 4-methylpentyl angelate (**2d**; RI = 1249 (DB-5MS column))

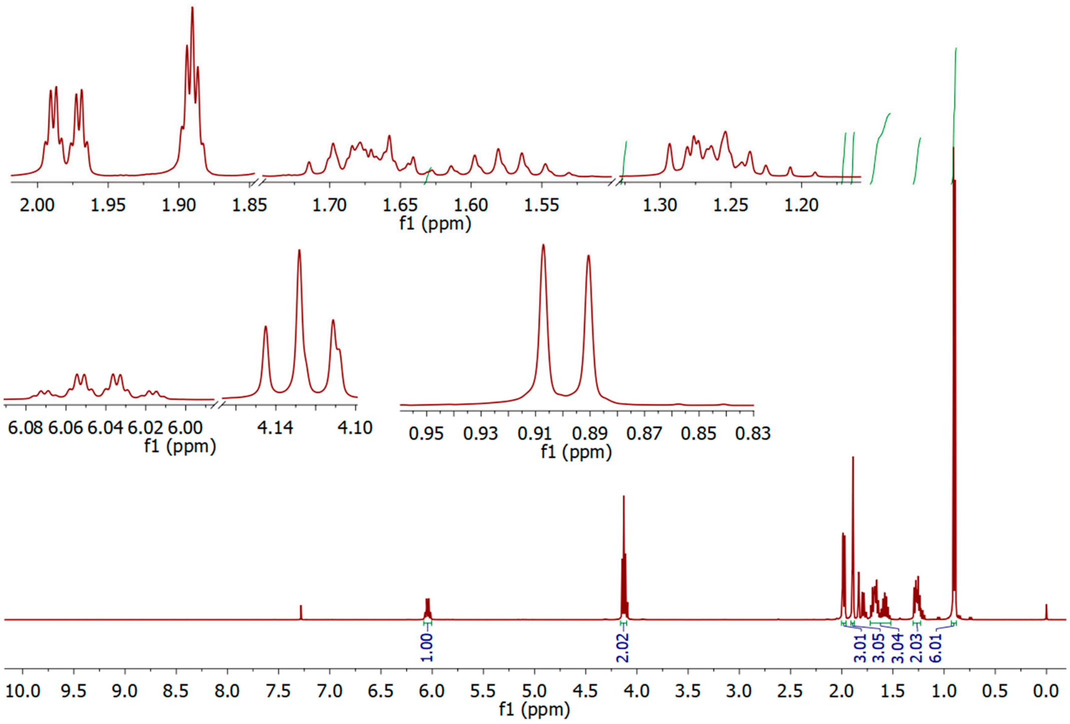

Figure S17. ^{1}H NMR spectrum of 4-methylpentyl angelate in $CDCl_3$ (400 MHz)

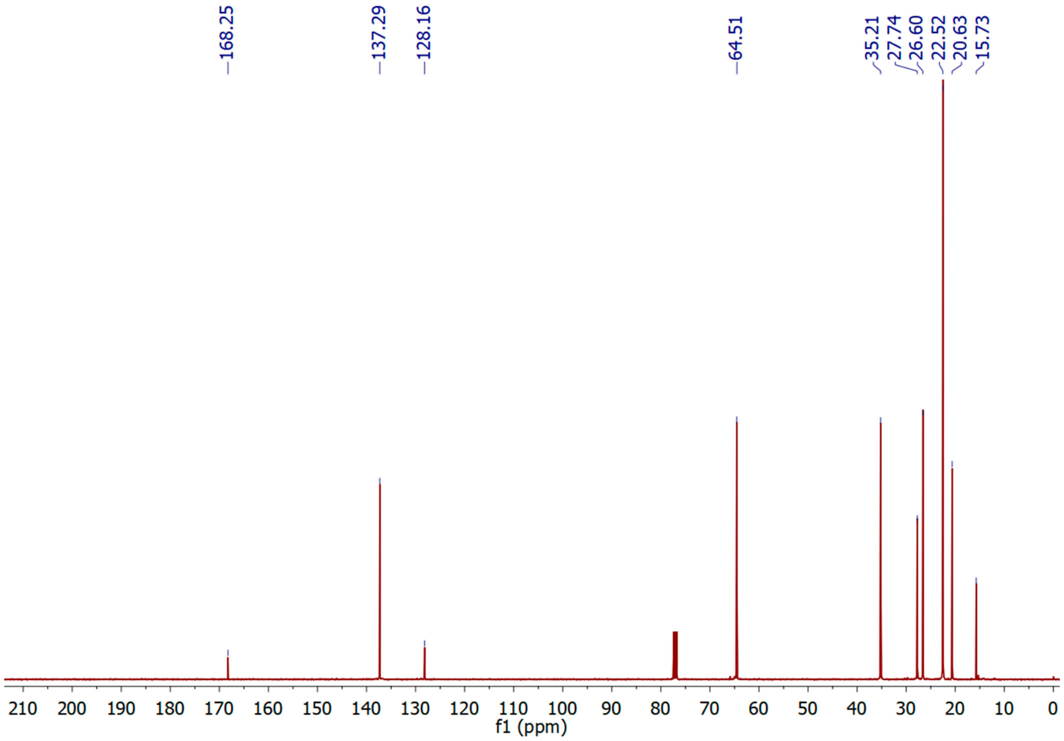

Figure S18. ^{13}C NMR spectrum of 4-methylpentyl angelate in $CDCl_3$ (101 MHz)

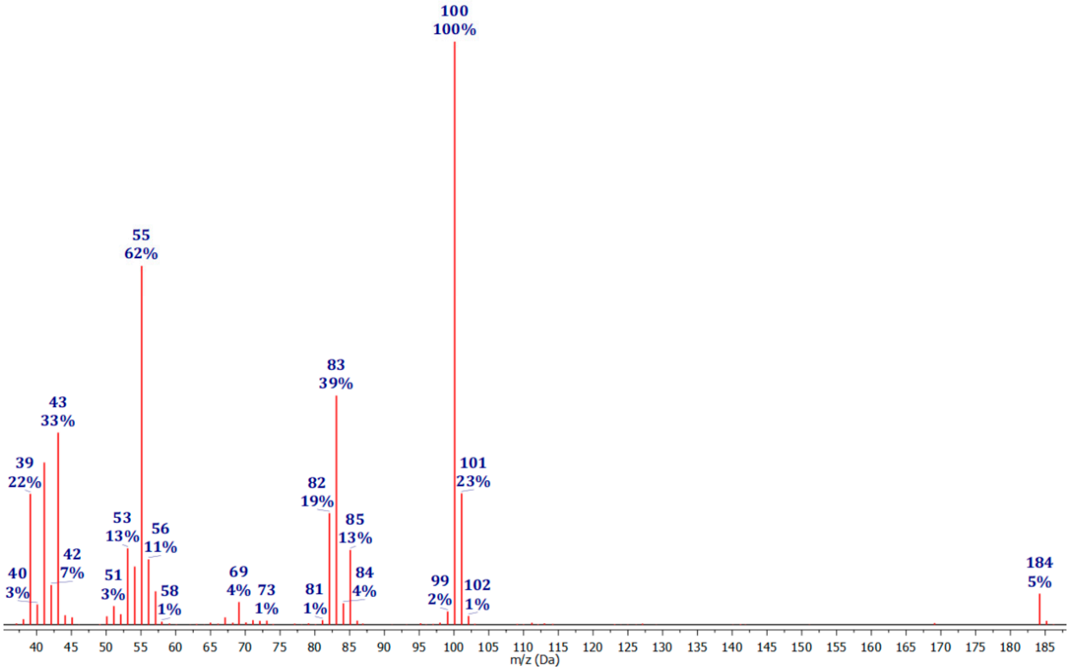

Figure S19. Mass spectrum of hexyl angelate (**2e**; RI = 1285 (DB-5MS column))

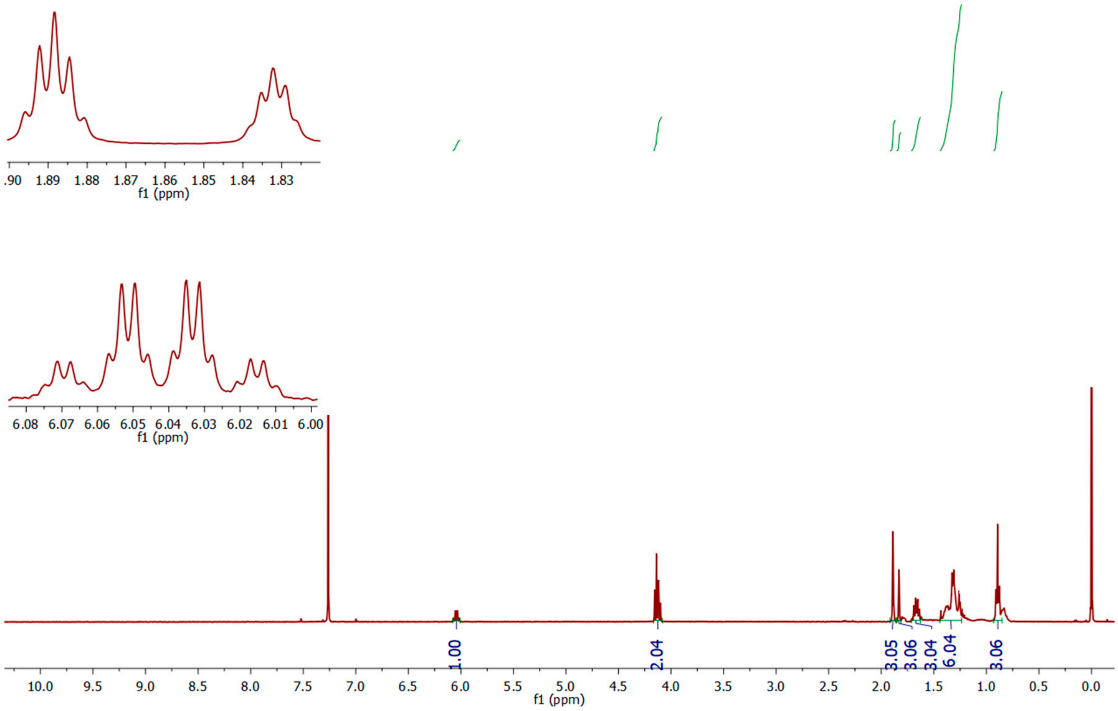

Figure S20. ^1H NMR spectrum of hexyl angelate in $CDCl_3$ (400 MHz)

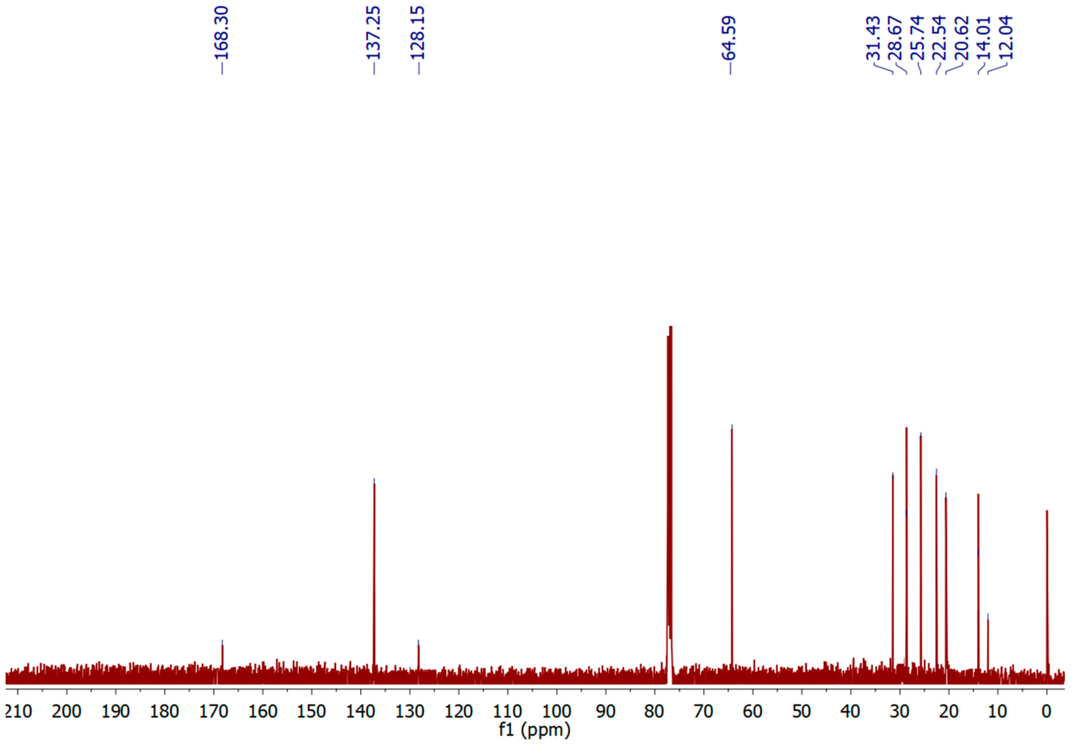

Figure S21. ^{13}C NMR spectrum of hexyl angelate in $CDCl_3$ (101 MHz)

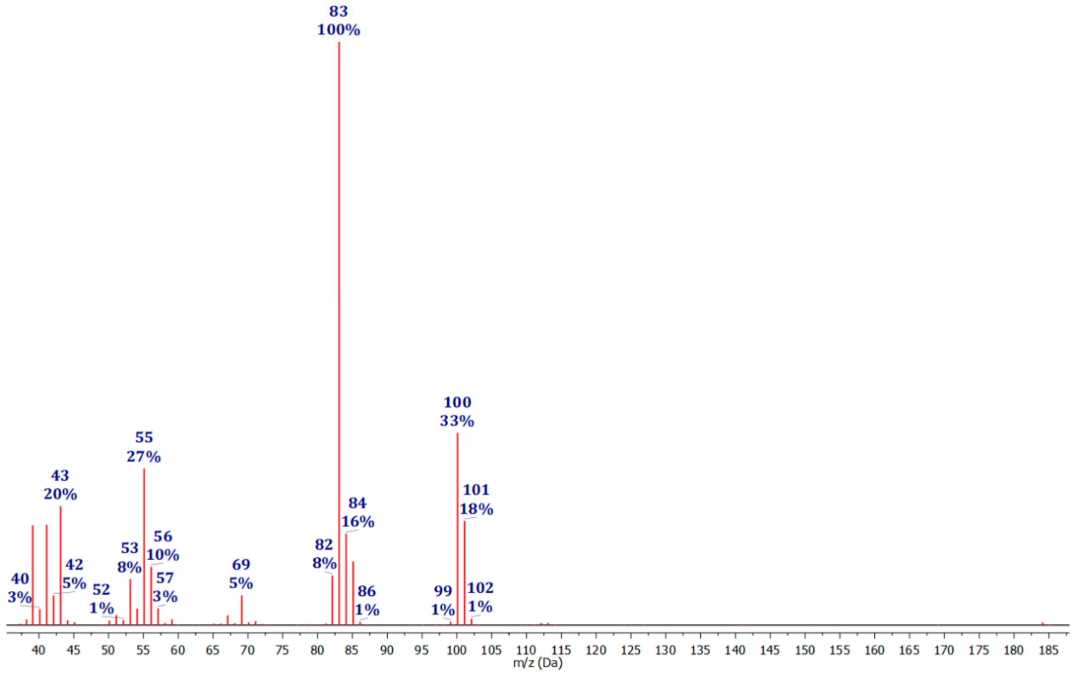

Figure S22. Mass spectrum of 2-methylpentyl senecioate (**3b**; RI = 1280 (DB-5MS column))

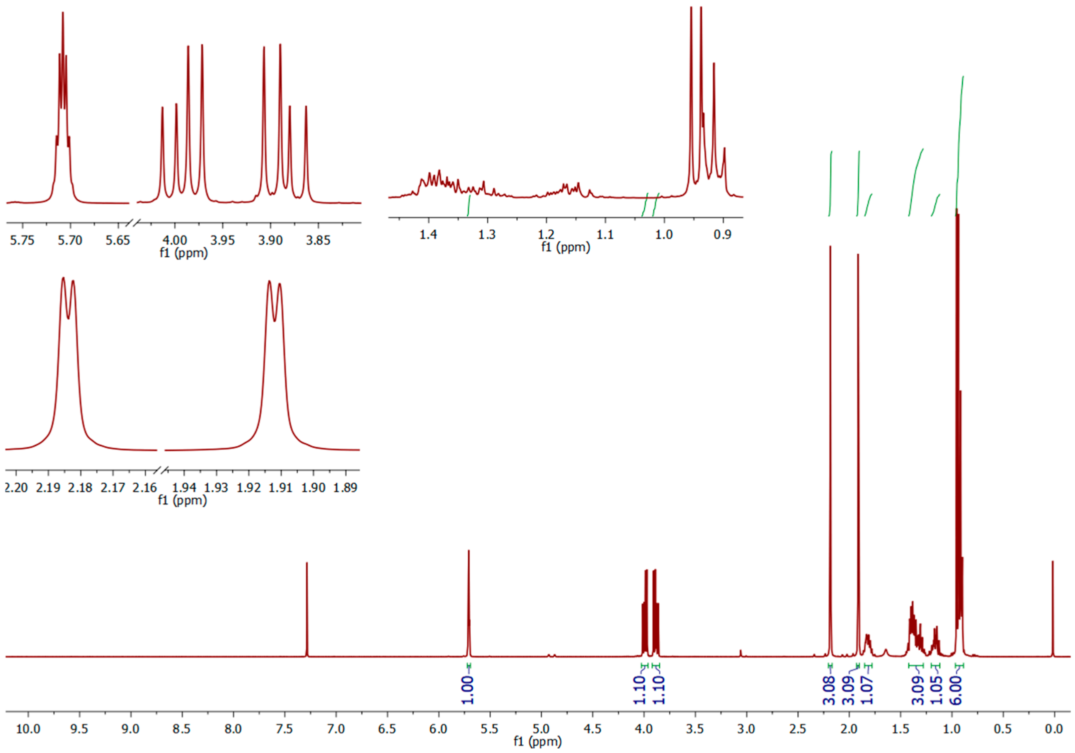

Figure S23. ^{1}H NMR spectrum of 2-methylpentyl senecioate in $CDCl_3$ (400 MHz)

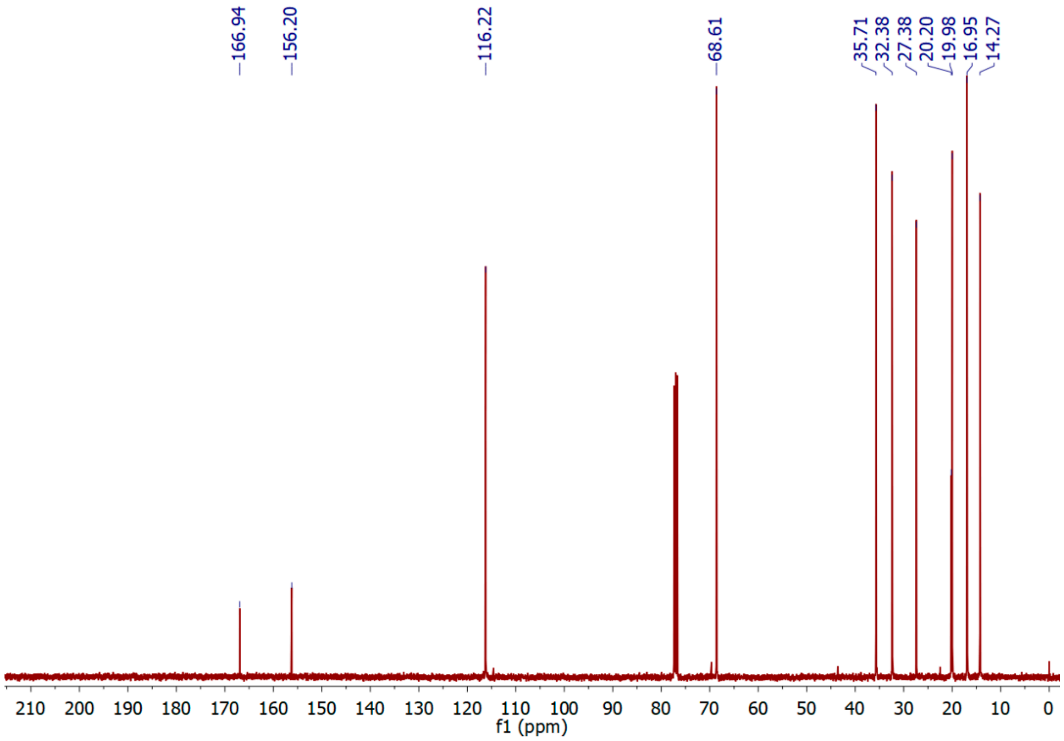

Figure S24. ^{13}C NMR spectrum of 2-methylpentyl senecioate in $CDCl_3$ (101 MHz)

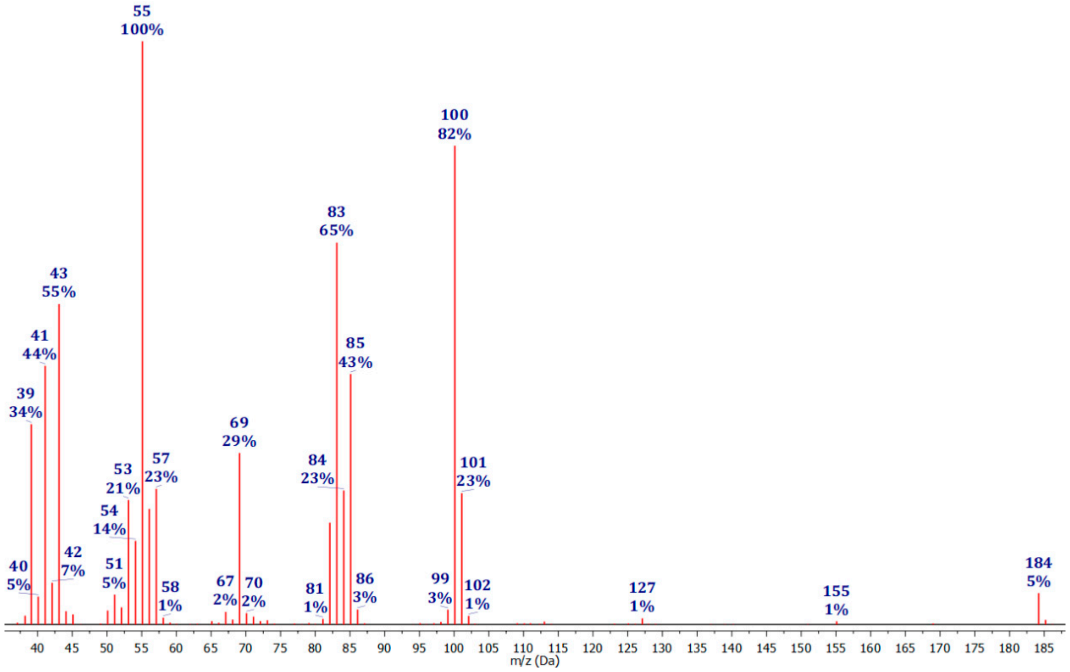

Figure S25. Mass spectrum of 3-methylpentyl senecioate (**3c**; RI = 1295 (DB-5MS column))

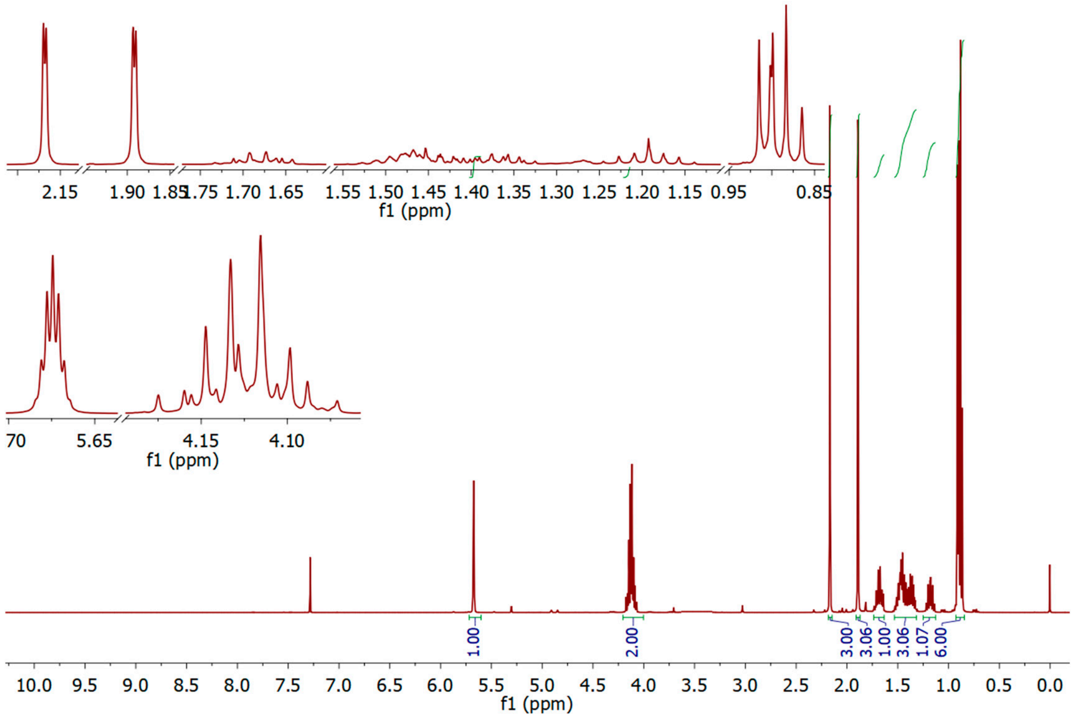

Figure S26. ^1H NMR spectrum of 3-methylpentyl senecioate in $CDCl_3$ (400 MHz)

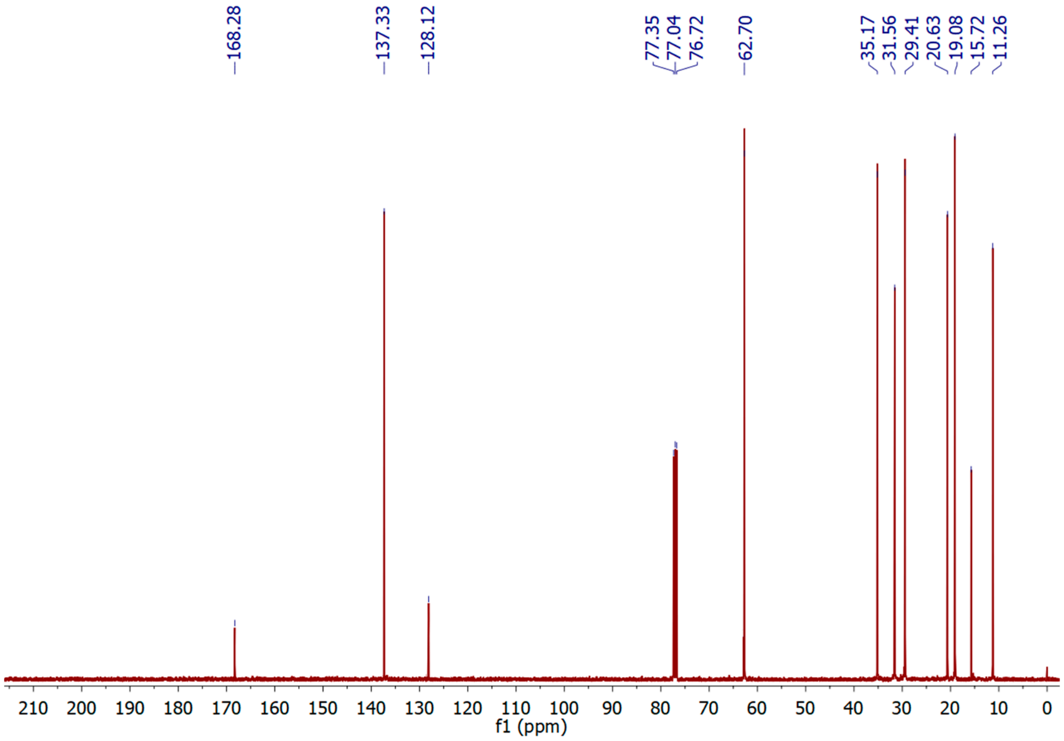

Figure S27. ^{13}C NMR spectrum of 3-methylpentyl senecioate in $CDCl_3$ (101 MHz)

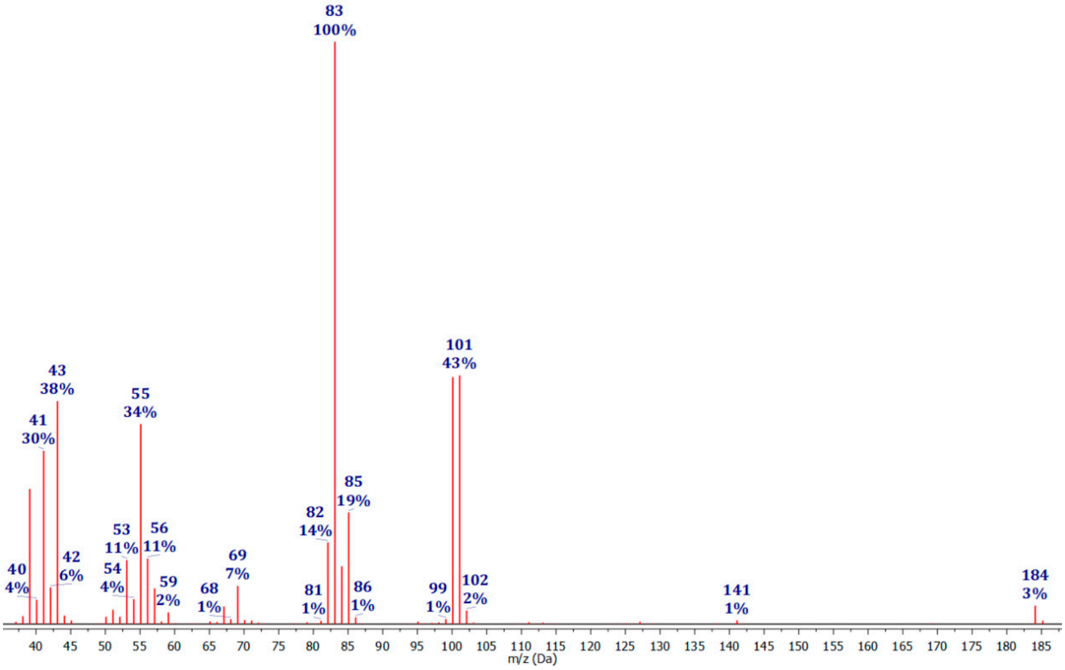

Figure S28. Mass spectrum of 4-methylpentyl senecioate (**3d**; RI = 1289 (DB-5MS column))

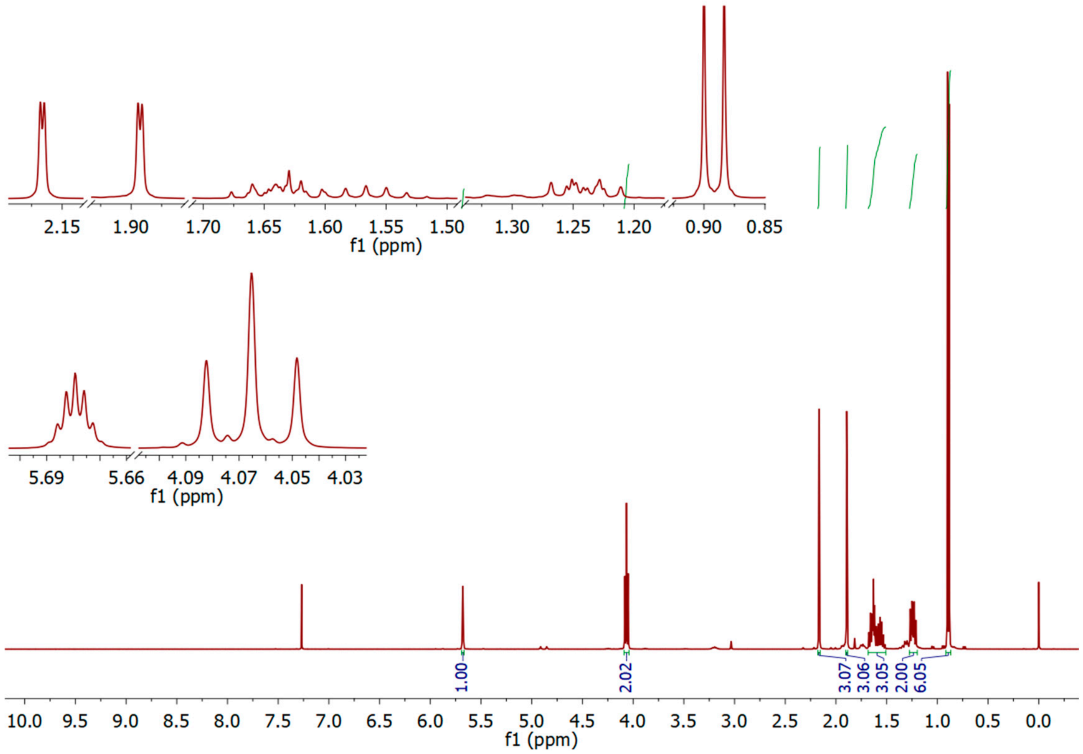

Figure S29. ^{1}H NMR spectrum of 4-methylpentyl senecioate in $CDCl_3$ (400 MHz)

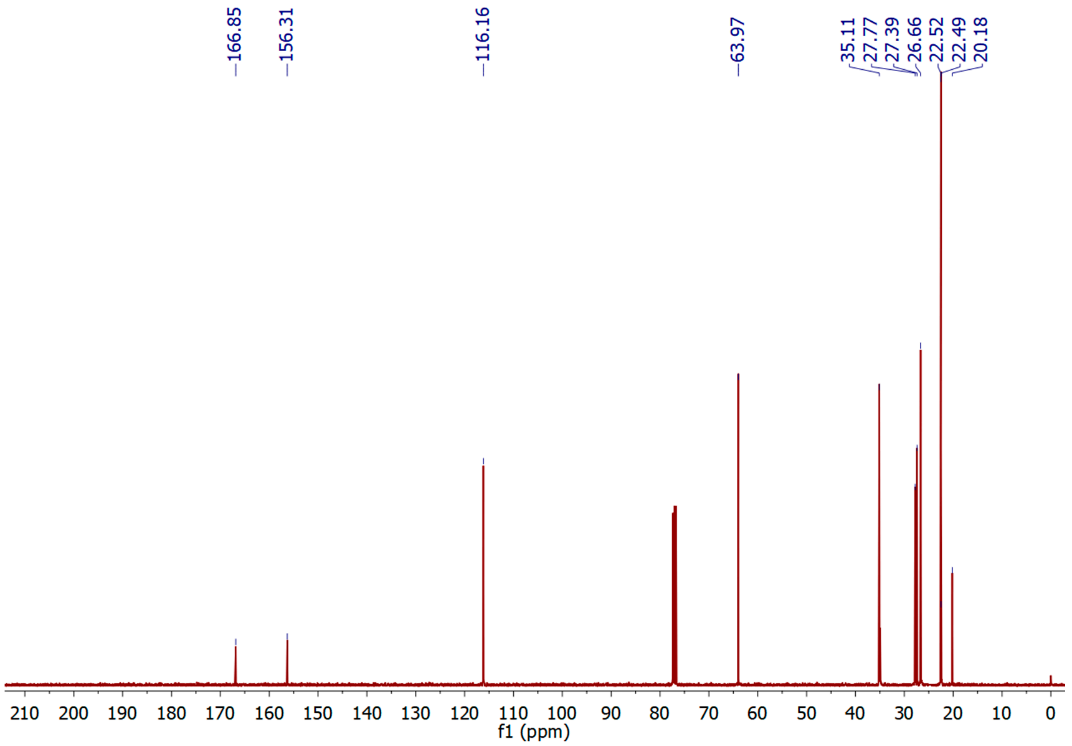

Figure S30. ^{13}C NMR spectrum of 4-methylpentyl senecioate in $CDCl_3$ (101 MHz)

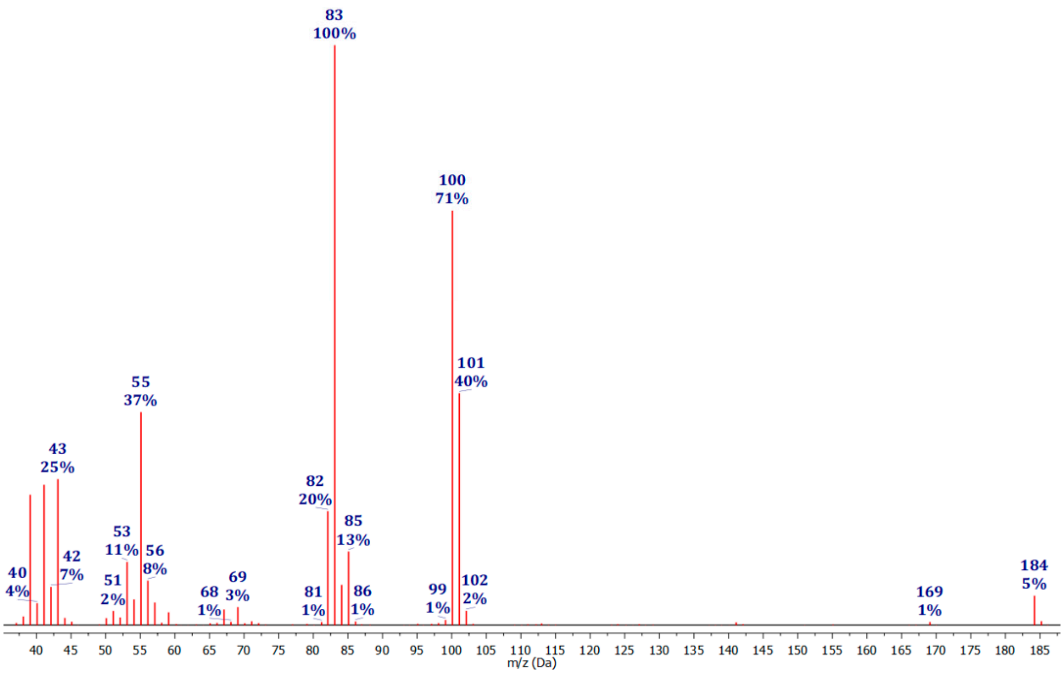

Figure S31. Mass spectrum of hexyl senecioate (**3e**; RI = 1325 (DB-5MS column))

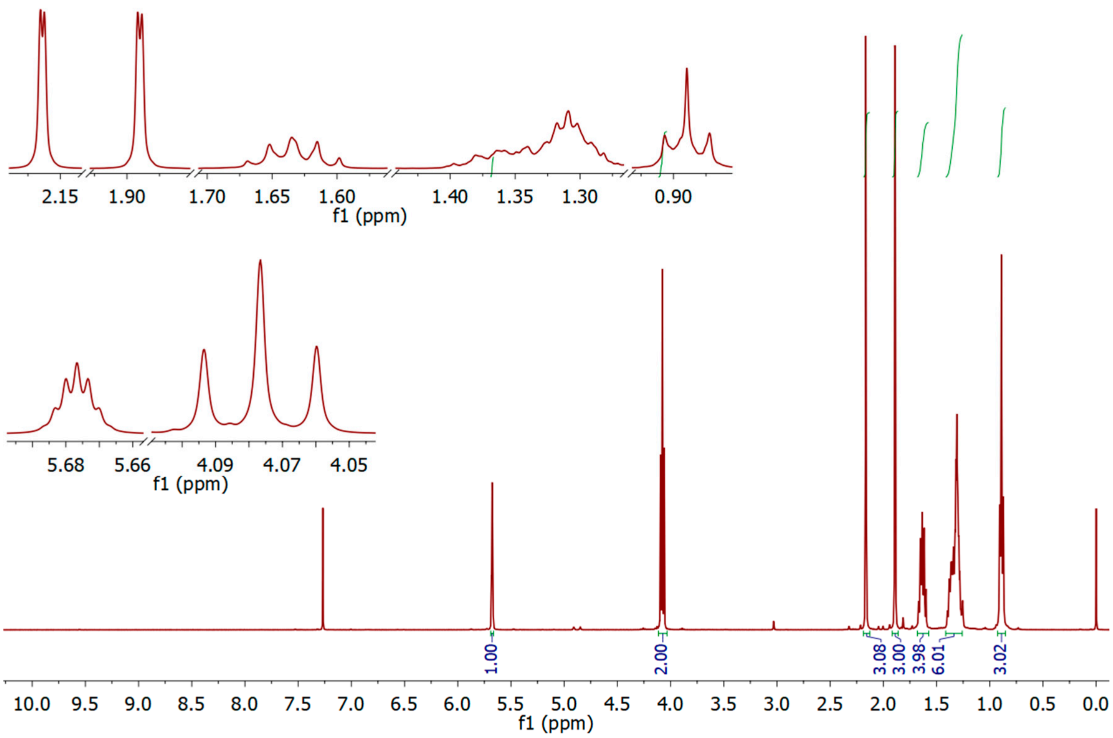

Figure S32. ^{1}H NMR spectrum of hexyl senecioate in $CDCl_3$ (400 MHz)

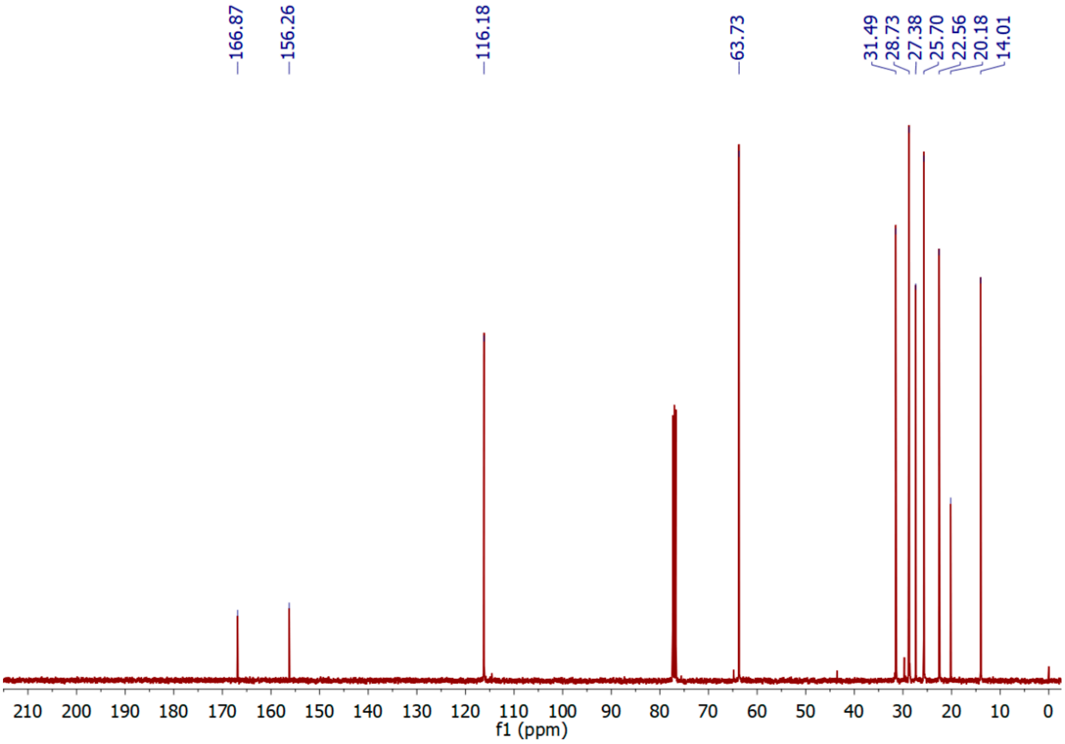

Figure S33. ^{13}C NMR spectrum of hexyl senecioate in $CDCl_3$ (101 MHz)

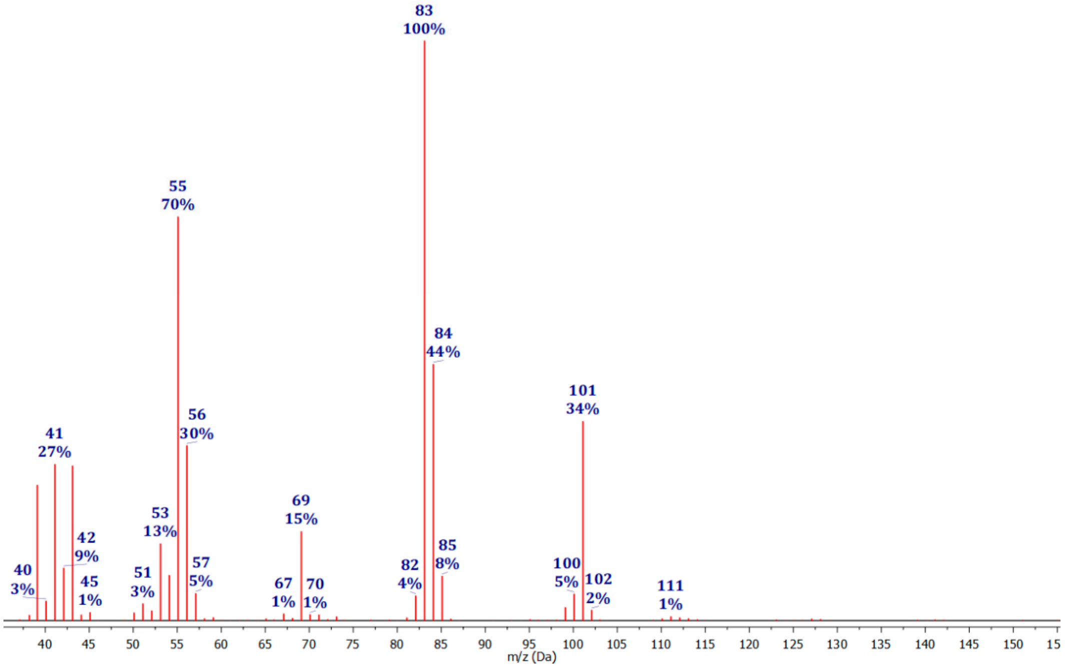

Figure S34. Mass spectrum of 2-methylpentyl tiglate (**4b**; RI = 1289 (DB-5MS column))

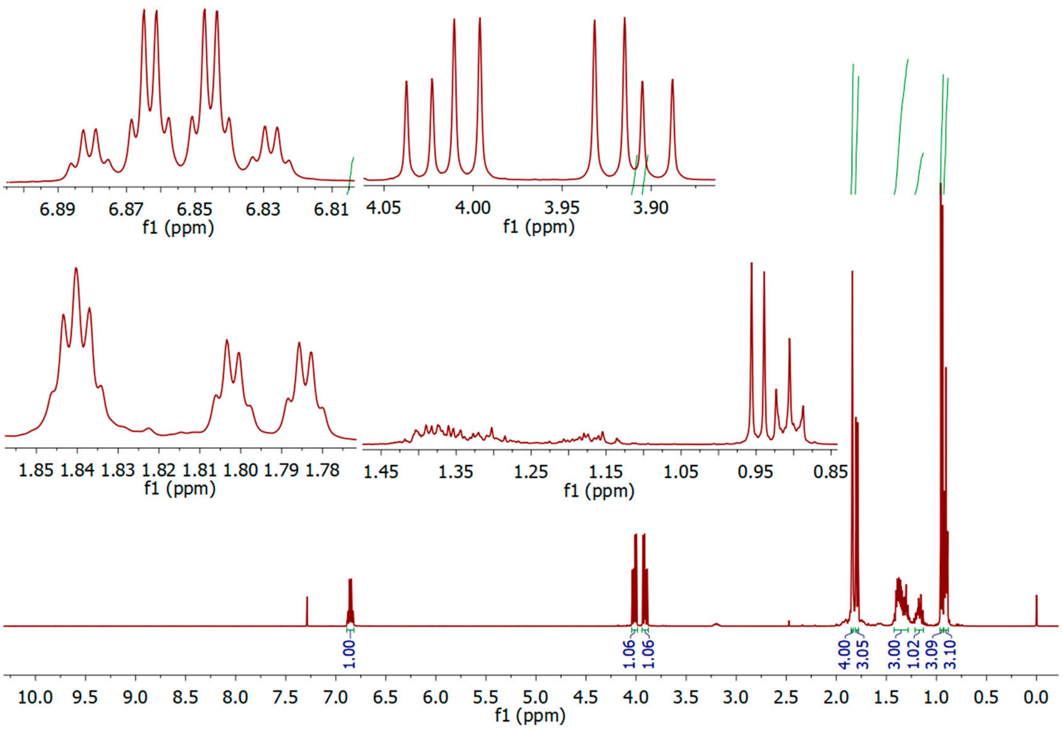

Figure S35. ^{1}H NMR spectrum of 2-methylpentyl tiglate in $CDCl_3$ (400 MHz)

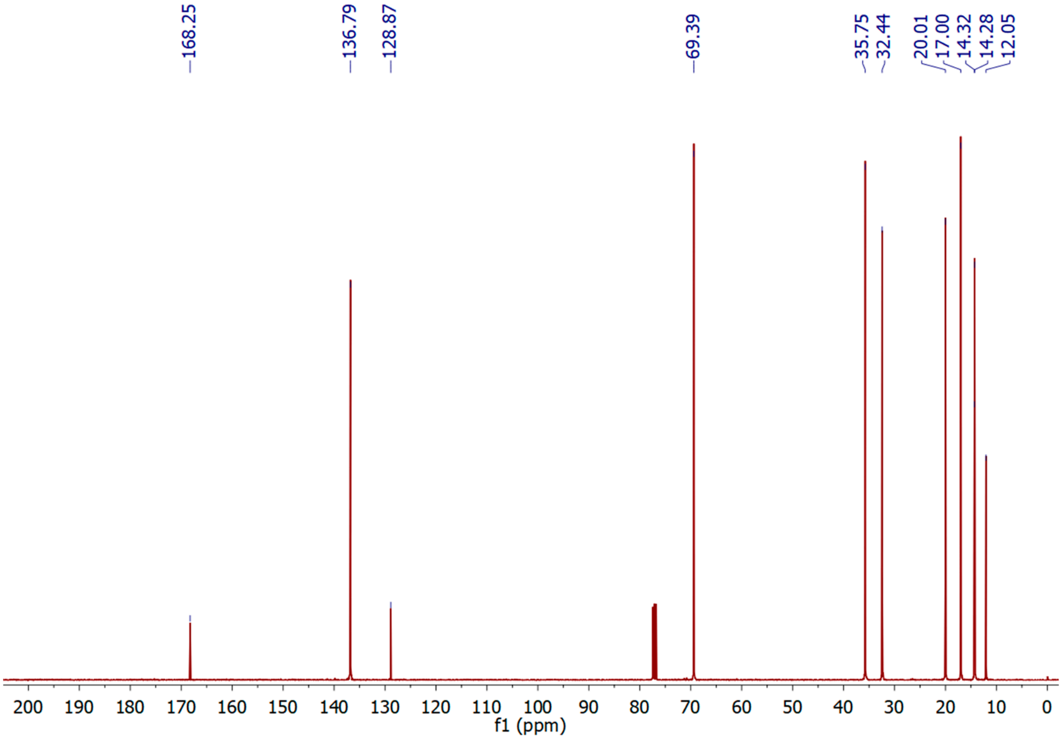

Figure S36. ^{13}C NMR spectrum of 2-methylpentyl tiglate in $CDCl_3$ (101 MHz)

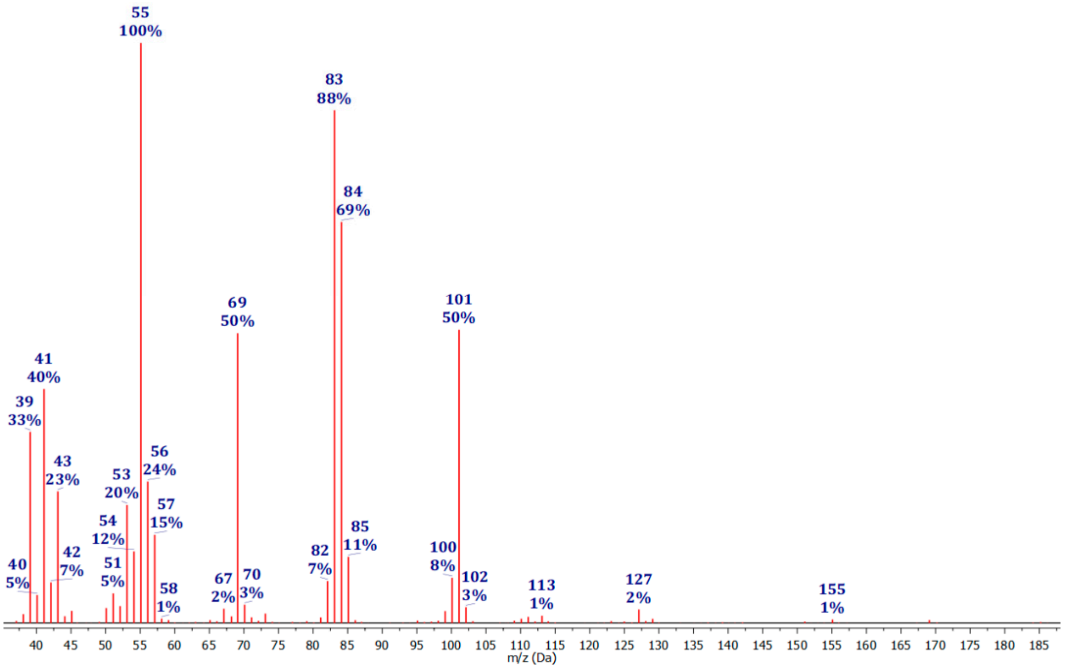

Figure S37. Mass spectrum of 3-methylpentyl tiglate (**4c**; RI = 1304 (DB-5MS column))

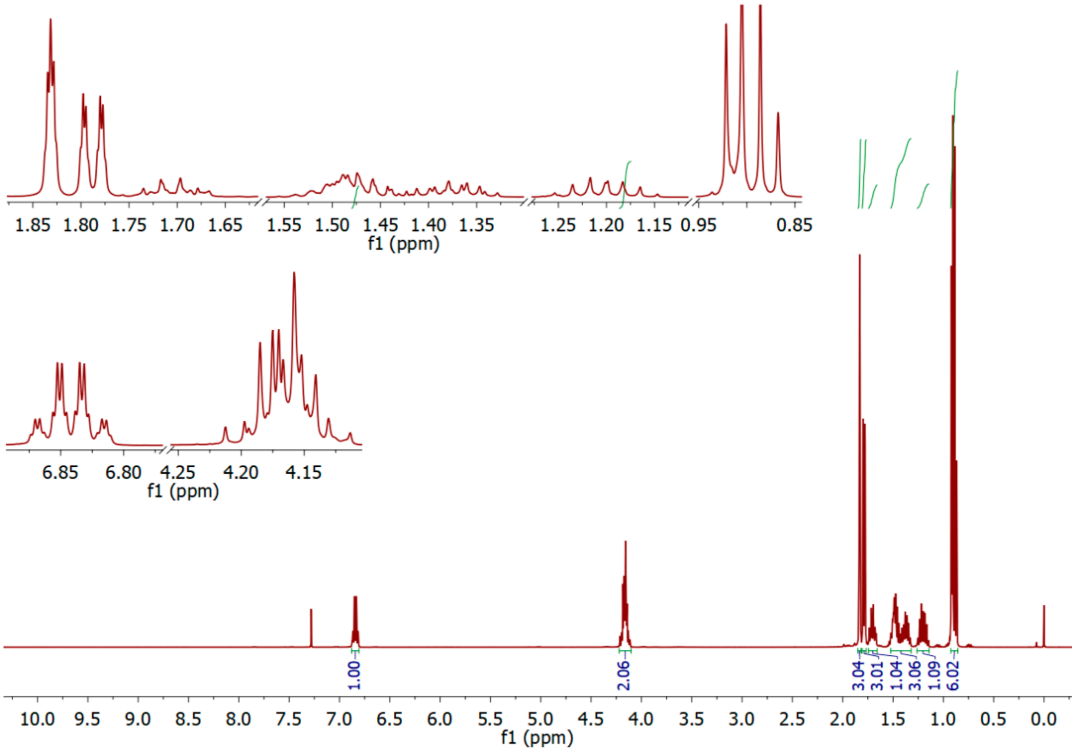

Figure S38. ^{1}H NMR spectrum of 3-methylpentyl tiglate in $CDCl_3$ (400 MHz)

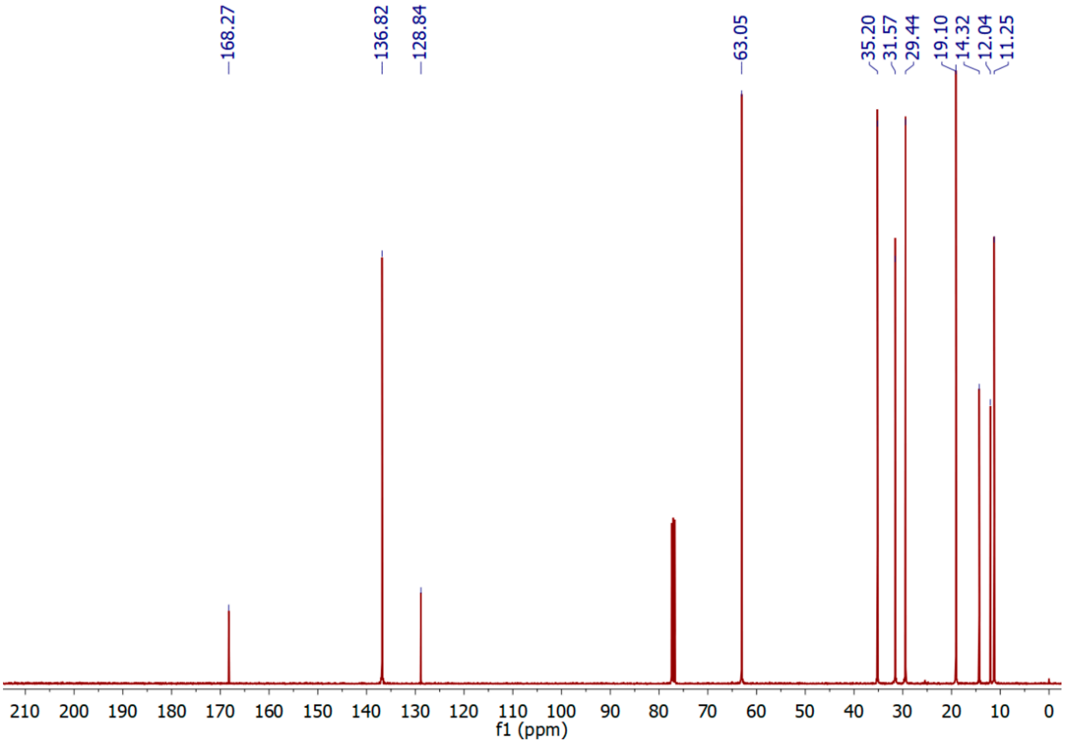

Figure S39. ^{13}C NMR spectrum of 3-methylpentyl tiglate in $CDCl_3$ (101 MHz)

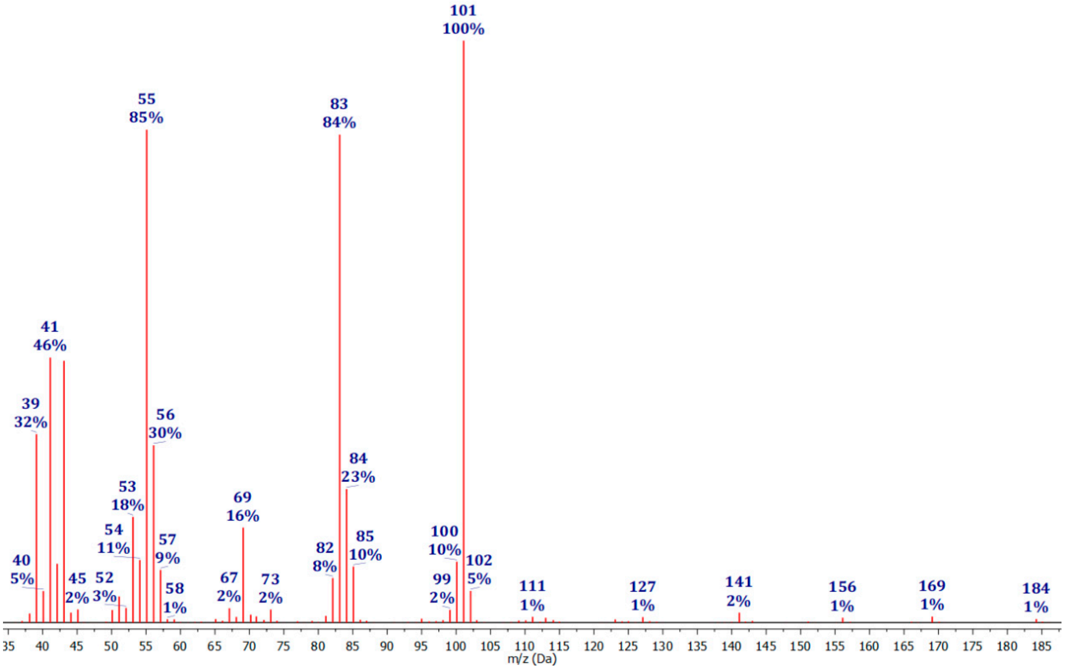

Figure S40. Mass spectrum of 4-methylpentyl tiglate (**4d**; RI = 1298 (DB-5MS column))

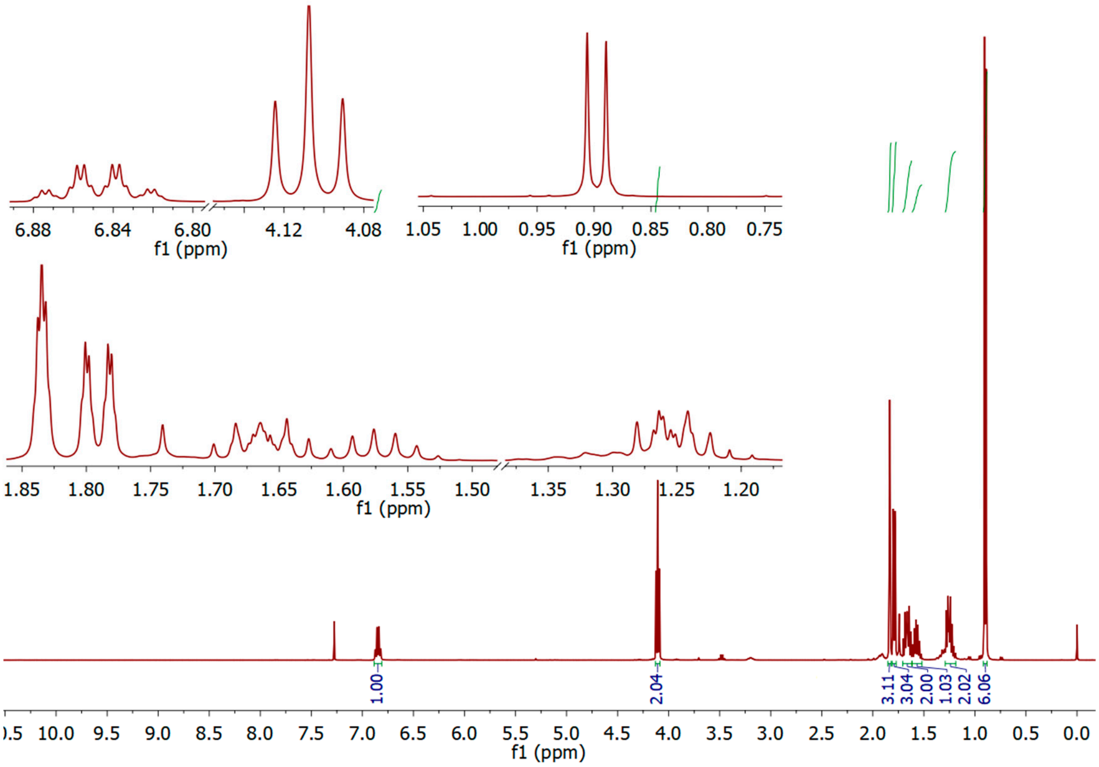

Figure S41. ^{1}H NMR spectrum of 4-methylpentyl tiglate in $CDCl_3$ (400 MHz)

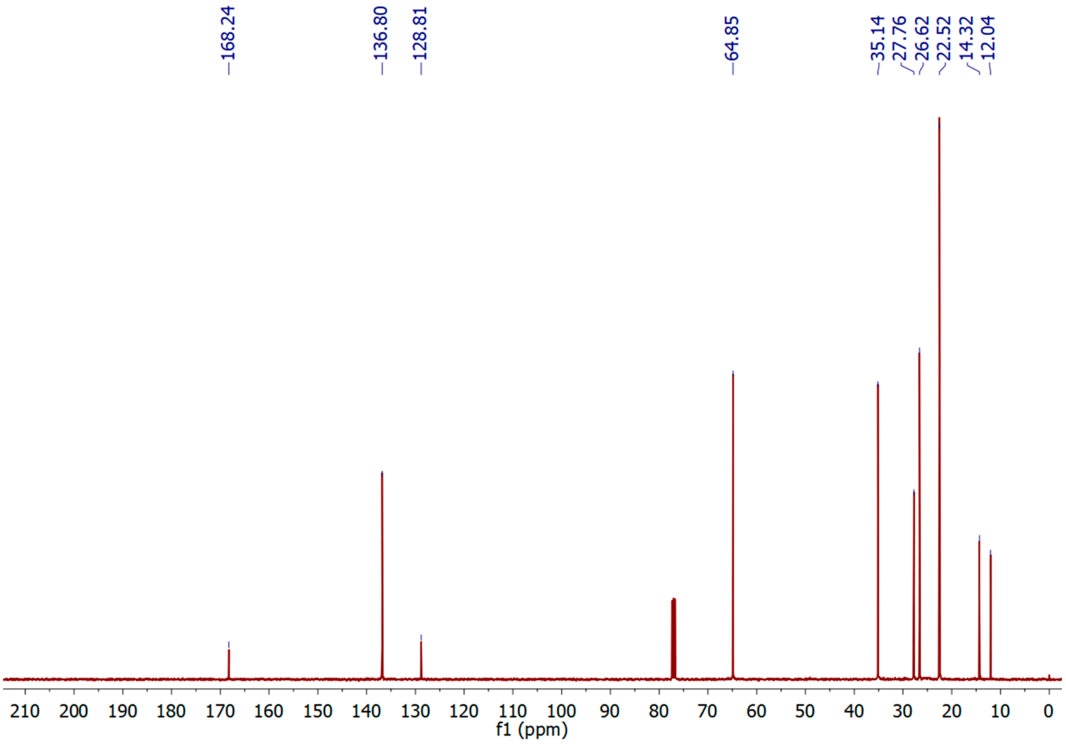

Figure S42. ^{13}C NMR spectrum of 4-methylpentyl tiglate in $CDCl_3$ (101 MHz)

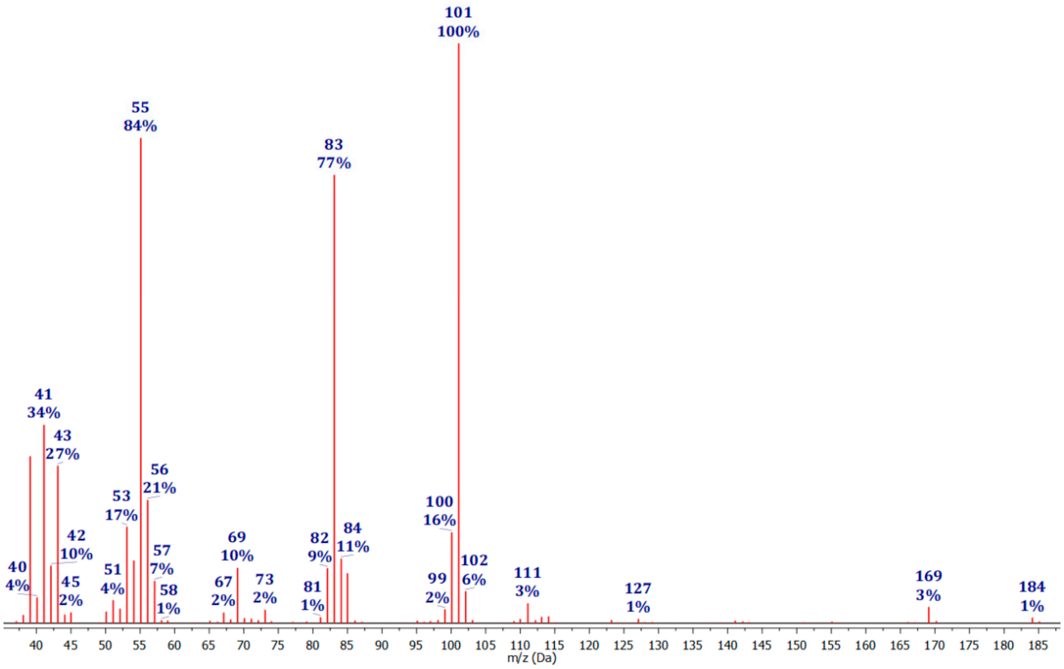

Figure S43. Mass spectrum of hexyl tiglate (**4e**; RI = 1334 (DB-5MS column))

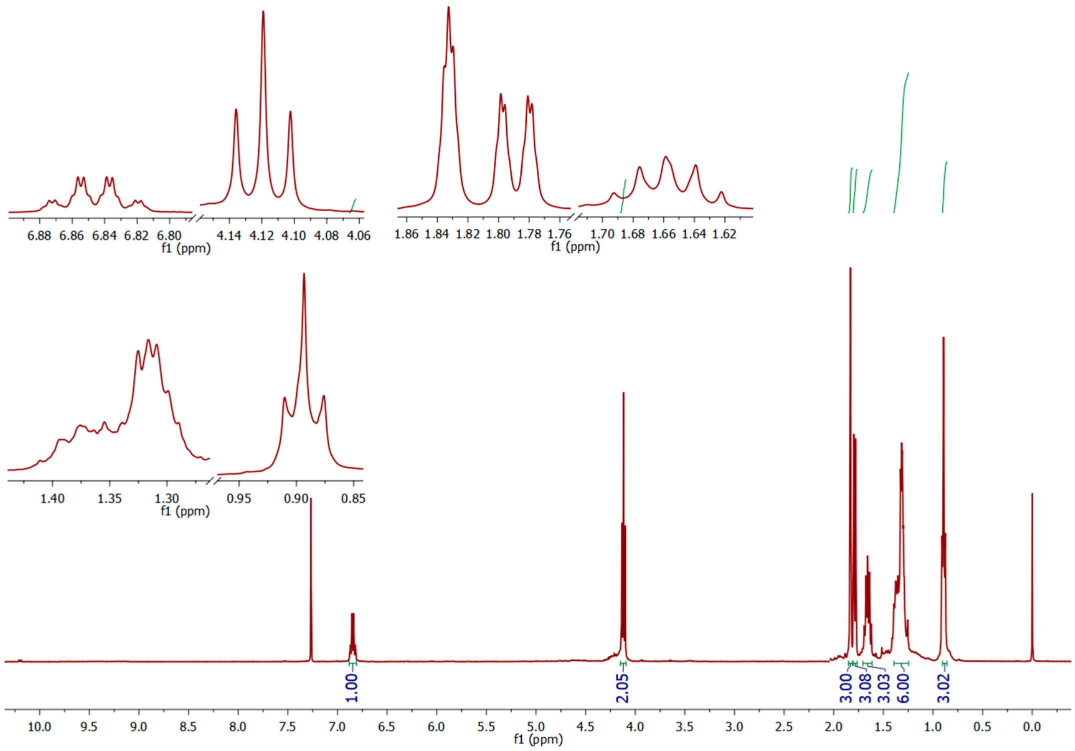

Figure S44. ^{1}H NMR spectrum of hexyl tiglate in $CDCl_3$ (400 MHz)

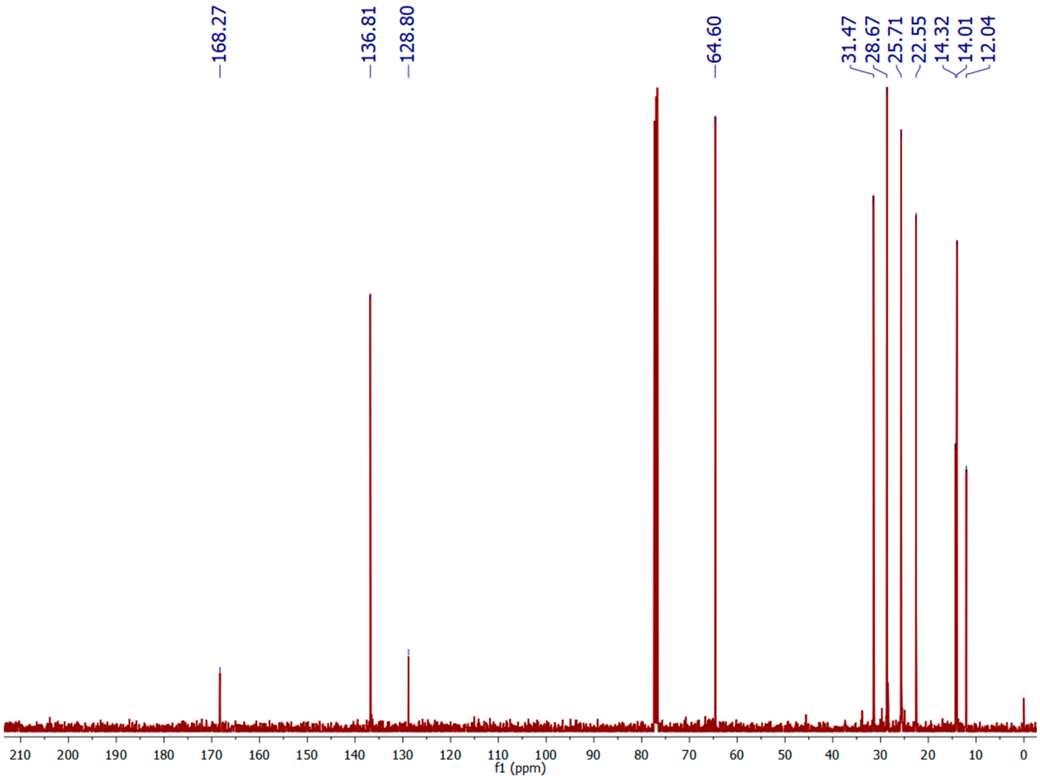

Figure S45. ^{13}C NMR spectrum of hexyl tiglate in $CDCl_3$ (101 MHz)

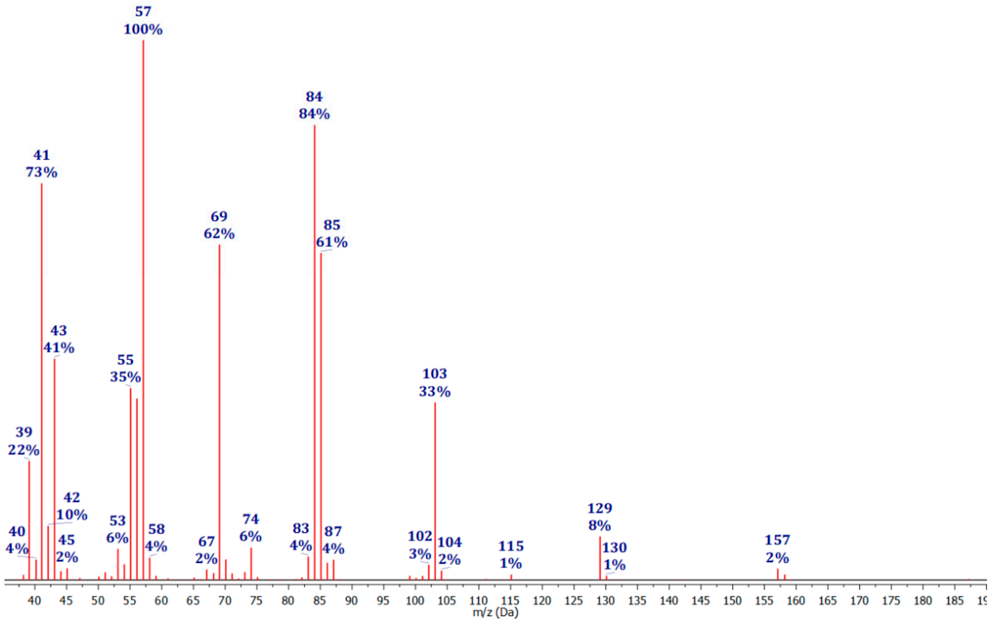

Figure S46. Mass spectrum of 3-methylpentyl 2-methylbutanoate (**5c**; RI = 1204 (DB-5MS column))

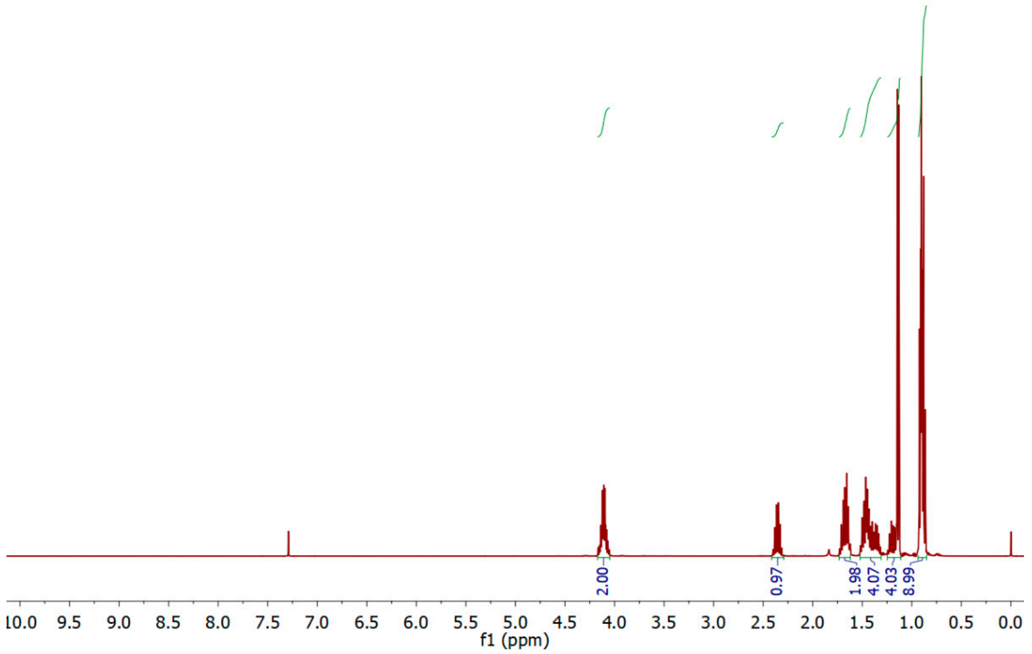

Figure S47. ^{1}H NMR spectrum of 3-methylpentyl 2-methylbutanoate in $CDCl_3$ (400 MHz)

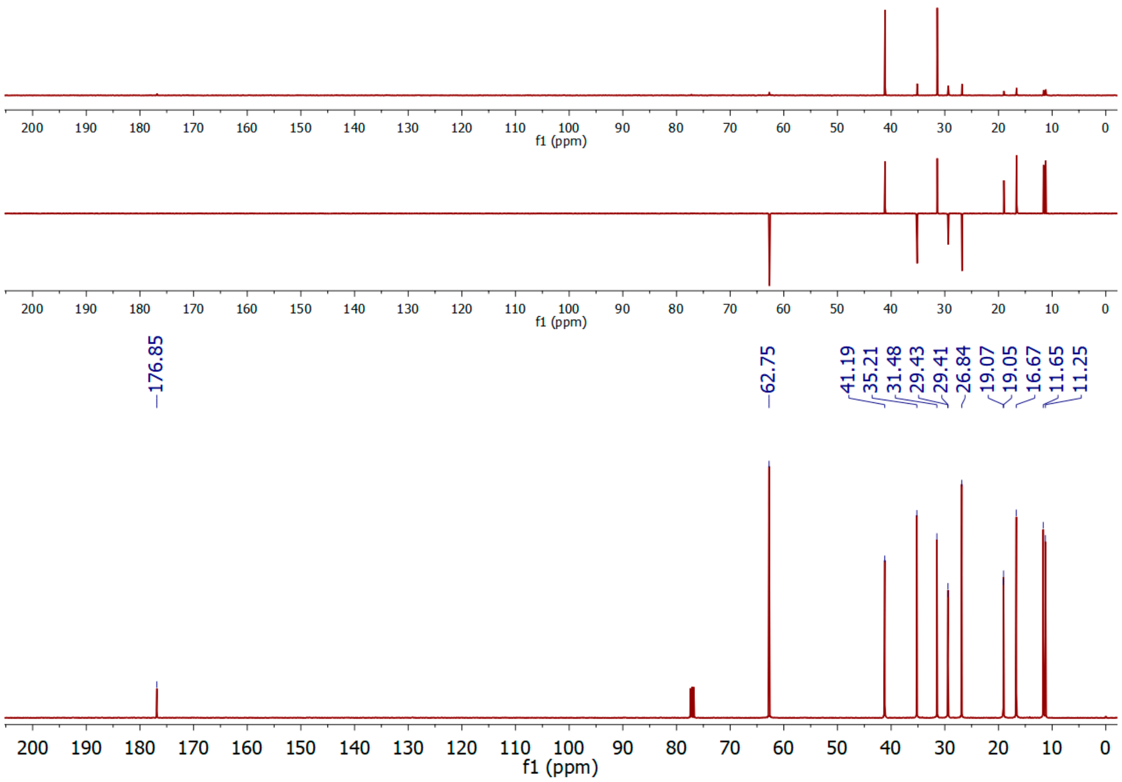

Figure S48a. DEPT-90, DEPT-135, and ^{13}C NMR spectra (from top to bottom) of 3-methylpentyl 2-methylbutanoate in $CDCl_3$ (101 MHz)

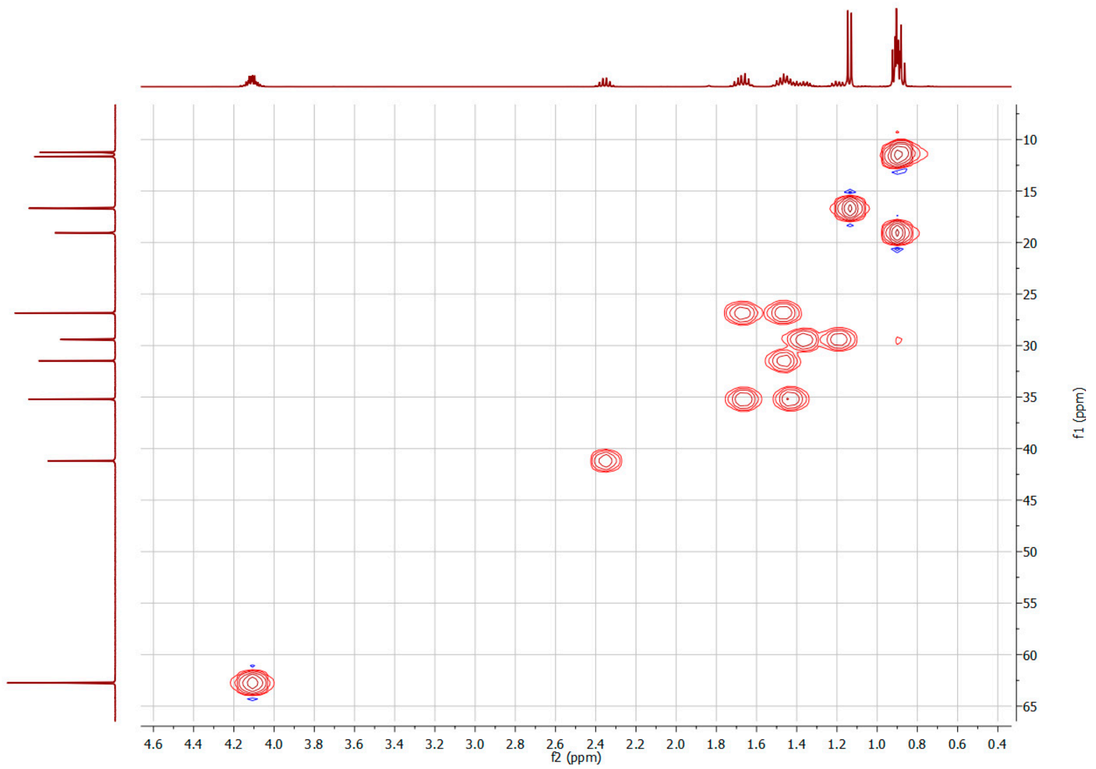

Figure S48b. HSQC spectrum of 3-methylpentyl 2-methylbutanoate in $CDCl_3$ (101 MHz)

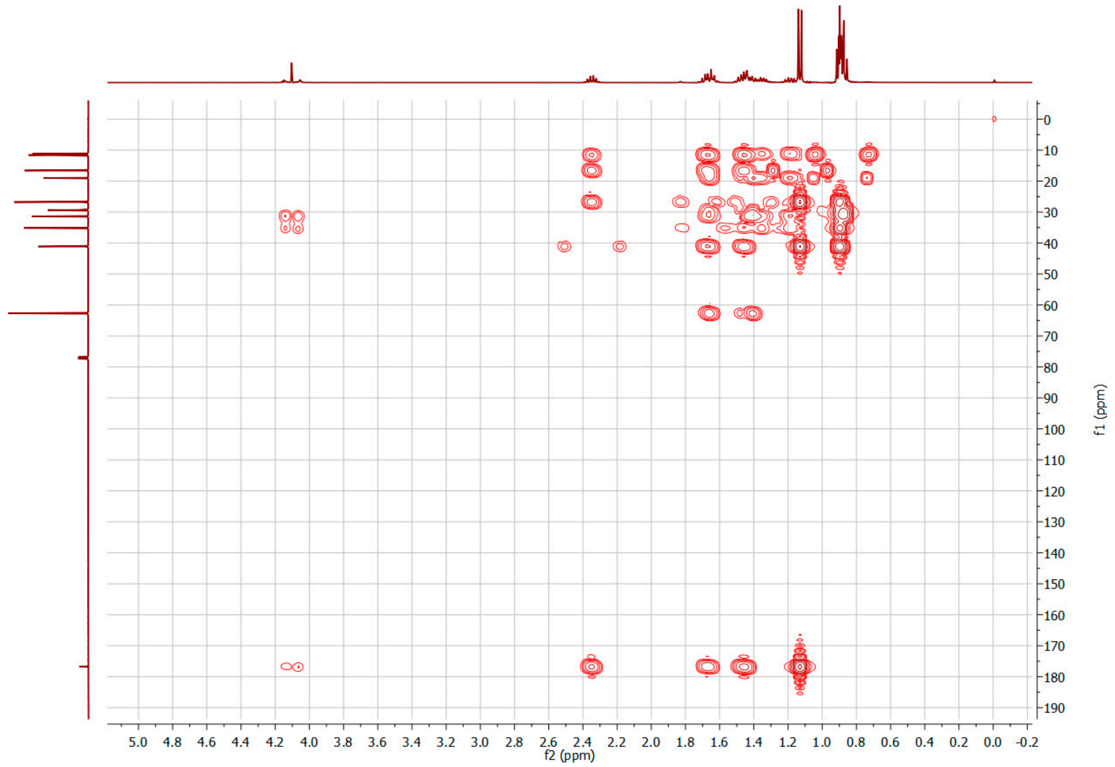

Figure S48c. HMBC spectrum of 3-methylpentyl 2-methylbutanoate in $CDCl_3$ (101 MHz)

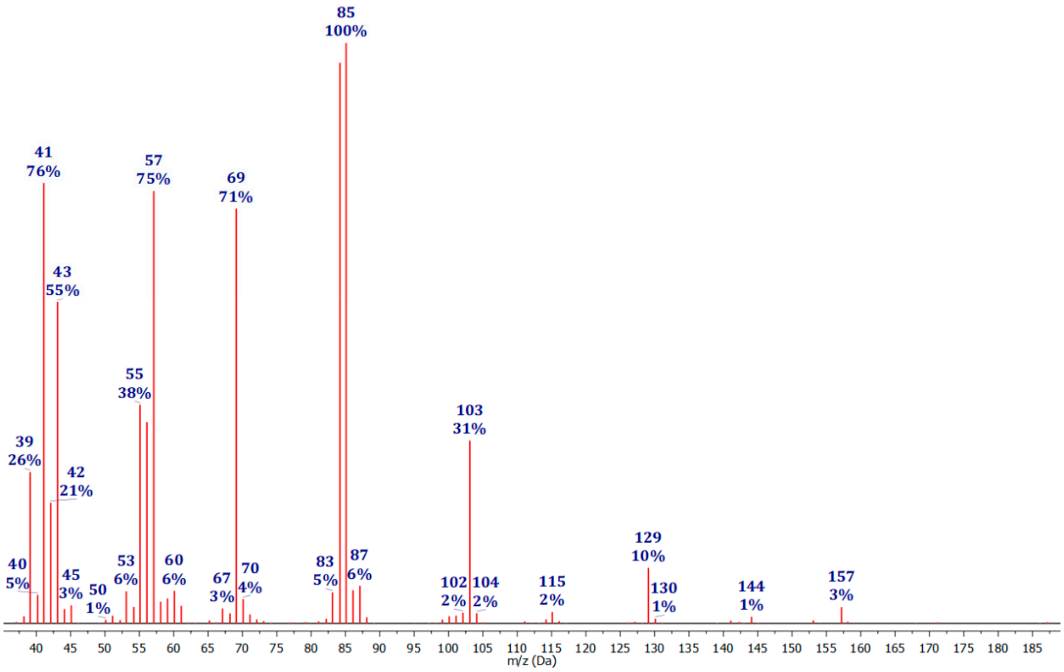

Figure S49. Mass spectrum of 3-methylpentyl 3-methylbutanoate (**6c**; RI = 1208 (DB-5MS column))

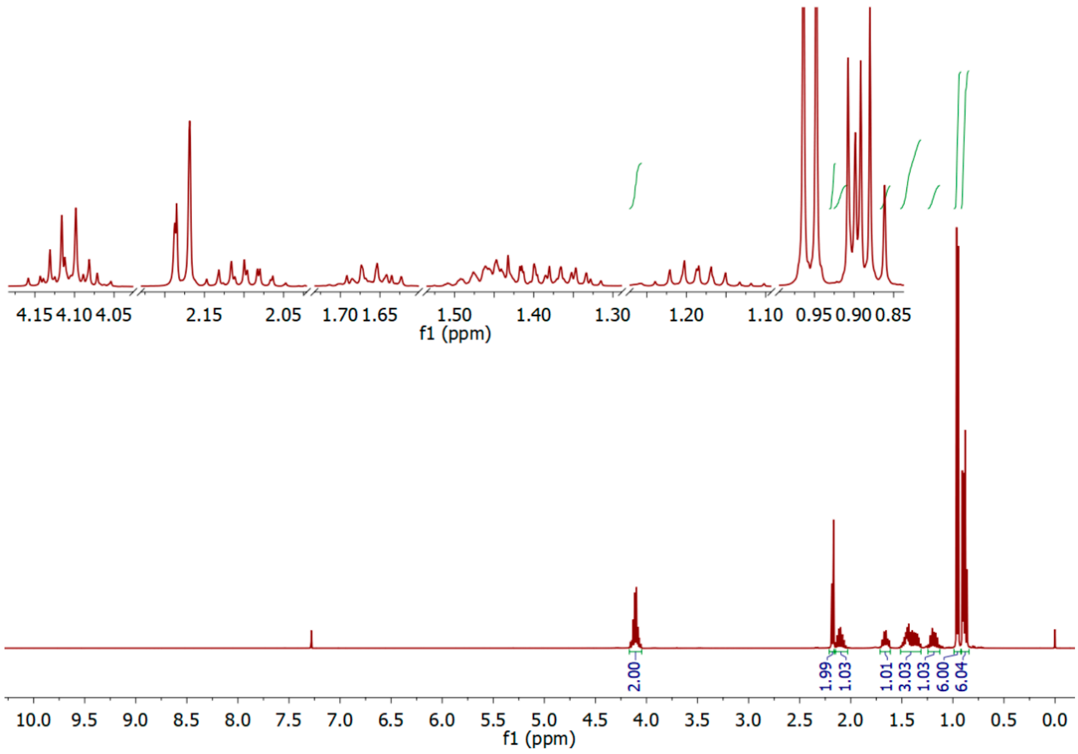

Figure S50. ^{1}H NMR spectrum of 3-methylpentyl 3-methylbutanoate in $CDCl_3$ (400 MHz)

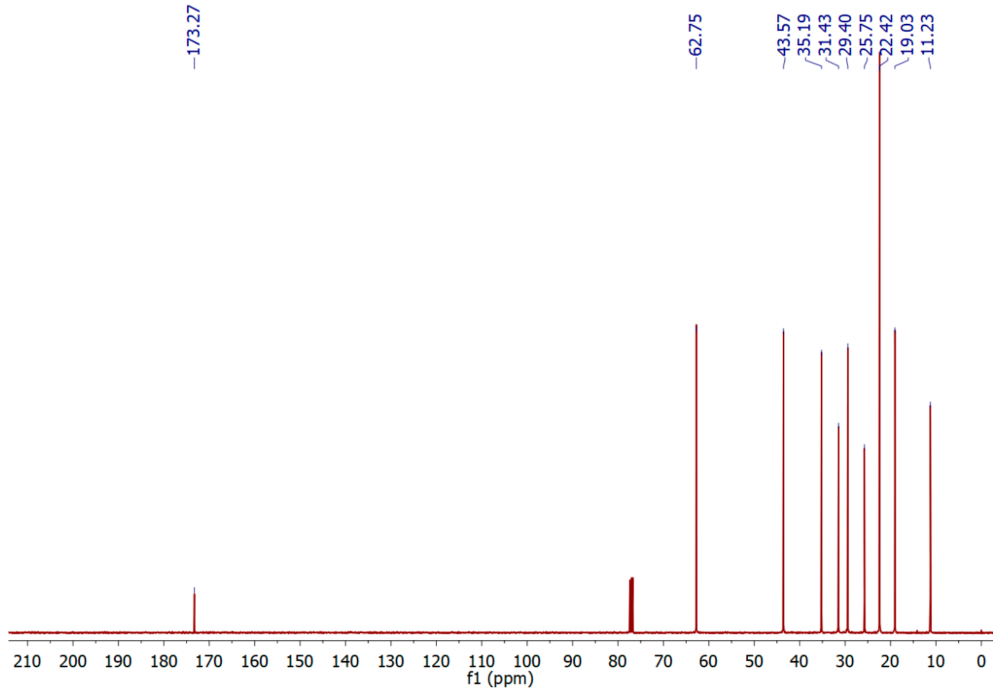

Figure S51. ^{13}C NMR spectrum of 3-methylpentyl 3-methylbutanoate in $CDCl_3$ (101 MHz)

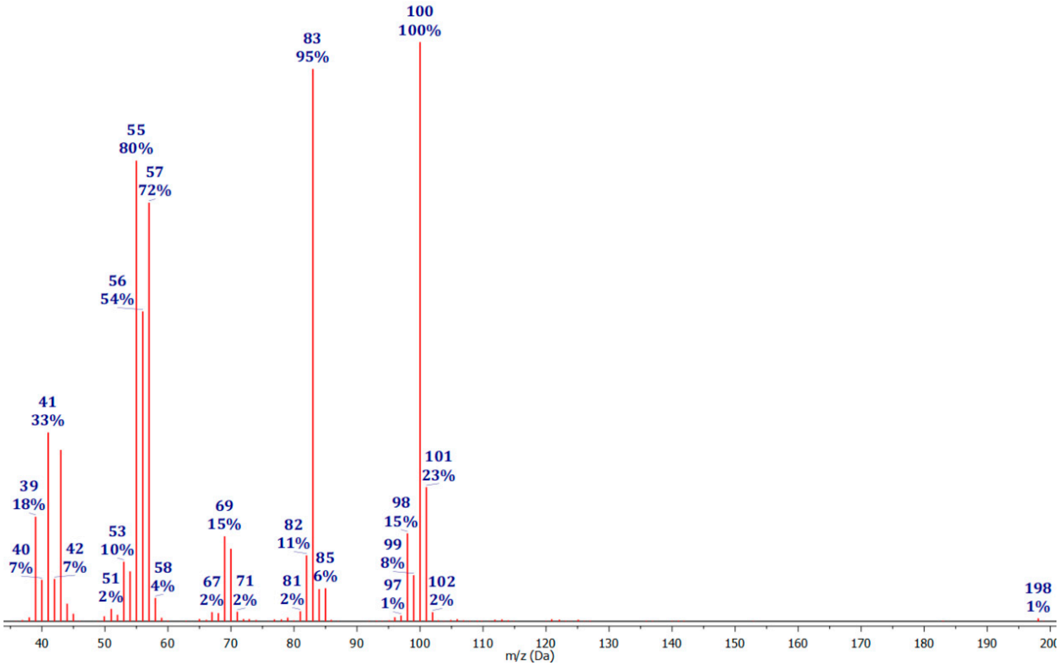

Figure S52. Mass spectrum of 4-methylhexyl angelate (**2f**; RI = 1358 (DB-5MS column))

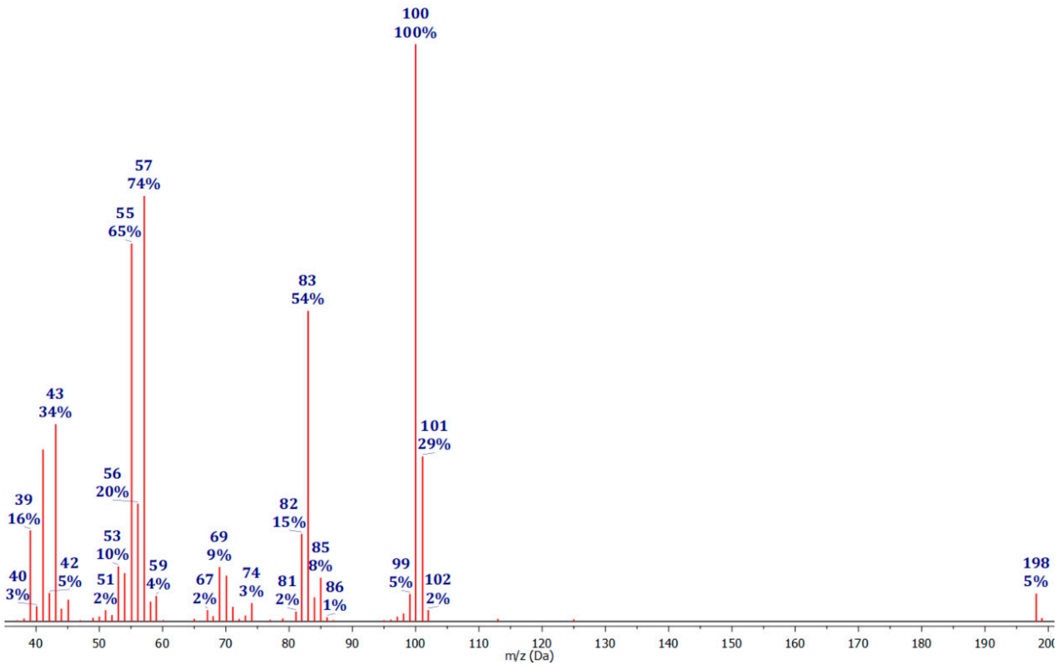

Figure S53. Mass spectrum of 5-methylhexyl angelate (**2g**; RI = 1349 (DB-5MS column))

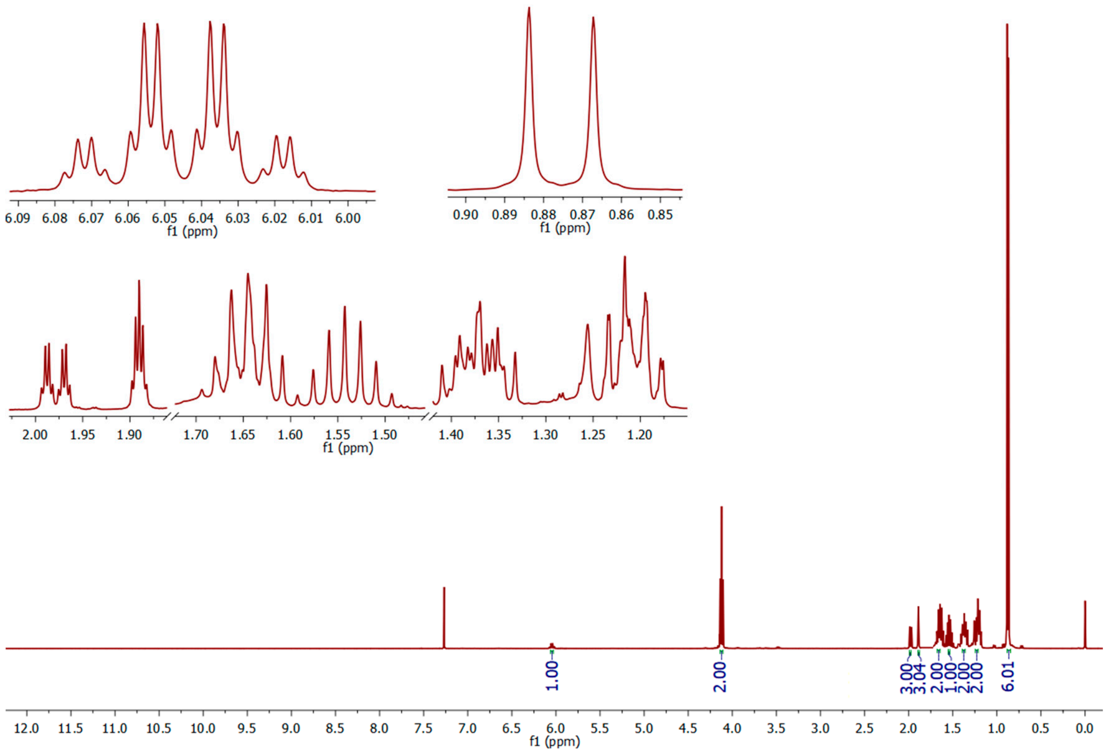

Figure S54. ^{1}H NMR spectrum of 5-methylhexyl angelate in $CDCl_3$ (400 MHz)

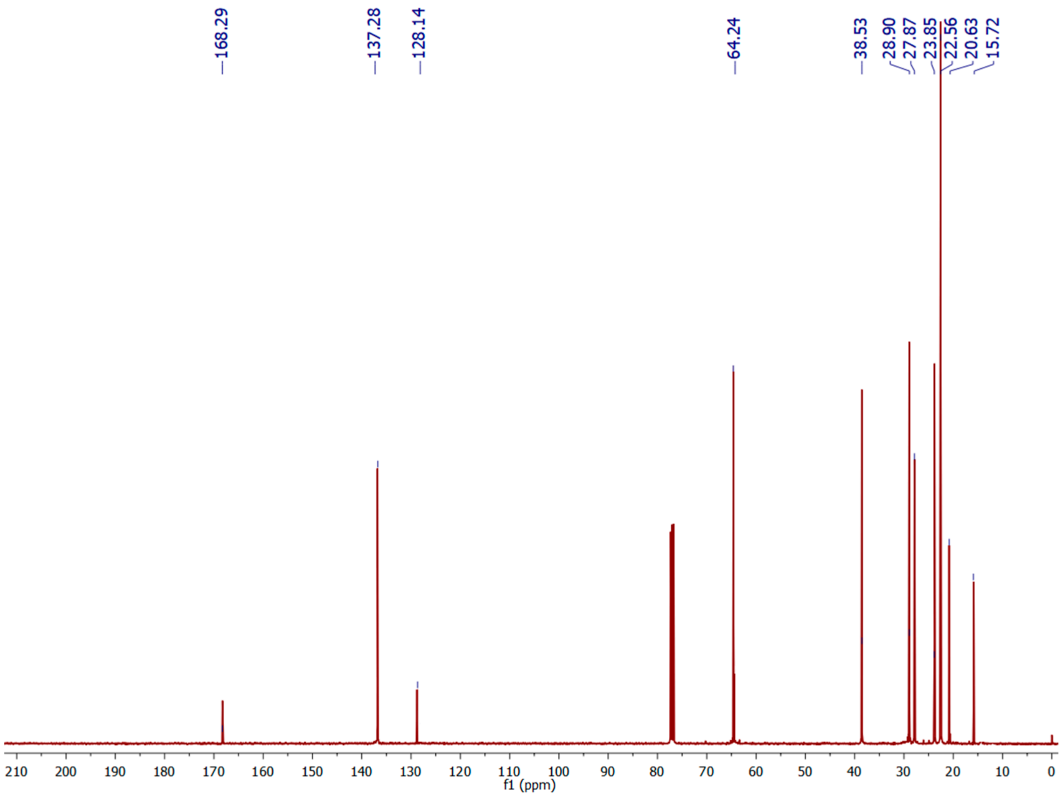

Figure S55a. ^{13}C NMR spectrum of 5-methylhexyl angelate in $CDCl_3$ (101 MHz)

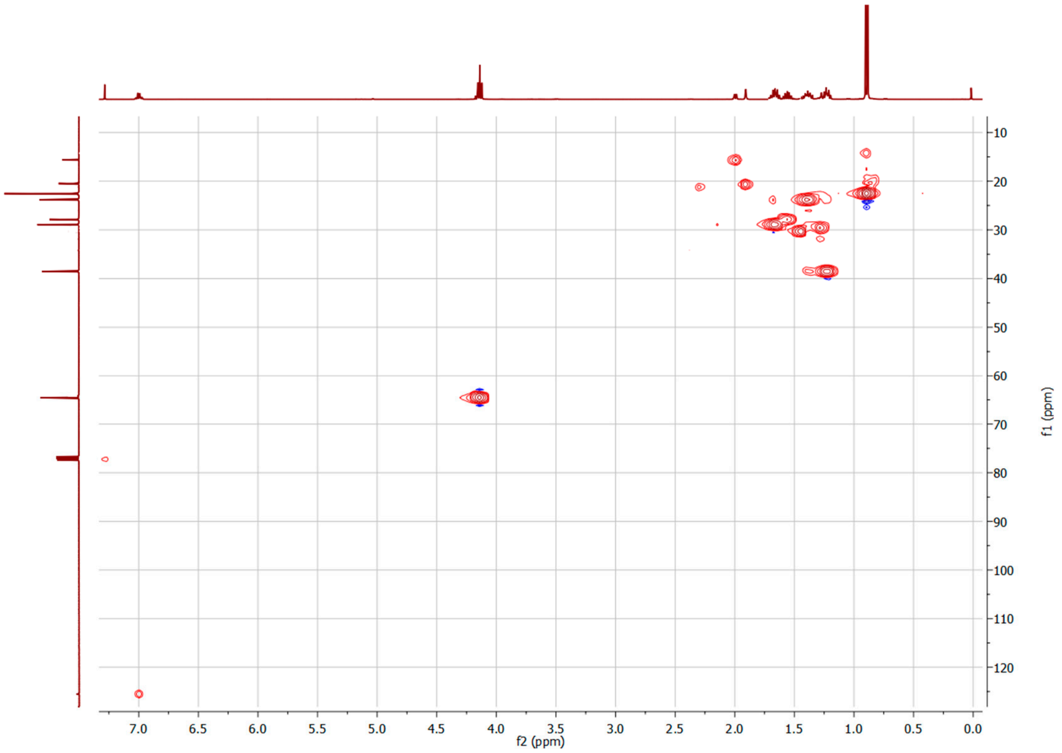

Figure S55b. HSQC spectrum of 5-methylhexyl angelate in $CDCl_3$ (101 MHz)

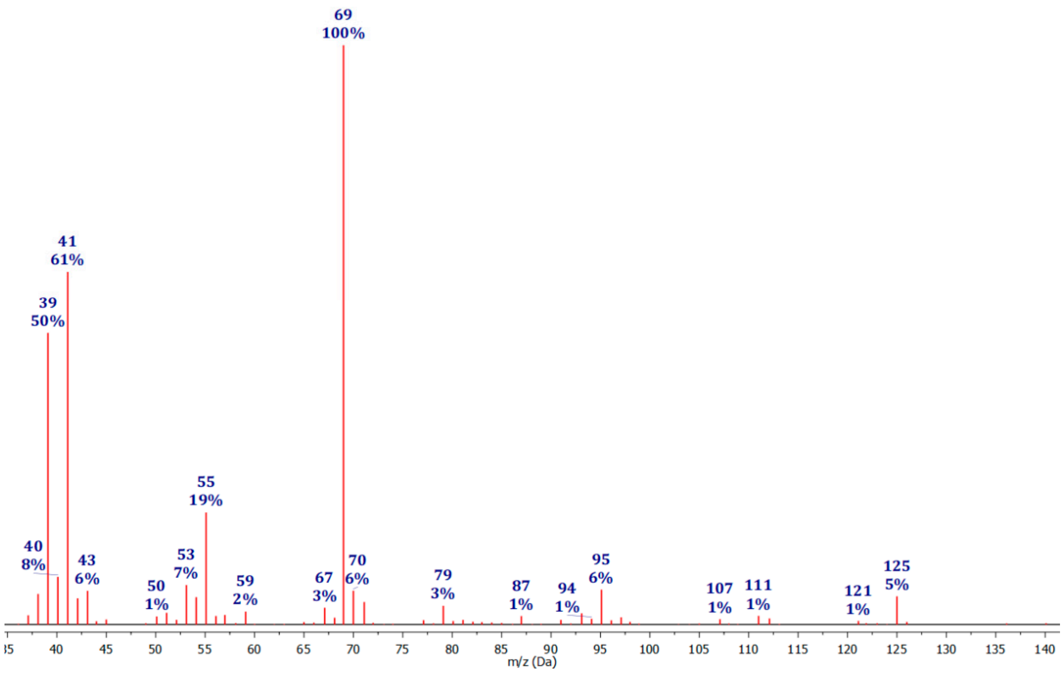

Figure S56. Mass spectrum of methallyl methacrylate (**1h**; RI = 947 (DB-5MS column))

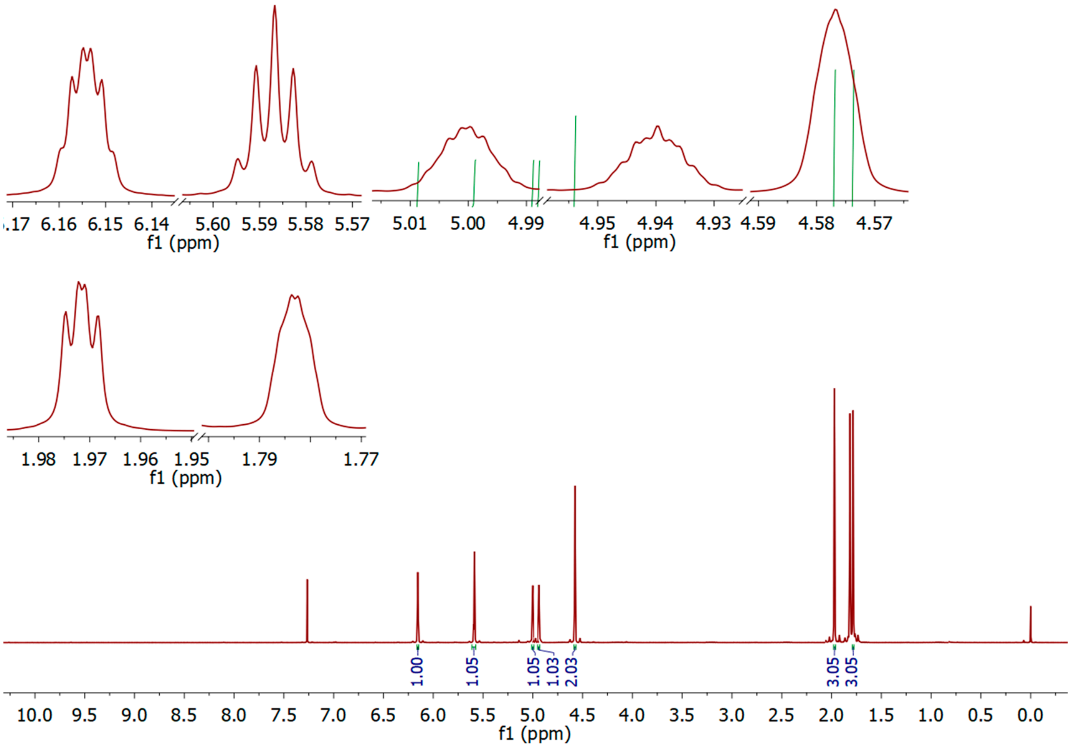

Figure S57. ^{1}H NMR spectrum of methallyl methacrylate in $CDCl_3$ (400 MHz)

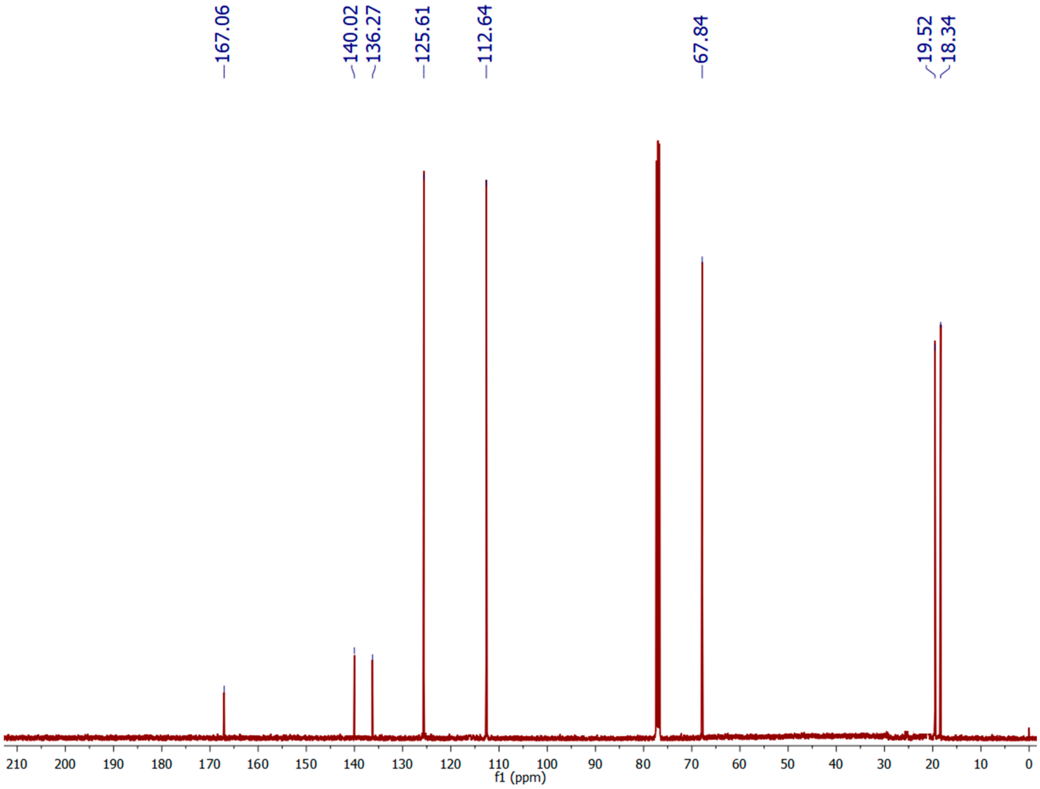

Figure S58. ^{13}C NMR spectrum of methallyl methacrylate in $CDCl_3$ (101 MHz)

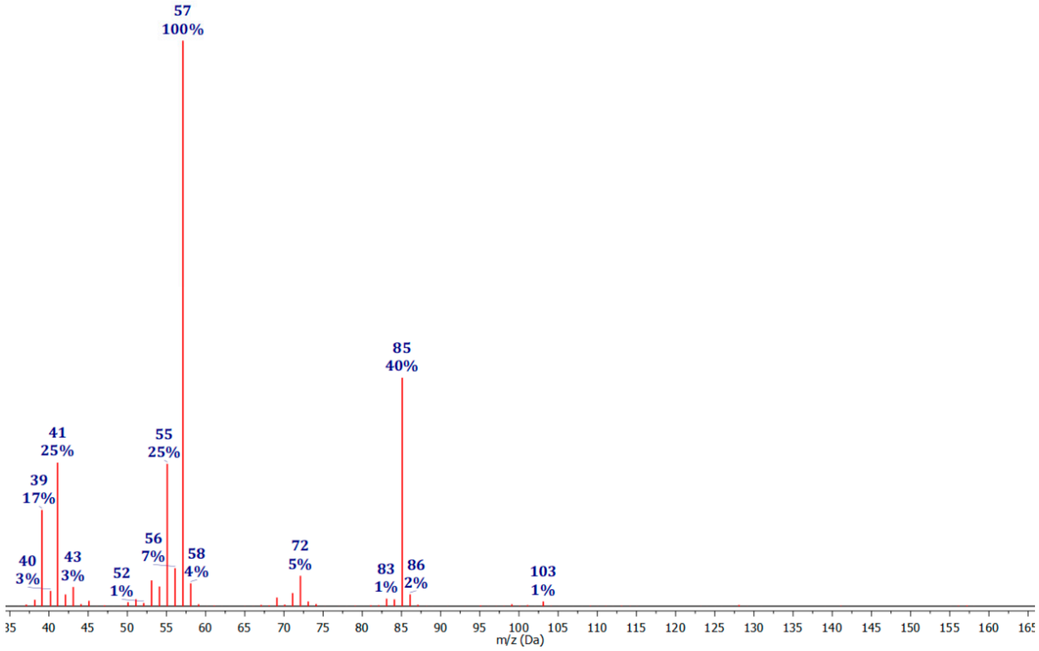

Figure S59. Mass spectrum of methallyl 2-methylbutanoate (**5h**; RI = 1015 (DB-5MS column))

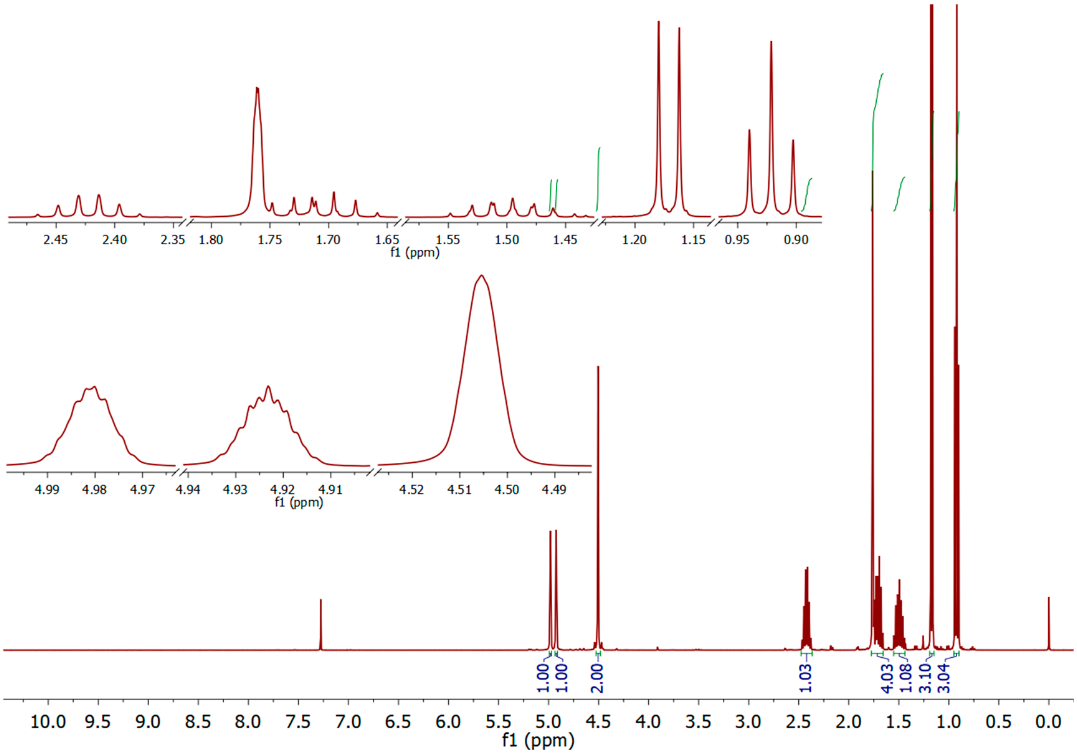

Figure S60. ^{1}H NMR spectrum of methallyl 2-methylbutanoate in $CDCl_3$ (400 MHz)

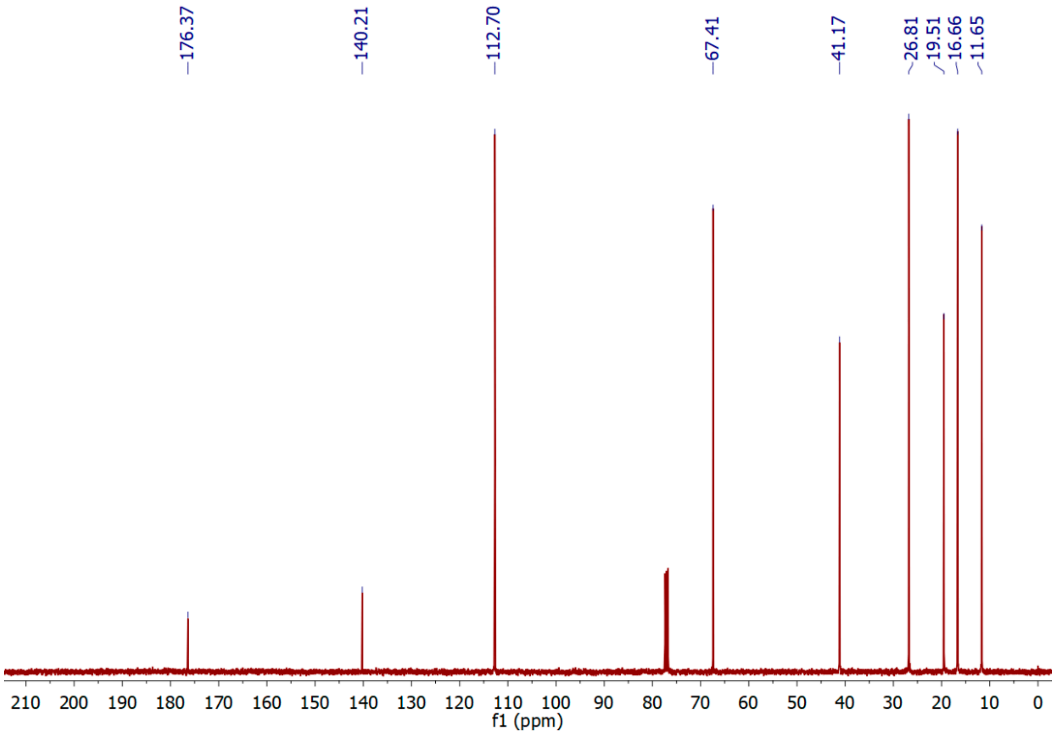

Figure S61. ^{13}C NMR spectrum of methallyl 2-methylbutanoate in $CDCl_3$ (101 MHz)

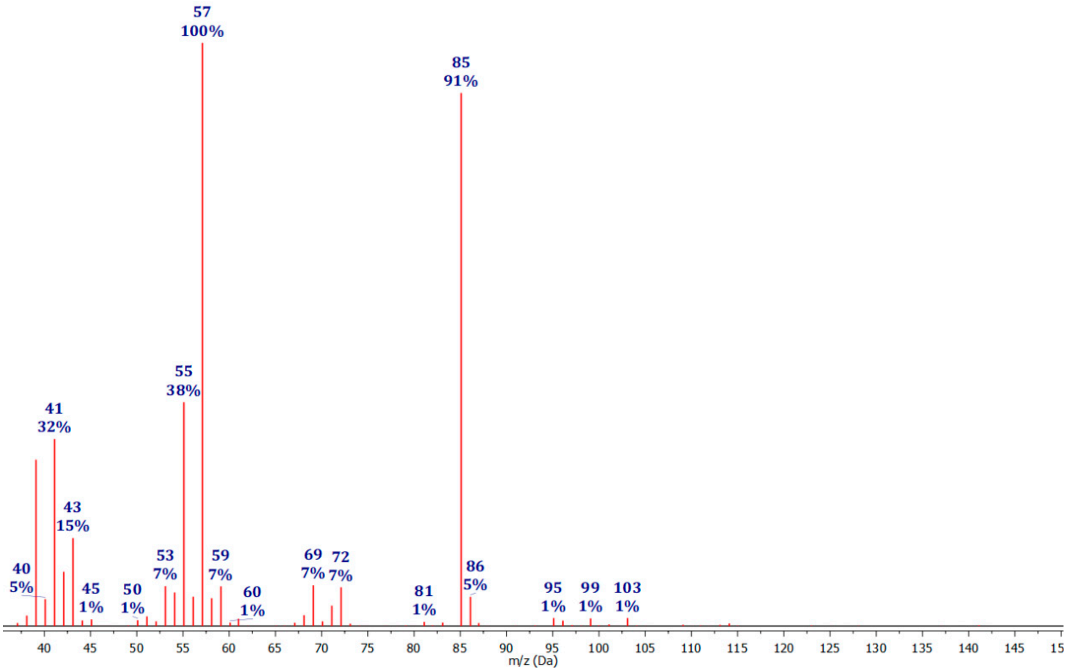

Figure S62. Mass spectrum of methallyl 3-methylbutanoate (**6h**; RI = 1017 (DB-5MS column))

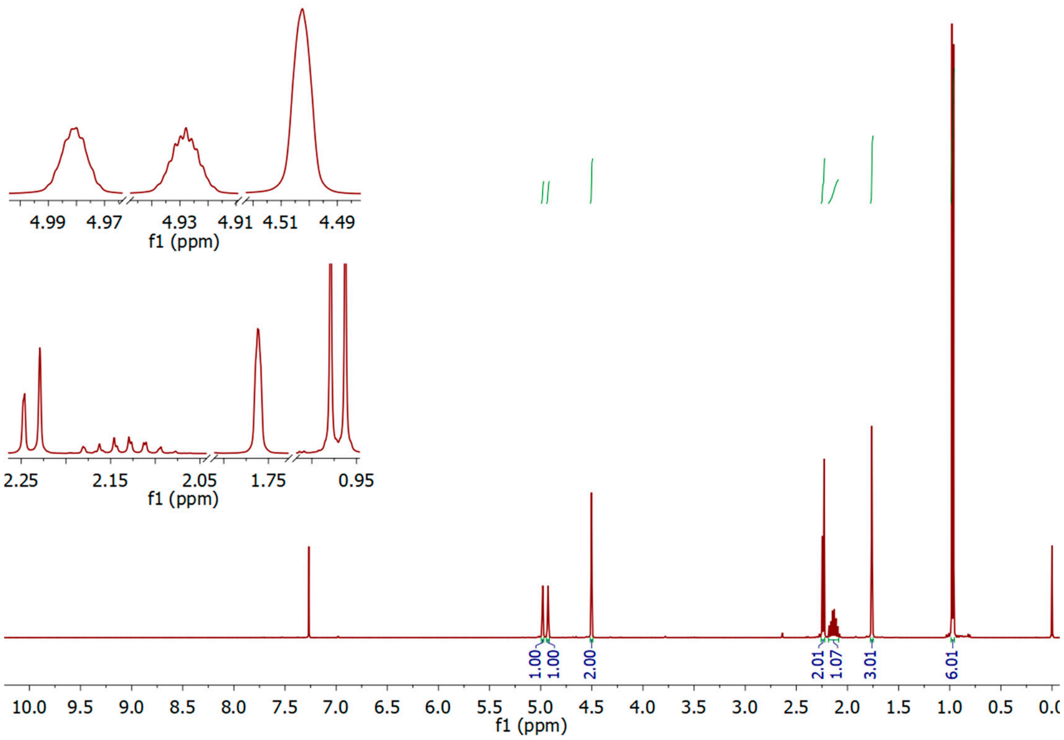

Figure S63. ^{1}H NMR spectrum of methallyl 3-methylbutanoate in $CDCl_3$ (400 MHz)

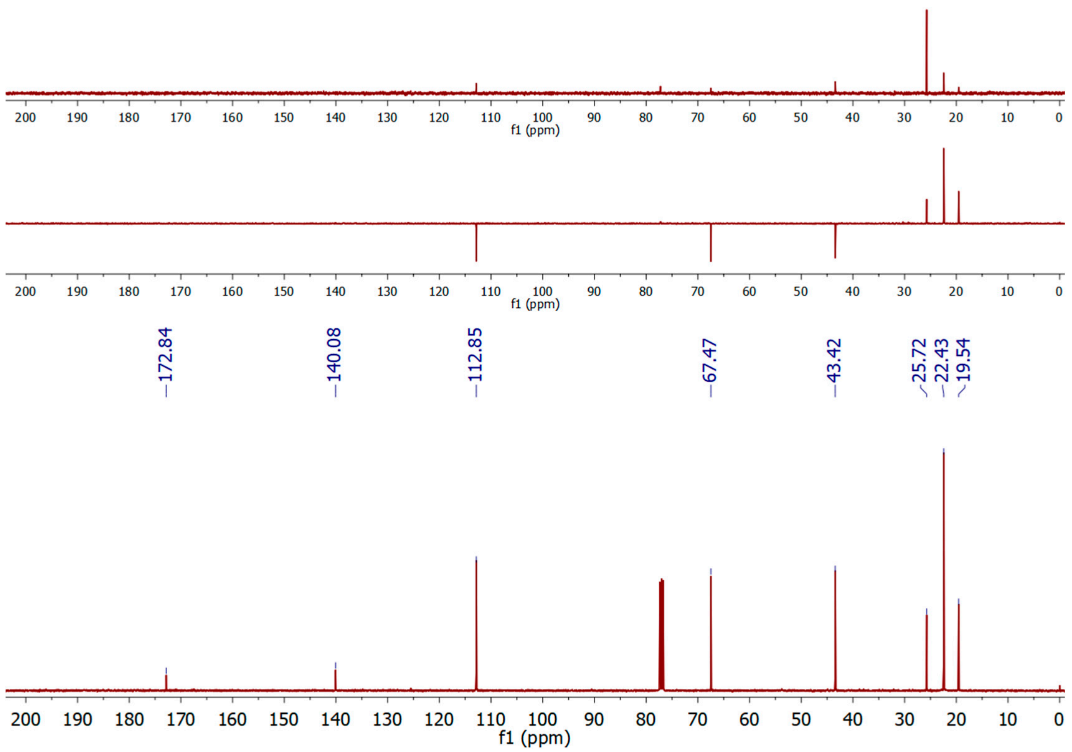

Figure S64a. DEPT-90, DEPT-135, and ^{13}C NMR spectra (from top to bottom) of methallyl 3-methylbutanoate in $CDCl_3$ (101 MHz)

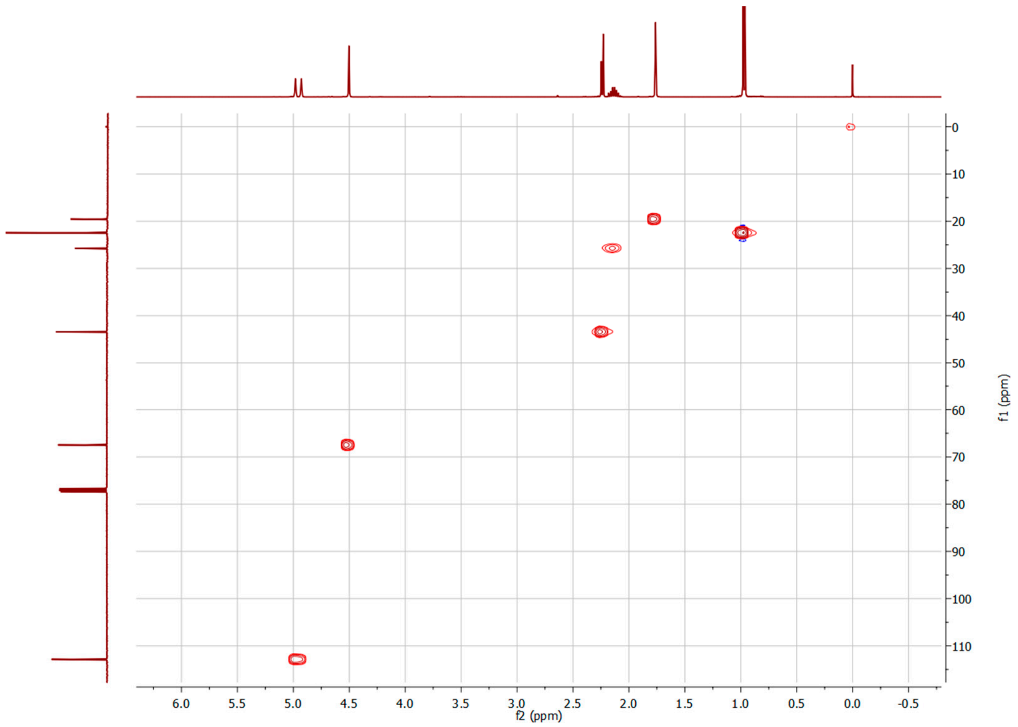

Figure S64b. HSQC spectrum of methallyl 3-methylbutanoate in $CDCl_3$ (101 MHz)

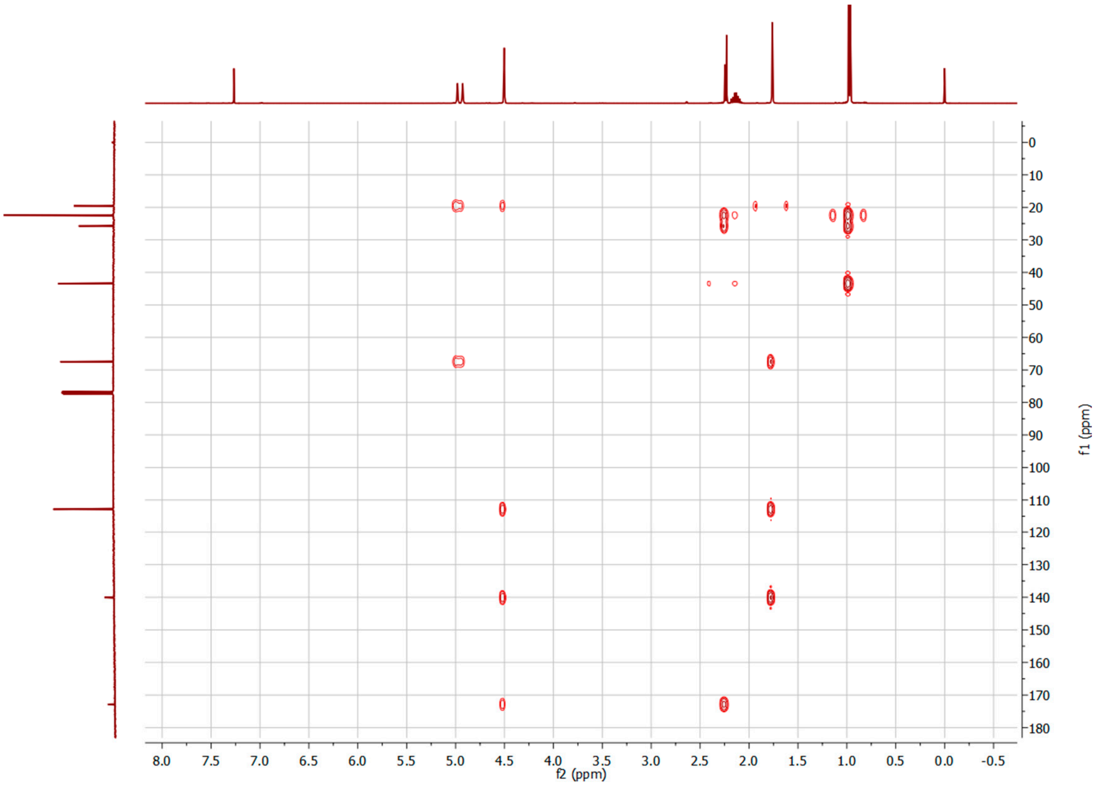

Figure S64c. HMBC spectrum of methallyl 3-methylbutanoate in $CDCl_3$ (101 MHz)

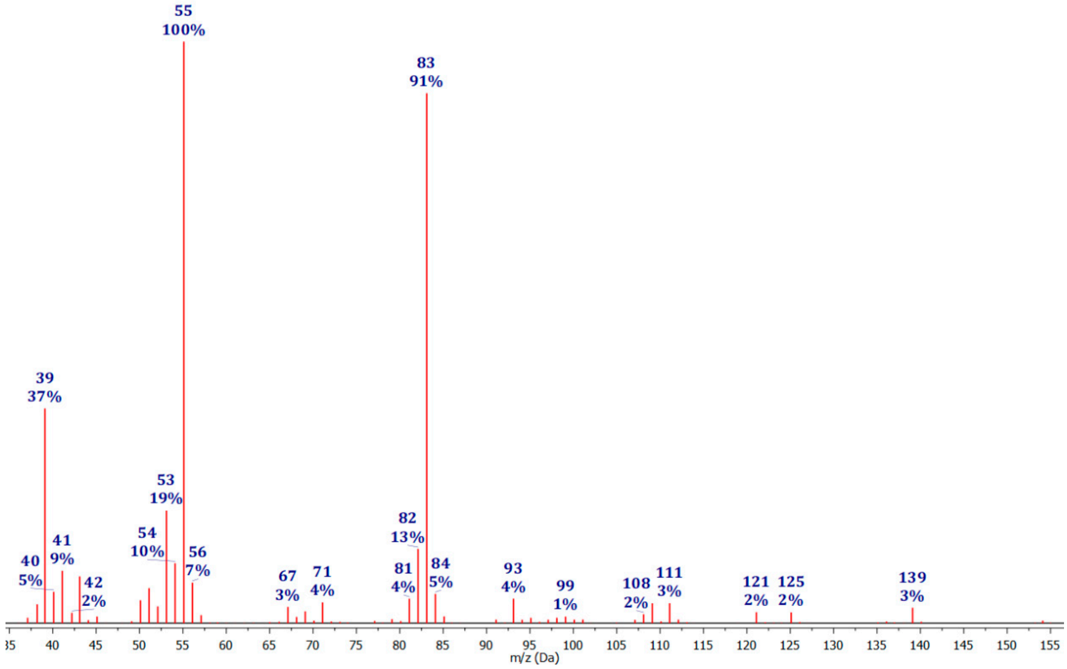

Figure S65. Mass spectrum of methallyl angelate (**2h**; RI = 1062 (DB-5MS column))

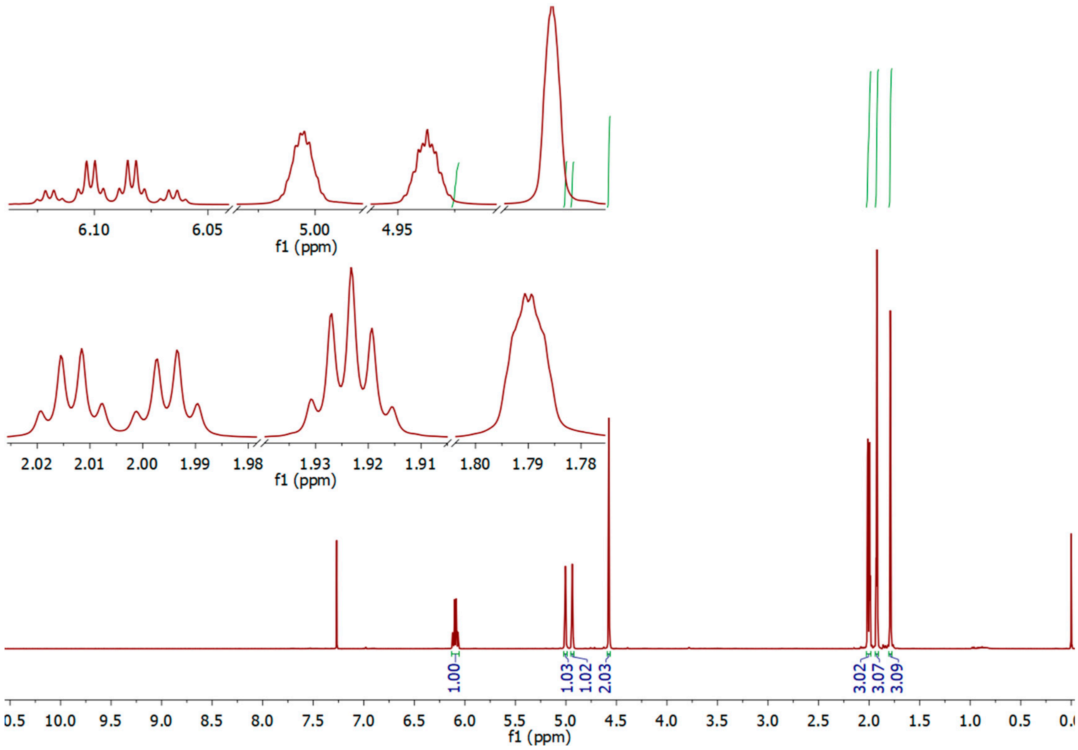

Figure S66. ^{1}H NMR spectrum of methallyl angelate in $CDCl_3$ (400 MHz)

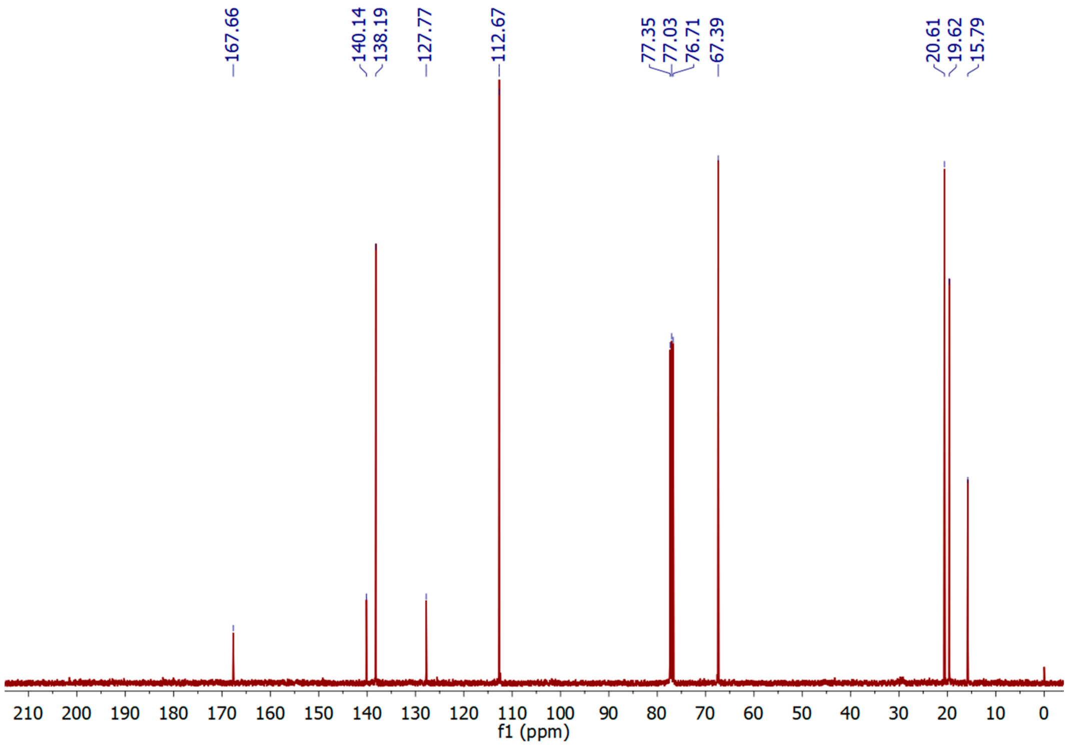

Figure S67. ^{13}C NMR spectrum of methallyl angelate in $CDCl_3$ (101 MHz)

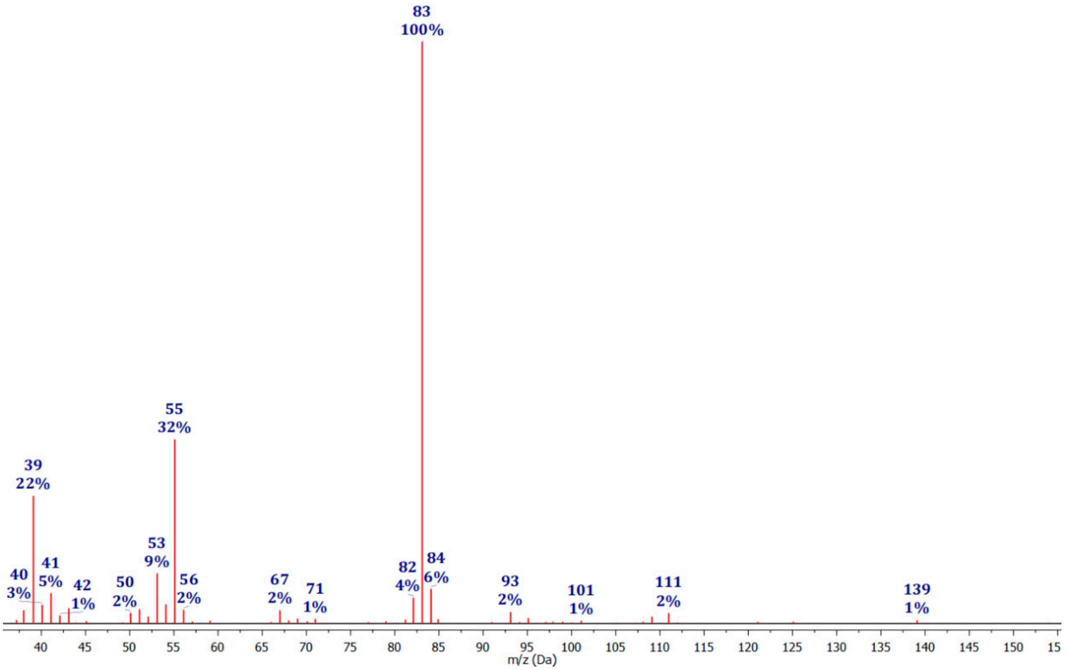

Figure S68. Mass spectrum of methallyl senecioate (**3h**; RI = 1091 (DB-5MS column))

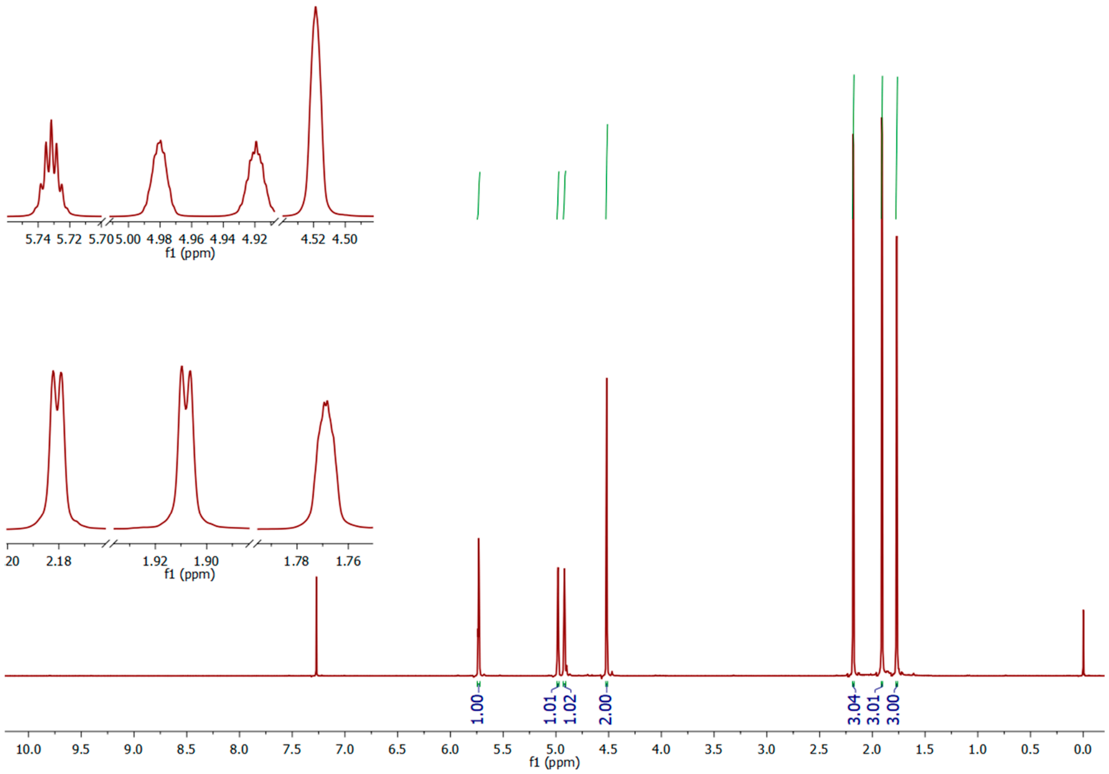

Figure S69. ^{1}H NMR spectrum of methallyl senecioate in $CDCl_3$ (400 MHz)

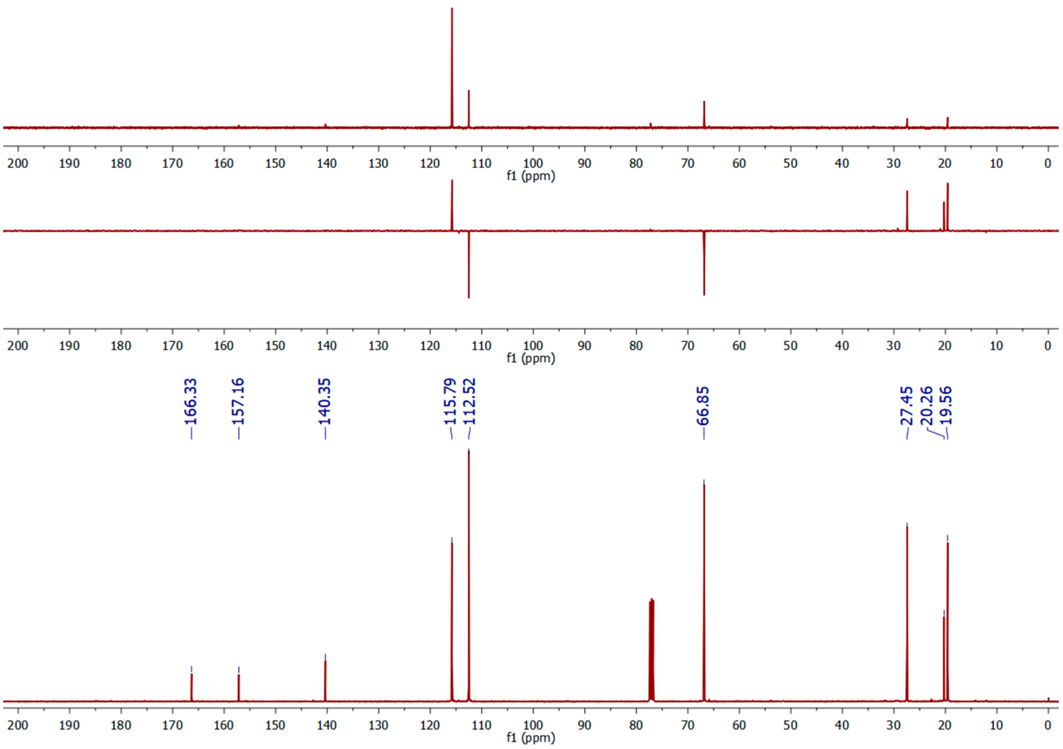

Figure S70a. DEPT-90, DEPT-135, and ^{13}C NMR spectra (from top to bottom) of methallyl senecioate in $CDCl_3$ (101 MHz)

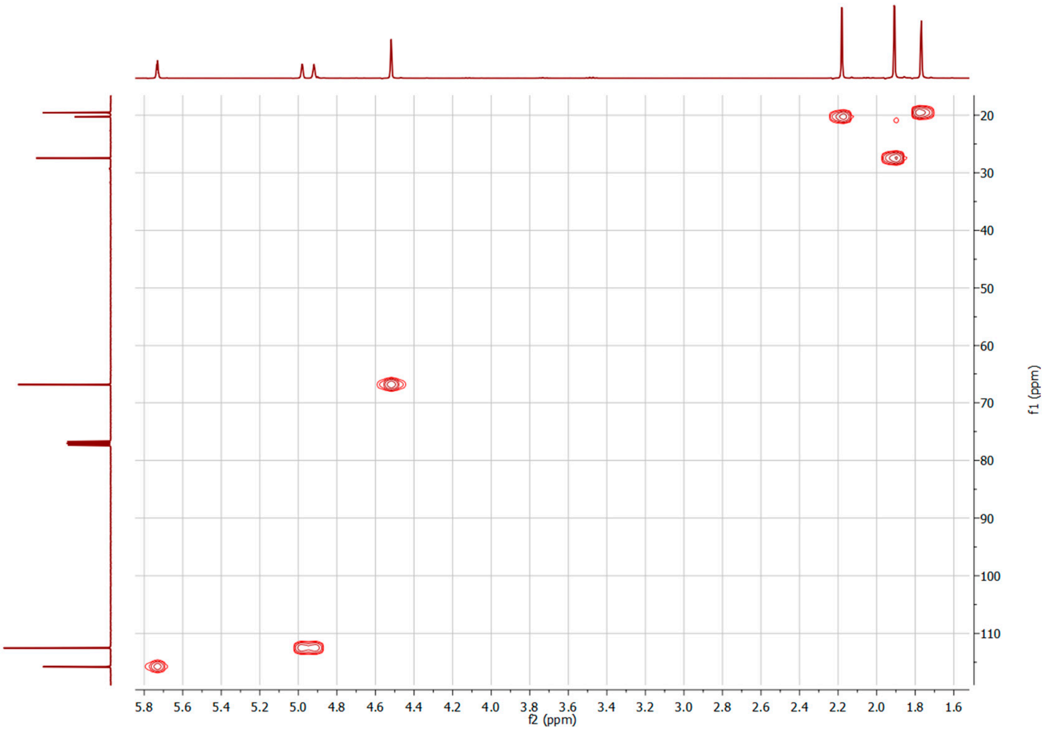

Figure S70b. HSQC spectrum of methallyl senecioate in $CDCl_3$ (101 MHz)

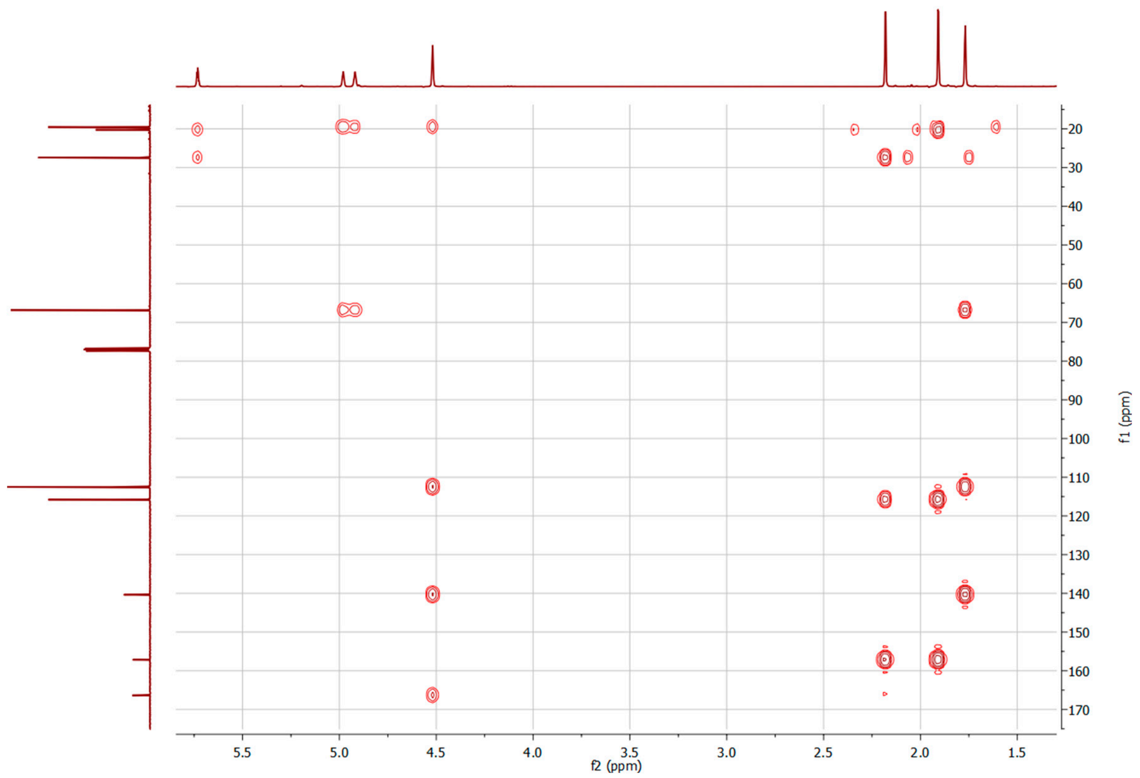

Figure S70c. HMBC spectrum of methallyl senecioate in $CDCl_3$ (101 MHz)

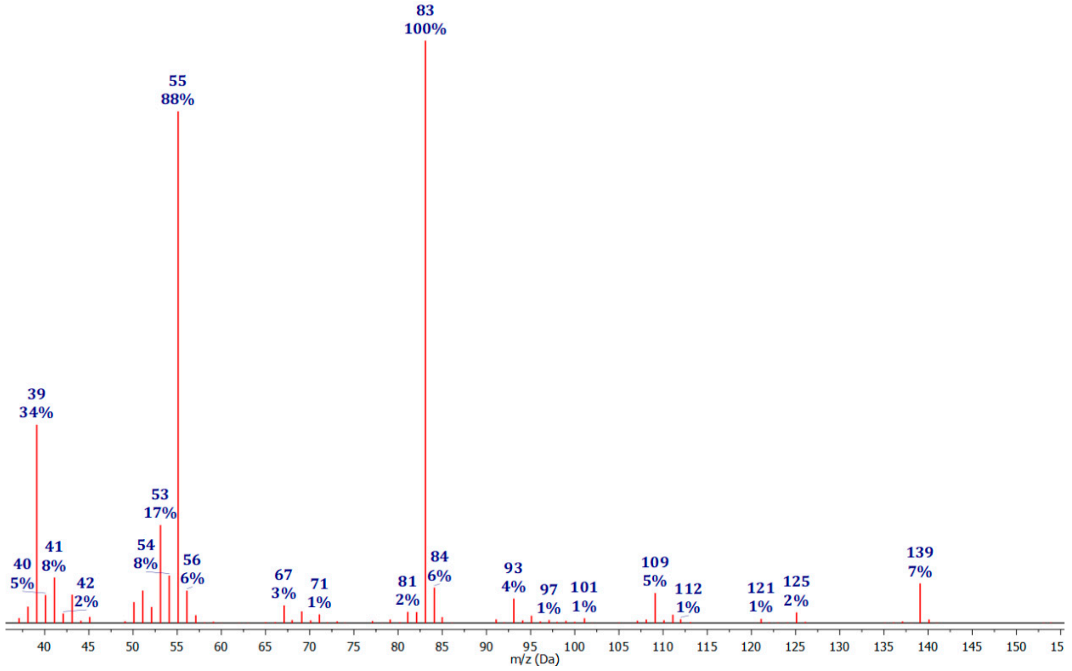

Figure S71. Mass spectrum of methallyl tiglate (**4h**; RI = 1106 (DB-5MS column))

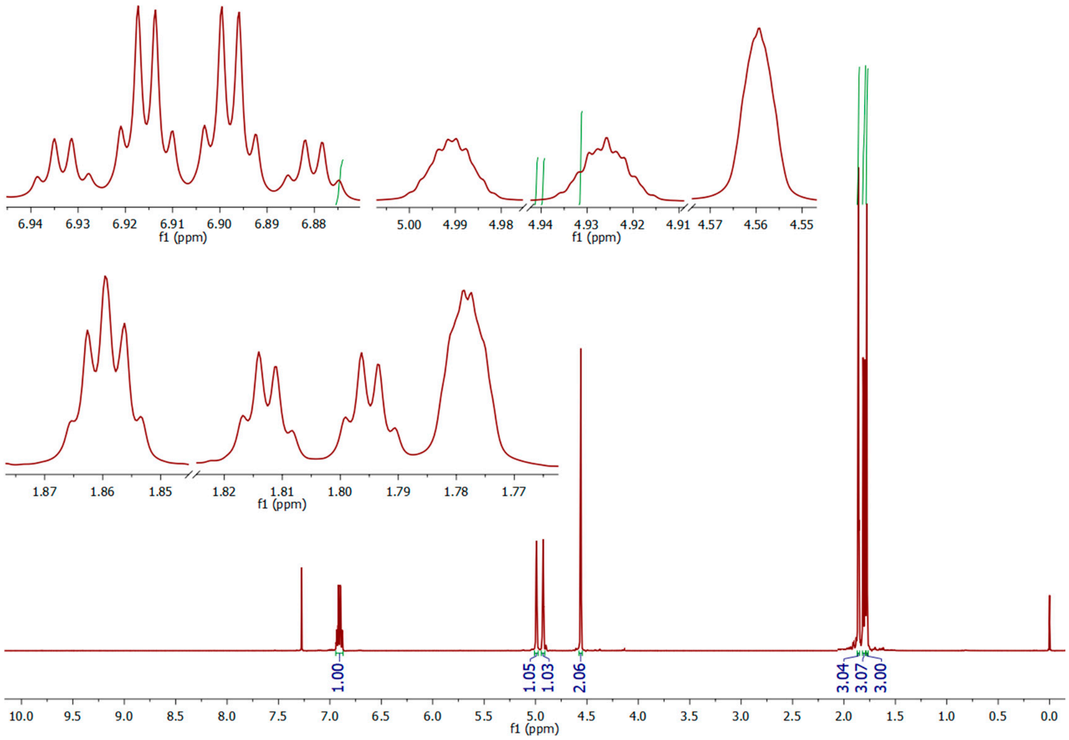

Figure S72. ^{1}H NMR spectrum of methallyl tiglate in $CDCl_3$ (400 MHz)

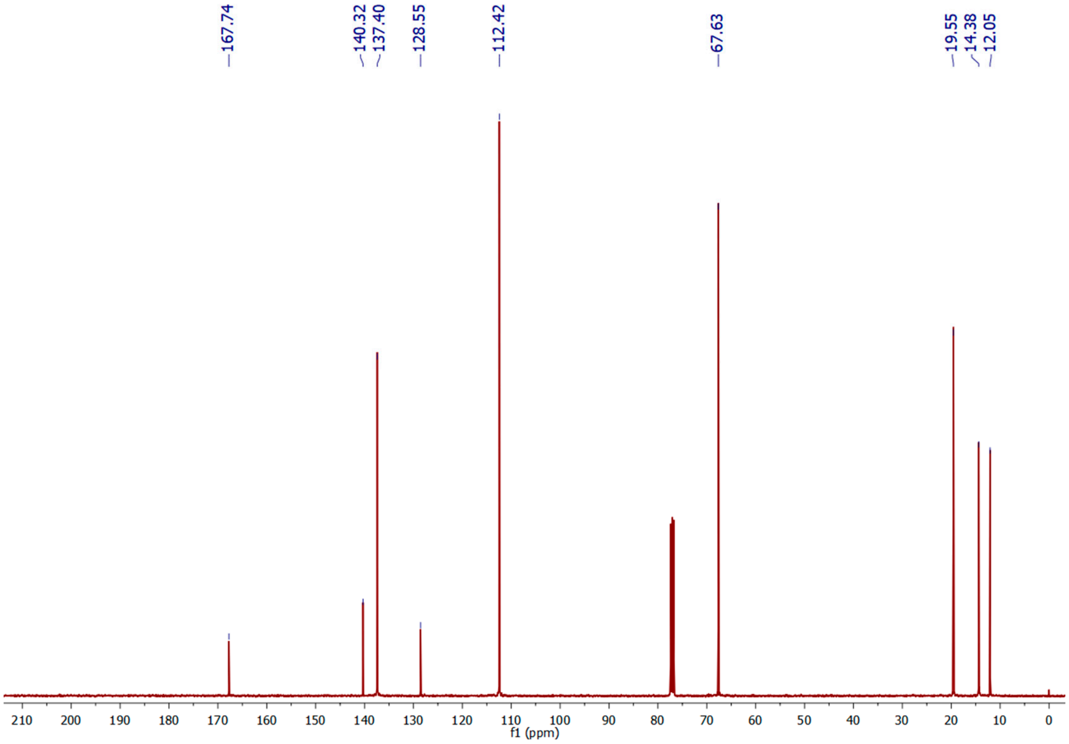

Figure S73. ^{13}C NMR spectrum of methallyl tiglate in $CDCl_3$ (101 MHz)

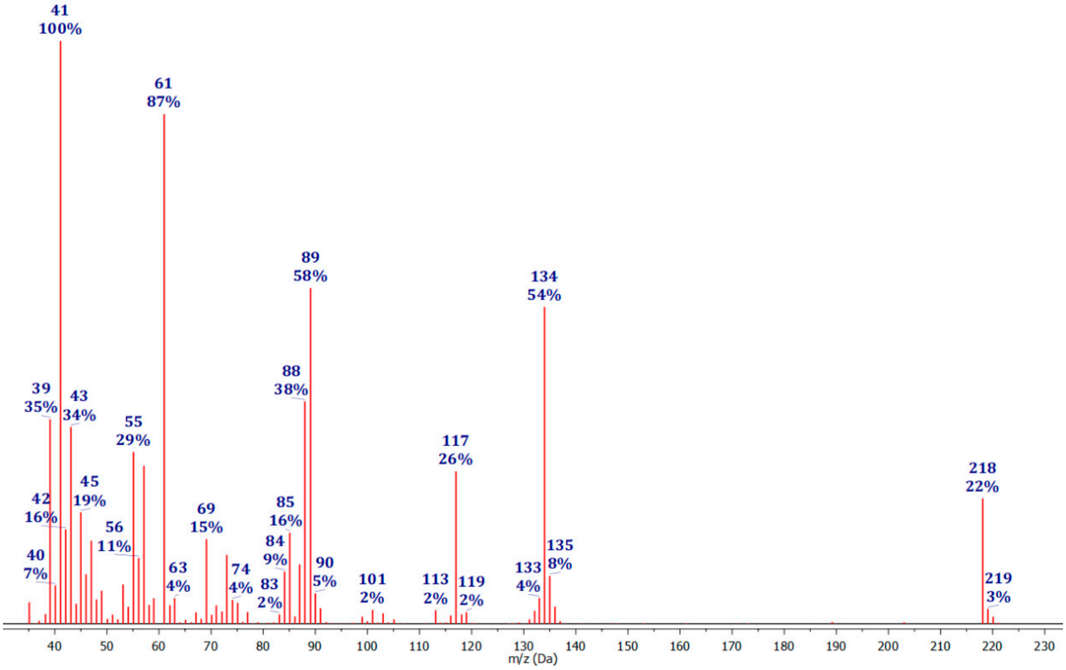

Figure S74. Mass spectrum of 3-methylpentyl 2-methyl-3-(methylthio)propanoate (**7c**; RI = 1493 (DB-5MS column))

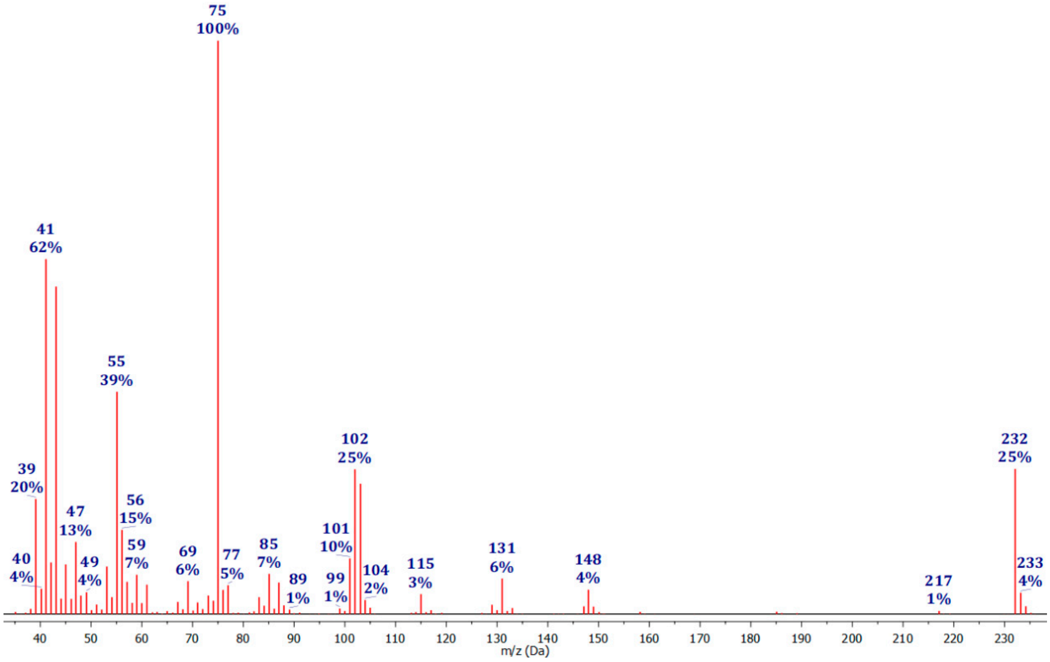

Figure S75. Mass spectrum of 3-methylpentyl 2-methyl-3-(methylthio)butanoate (**8c**; RI = 1550 (DB-5MS column))

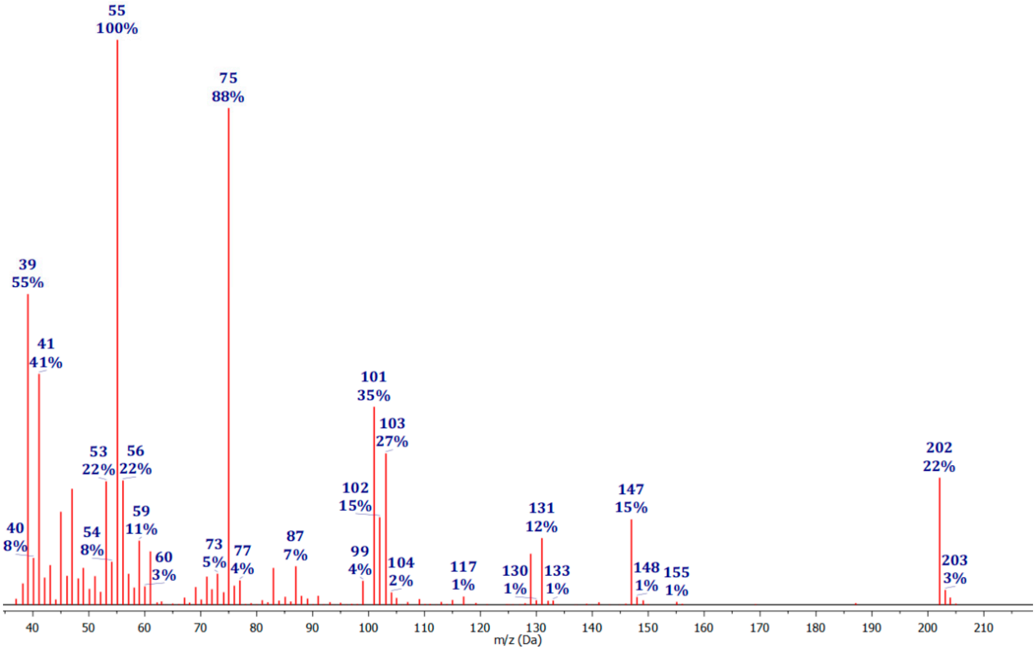

Figure S76. Mass spectrum of 2-methylallyl 2-methyl-3-(methylthio)butanoate (**8h**; RI = 1358 (DB-5MS column))

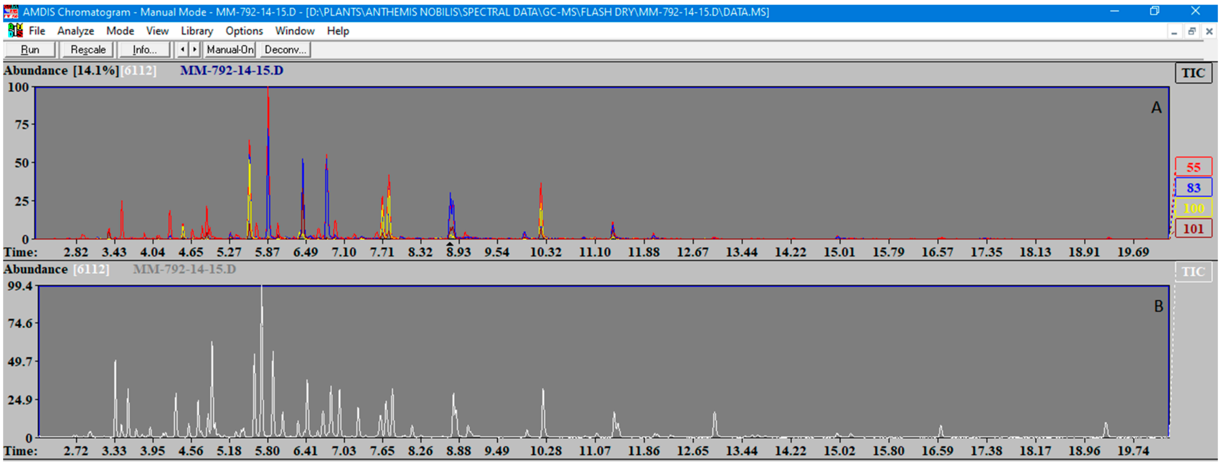

Figure S77. (A) Extracted partial ion chromatograms (*m/z* 55, 83, 100, and 101) highlighting diagnostic fragments for the identification of ester series, and (B) representative total ion chromatogram (TIC) of the ester-enriched essential oil fraction

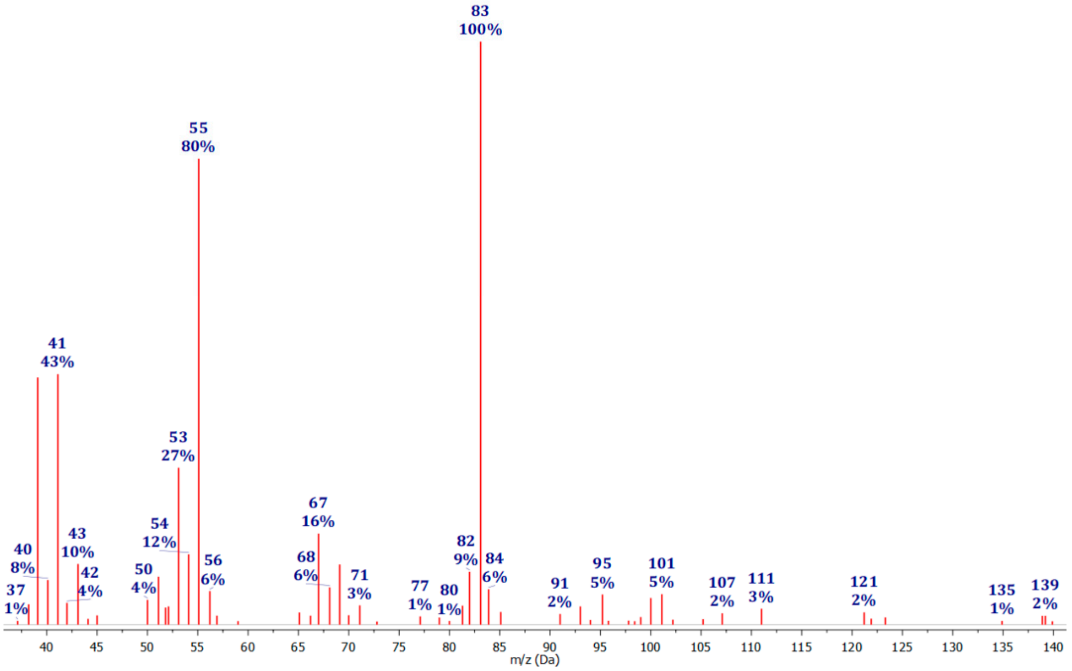

Figure S78. Mass spectrum of tentatively identified (*Z*)-2-methylbut-2-en-1-yl angelate (RI = 1157 (DB-5MS column))

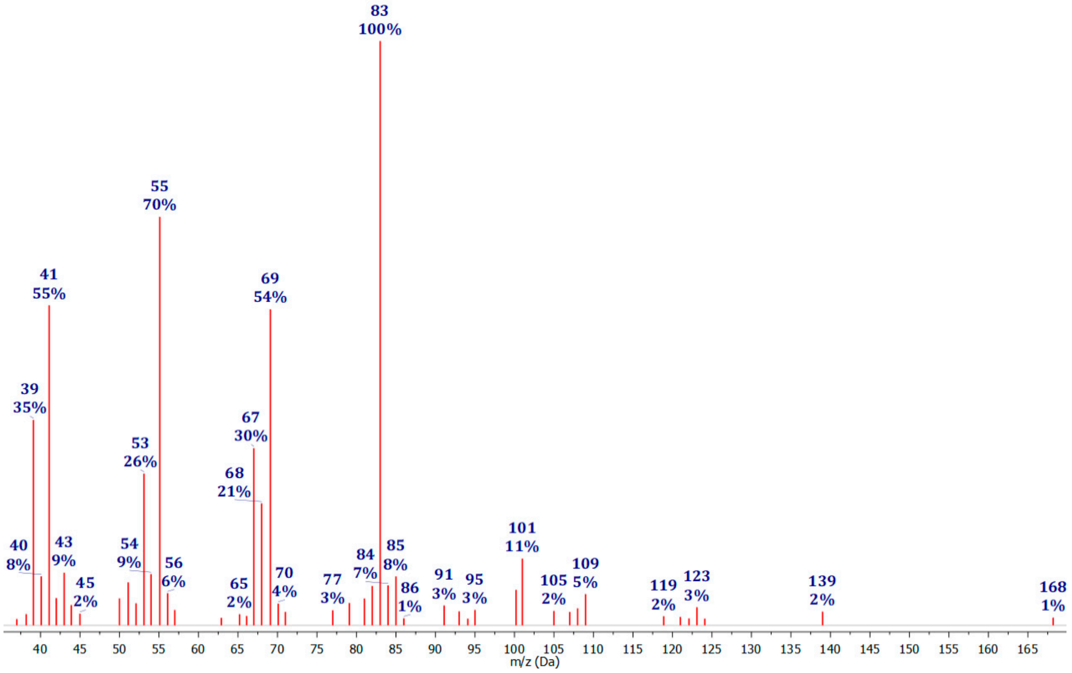

Figure S79. Mass spectrum of unidentified constituent 1 (RI = 1169 (DB-5MS column))

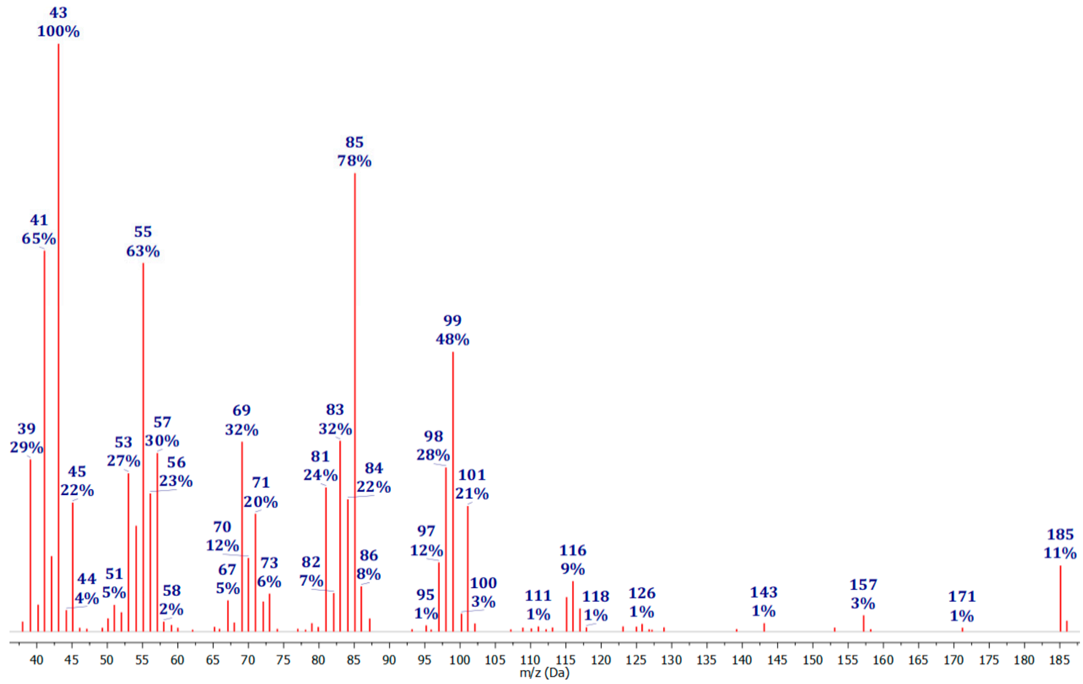

Figure S80. Mass spectrum of unidentified constituent 2 (RI = 1386 (DB-5MS column))

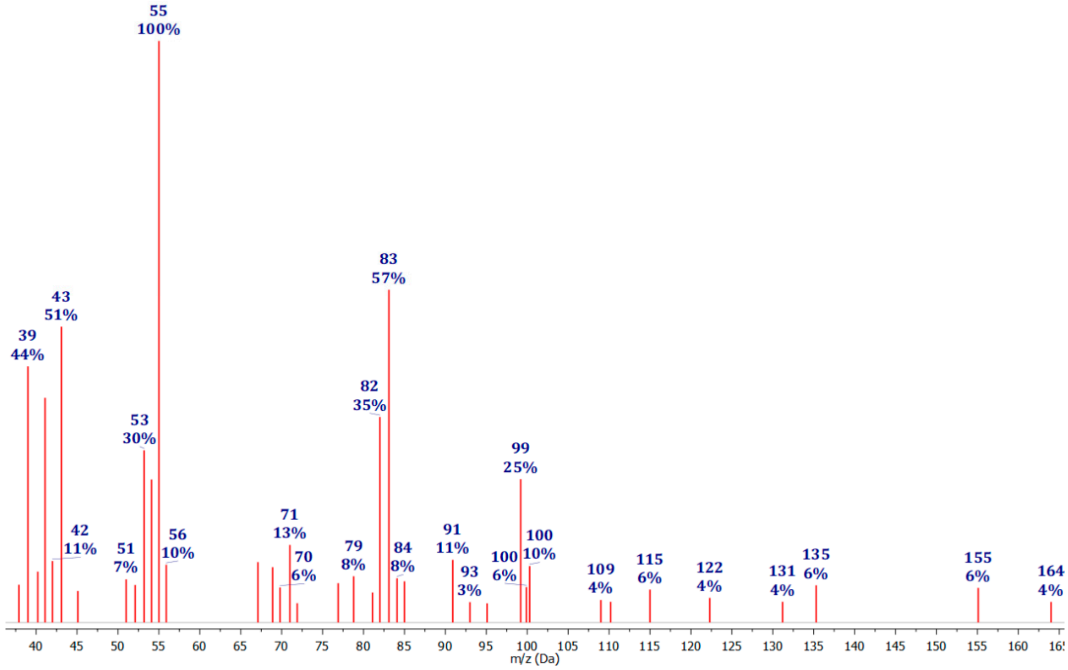

Figure S81. Mass spectrum of unidentified constituent 3 (RI = 1401 (DB-5MS column))

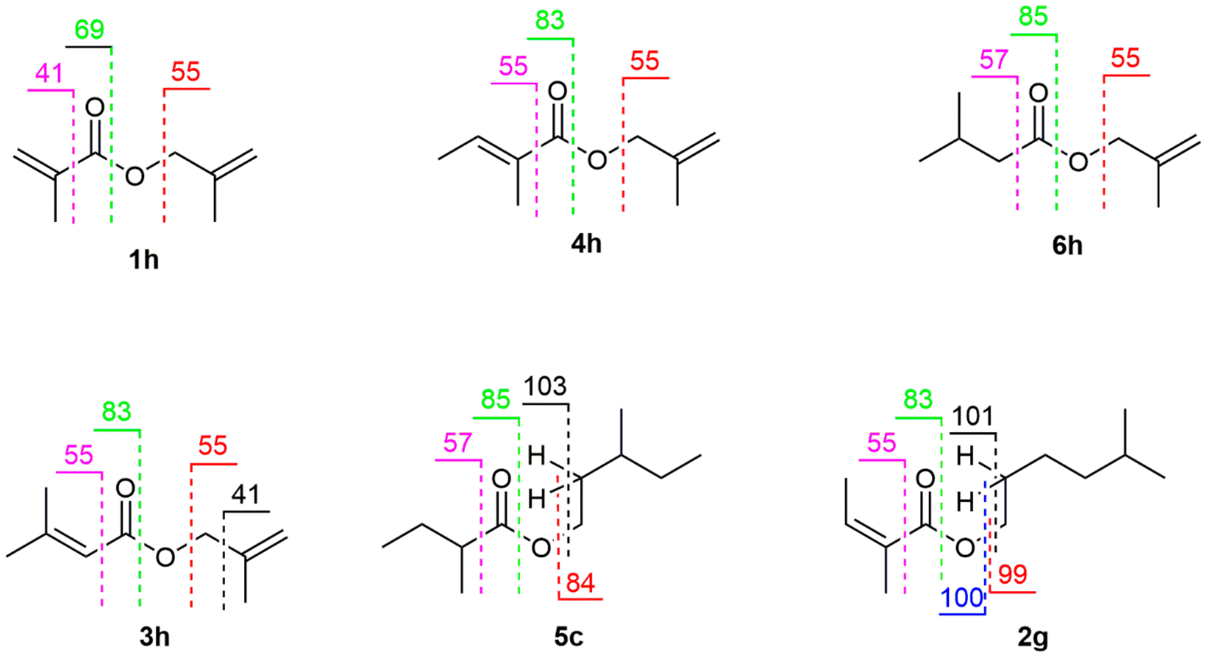

Figure S82. (EI)MS fragmentation patterns of selected rare natural products (methallyl methacrylate (**1h**) and methallyl tiglate (**4h**)) and newly identified natural products (methallyl 3-methylbutanoate (**6h**), methallyl senecioate (**3h**), 3-methylpentyl 2-methylbutanoate (**5c**), and 5-methylhexyl angelate (**2g**))
